# Generalized sequential state discrimination for multiparty QKD and its optical implementation

**DOI:** 10.1038/s41598-020-63719-9

**Published:** 2020-05-19

**Authors:** Min Namkung, Younghun Kwon

**Affiliations:** Department of Applied Physics, Hanyang University, Ansan, Kyunggi-Do 425-791 South Korea

**Keywords:** Quantum information, Quantum mechanics

## Abstract

Sequential state discrimination is a strategy for *N* separated receivers. As sequential state discrimination can be applied to multiparty quantum key distribution (QKD), it has become one of the relevant research fields in quantum information theory. Up to now, the analysis of sequential state discrimination has been confined to special cases. In this report, we consider a generalization of sequential state discrimination. Here, we do not limit the prior probabilities and the number of quantum states and receivers. We show that the generalized sequential state discrimination can be expressed as an optimization problem. Moreover, we investigate a structure of generalized sequential state discrimination for two quantum states and apply it to multiparty QKD. We demonstrate that when the number of receivers is not too many, generalized sequential state discrimination for two pure states can be suitable for multiparty QKD. In addition, we show that generalized sequential state discrimination for two mixed states can be performed with high optimal success probability. This optimal success probability is even higher than those of quantum reproducing and quantum broadcasting strategy. Thus, generalized sequential state discrimination of mixed states is adequate for performing multiparty QKD. Furthermore, we prove that generalized sequential state discrimination can be implemented experimentally by using linear optics. Finally, we analyze the security of multiparty QKD provided by optimal sequential state discrimination. Our analysis shows that the multiparty QKD guarantees nonzero secret key rate even in low channel efficiency.

## Introduction

In quantum mechanics, one cannot always discriminate quantum states that are non-orthogonal to each other. Therefore, a strategy to discriminate these quantum states is required. Investigation of the strategy for quantum state discrimination is one of the fundamental research fields in quantum information processing. The concept of quantum state discrimination can be understood as a game consisting of a sender Alice and a receiver Bob. In this game, Alice prepares a quantum state out of two or more than two quantum states, with a prior probability. It is assumed that Alice has informed Bob about the prior probabilities before Bob performs a measurement for quantum state discrimination. With the information, Bob measures Alice’s quantum state. Bob’s measurement outcome is divided into an inconclusive and a conclusive result. If the measurement outcome is conclusive, Bob can use the outcome to distinguish Alice’s quantum state. If it is inconclusive, Bob cannot figure out the quantum state that Alice had prepared. The purpose of quantum state discrimination is to maximize the probability that Bob’s conclusive result is correct. Many researchers have proposed a variety of discrimination strategies. In the minimum error discrimination strategy, Bob’s measurement is designed to obtain only a conclusive result^1–5^. The purpose of this strategy is to minimize the probability that a conclusive result is erroneous. In the unambiguous discrimination strategy, Bob’s measurement is designed to guarantee that his conclusive result is always correct^6–12^. The purpose of this strategy is to minimize the probability, that the outcome is inconclusive. In the maximal confidence strategy, Bob should maximize the confidence of a conclusive result^13^. Recently, other strategies, that interpolate between minimum error discrimination and unambiguous discrimination have been proposed. In the error margin strategy, Bob’s measurement is designed not to make his error probability surpass an error margin^14–17^. In the fixed rate of the inconclusive result strategy, the probability that Bob obtains an inconclusive result is fixed to a specific value^18–23^. It is well known that quantum state discrimination provides a variety of quantum information protocols. Especially, unambiguous discrimination can be fruitfully applied to quantum key distribution (QKD)^24^, quantum random number generator^25^ and quantum state tomography^26^.

In 2013, Bergou *et al*.^27^ proposed *the sequential state discrimination strategy*. This strategy can consist of many receivers (called Bob 1, Bob 2 … Bob N), who are separated and are not allowed to perform classical communication with each other. In sequential state discrimination, a sender Alice sends one out of two quantum states, with a prior probability to Bob 1. It is assumed that all receivers are aware of the prior probabilities, before they perform sequential state discrimination. Bob 1 performs a measurement to discriminate Alice’s quantum state. After the measurement, Bob 1 sends his post-measurement state to Bob 2. Then, Bob 2 performs his measurement to discriminate Bob 1’s post-measurement state. This process is sequentially performed. The purpose of sequential state discrimination is to maximize the probability that all receivers successfully discriminate Alice’s quantum state. According to Bergou *et al*.^27^, the optimal (maximum) success probability is non-zero, in general. It implies that Bob $I+1$ can obtain information about Alice’s quantum state, from Bob $I$’s post-measurement state. This result not only enables us to know the property of a non-projective measurement but also allows its application to multiparty QKD strategy. Bergou *et al*.^27^ and Pang *et al*.^28^ separately investigated the sequential state discrimination of two pure states with equal prior probabilities. Solis-Prosser *et al*.^29^ implemented the sequential state discrimination trategy. In their work, two polarized single photon states with equal prior probabilities, were considered. Moreover, Zhang *et al*.^30^ investigated the sequential state discrimination of two pure states with unequal prior probabilities. Hillery and Mimih^31^ considered the sequential state discrimination of $N$ symmetric pure state, with equal prior probabilities.

However, most studies of sequential state discrimination have been focused on special cases. In other words, the generalized structure of sequential state discrimination has not been investigated yet^32–34^. Therefore, in this report, we consider a generalization of sequential state discrimination. That is, in constructing sequential state discrimination, we do not limit the prior probabilities and the number of quantum states and receivers. Moreover, we consider the most general case of quantum states, in which every quantum state can be either pure^32^ or mixed state^33^. Because unambiguous discrimination of general mixed states is not known yet^35–37^, sequential state discrimination for general mixed states is beyond the scope of this paper. However, if mixed states are given in the form of Herzog’s work^36^, we can build generalized sequential state discrimination of two mixed states. Also, in terms of success probability, generalized sequential state discrimination provides better result in mixed states than in pure states.

First, we show that generalized sequential state discrimination can be expressed to a mathematical optimization problem. This optimization problem provides an optimal positive-operator-valued-measurement (POVM) condition, as well as an optimal success probability. Exploiting this structure, we explicitly investigate the generalized sequential state discrimination of two quantum states. Naturally, our investigation contains previous works^27,28^. Also, we apply it to multiparty QKD. We show that if the number of receivers is too many, generalized sequential state discrimination of two pure states can be performed, with very small optimal success probability. It means that generalized sequential state discrimination of two pure states can be suitable for multiparty QKD, only when the number of receivers is not too many. Meanwhile, generalized sequential state discrimination of two mixed states can be performed, with high optimal success probability. Especially, its optimal success probability exceeds those of quantum reproducing^27^ and quantum broadcasting^38,39^ strategy. It implies that generalized sequential state discrimination of two mixed states can be more suitable for multiparty QKD than other strategies.

In addition, we show that linear optics can be used to experimentally implement generalized sequential state discrimination. Here, our models can be implemented by modifying the Banaszek model^40^ or the Huttner model^41^. We show that generalized sequential state discrimination of binary coherent states^34^ can be implemented optimally. Moreover, we show that generalized sequential state discrimination of two mixed states can be implemented optimally. Further, we consider mixed states, which consists of coherent states. When an information carrier is a coherent state, which is robust in a noisy environment^42^, our model can be suitable for implementing a realistic multiparty QKD.

Finally, we analyze the security of multiparty QKD based on optimal sequential state discrimination. It is known that B92 protocol provides unconditional security^43^. Therefore, one can guess that the QKD based on generalized sequential state discrimination guarantees security. To show this, we evaluate the secret key rate^44^ of multiparty QKD based on generalized sequential state discrimination. Our result tells that the multiparty QKD guarantees nonzero secret key rate even in low channel efficiency. In addition, our multiparty QKD is composed of the method based on prepare and measure^24,45,46^ and is more robust in noise than the QKD of multipartite entanglement.

## Results

### Scenario of Sequential State Discrimination

**Figure 1:**
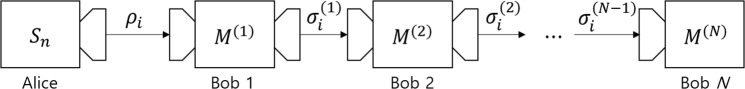
Schematic of the concept of sequential state discrimination. In this concept, Alice prepares ${\rho }_{i}\in {S}_{n}$, with a prior probability ${q}_{i}$. Bob 1 discriminates Alice’s quantum state, using POVM ${M}^{(1)}={\{{M}_{i}^{(1)}\}}_{i=0}^{n}$, without an error. Using POVM ${M}^{(2)}={\{{M}_{i}^{(2)}\}}_{i=0}^{n}$, Bob 2 also discriminates ${\sigma }_{i}^{\mathrm{(1)}}$ which is the post-measurement state of Bob 1, without an error. Then, this process is sequentially performed from Bob 3 to Bob $N$.

The concept of generalized sequential state discrimination can be understood as a game, consisting of a sender Alice and $N$ receivers such as Bob 1, Bob 2, $\ldots$, Bob $N$(see Fig. 1). In this scenario, every party acts as follows: Alice prepares a quantum state ${\rho }_{i}\in \{{\rho }_{1},\ldots ,{\rho }_{n}\}$, with a prior probability ${q}_{i}$ and sends ${\rho }_{i}$ to Bob 1. Bob 1 performs a POVM $\{{M}_{0}^{(1)},{M}_{1}^{(1)},\ldots ,{M}_{n}^{(1)}\}$ on Alice’s quantum state, for unambiguous discrimination. Here, ${M}_{j}^{\mathrm{(1)}}$ is a POVM element, corresponding to a measurement outcome $j$. If Bob 1 obtains a conclusive outcome ($j\ne 0$), he thinks Alice’s quantum state as ${\rho }_{j}$. If Bob 1 obtains an inconclusive result ($j=0$), he cannot figure out which quantum state Alice had prepared. If Bob 1 obtains a conclusive result, he sends a post-measurement state to Bob 2. Because in generalized sequential state discrimination every receiver should perform unambiguous discrimination, the post-measurement state of Bob 1 is given as ${\sigma }_{i}^{(1)}\propto {K}_{j}^{(1)}{\rho }_{i}{K}_{j}^{(1)\dagger }{\delta }_{ij}(i,j\ne 0)$. Here, ${\delta }_{ij}$ is the Kronecker delta and ${K}_{i}^{\mathrm{(1)}}$ is the Kraus operator, satisfying ${M}_{i}^{(1)}={K}_{i}^{(1)\dagger }{K}_{i}^{(1)}$. Likewise, Bob 2 performs unambiguous discrimination on Bob 1’s post-measurement state ${\sigma }_{i}^{\mathrm{(1)}}$, using POVM $\{{M}_{0}^{(2)},{M}_{1}^{(2)},\ldots ,{M}_{n}^{(2)}\}$. Then, Bob 2 sends his post-measurement state ${\sigma }_{i}^{(2)}\propto {K}_{j}^{(2)}{\sigma }_{i}^{(1)}{K}_{j}^{(2)\dagger }{\delta }_{ij}$ to Bob 3. This process is sequentially conducted from Bob 3 to Bob $N$. The average success probability of generalized sequential state discrimination is given as

1 $$\begin{array}{rcl}{P}_{s}^{({B}_{1},\ldots ,{B}_{N})} & = & \mathop{\sum }\limits_{i=1}^{n}\,{q}_{i}\text{Tr}[{\rho }_{i}{M}_{i}^{(1)}]\text{Tr}[{\rho }_{i}^{(1)}{M}_{i}^{(2)}]\text{Tr}[{\rho }_{i}^{(2)}{M}_{i}^{(3)}]\\  &  & \times \,\cdots \,\times \text{Tr}[{\rho }_{i}^{(N-1)}{M}_{i}^{(N)}].\end{array}$$

(In Eq. (1), each Bob 1, Bob 2, $\ldots$, Bob $N$ is briefly expressed as ${B}_{1},{B}_{2},\ldots ,{B}_{N}$). The purpose of generalized sequential state discrimination is to maximize the average success probability, as expressed in Eq. (1). In the process, every receiver should obey the following rules:

**Rule 1**. Bob 1, Bob 2, $\ldots$, Bob $N-1$ performs a nonoptimal unambiguous discrimination. However, Bob $N$ performs an optimal unambiguous discrimination.

**Rule 2**. Classical communication is forbidden between every receiver.

If Bob $I\in \{1,2,\ldots ,N-1\}$ performs an optimal unambiguous discrimination, Bob $I+1$ cannot obtain any information from Bob $I$’s post-measurement state. Moreover, if one of the receivers sends his measurement outcome through classical communication, an eavesdropper can steal the measurement outcome without being noticed by any receivers. Thus, it is reasonable that Rules 1 and 2 should be imposed on every receiver.

### Construction of the Optimization Problem

In this section, we express generalized sequential state discrimination as an optimization problem. To construct the optimization problem, we should involve not only two rules but also POVM conditions for every receiver. First, we should consider a POVM that performs an unambiguous discrimination.

#### POVM for unambiguous discrimination

Let us find the condition for the POVM $\{{M}_{0}^{\mbox{'}}{M}_{1},\ldots ,{M}_{n}\}$ that performs an unambiguous discrimination. This POVM should satisfy the following conditions (I) ${M}_{i}\ge 0(\forall i\in \{1,\ldots ,n\})$ (II) ${M}_{i}={M}_{i}^{\dagger }(\forall i\in \{1,\ldots ,n\})$ (III) ${M}_{0}+{M}_{1}+\cdots +{M}_{n}=I$ and (IV) $\text{Tr}{\rho }_{i}{M}_{j}={\delta }_{ij}\text{Tr}{\rho }_{i}{M}_{i}(\forall i,j\in \{1,\ldots ,n\})$. Here, the conditions of (I), (II), and (III) are positive-semidefinite, Hermitian and completeness condition, respectively. Especially, (IV) is the condition in which the POVM performs an unambiguous discrimination. However, understanding an unambiguous discrimination is confined only to a special set of quantum states ${S}_{n}=\{{\rho }_{1},{\rho }_{2},\ldots ,{\rho }_{n}\}$. If the set of quantum states ${S}_{n}$ satisfies the following theorem, there exists a POVM that performs an unambiguous discrimination on ${S}_{n}$.

##### Theorem 1

If^7^ $\text{supp}({\rho }_{i})$ satisfies $\text{supp}({\rho }_{i}) \nsubseteq {\cup }_{j\ne i}\,\text{supp}({\rho }_{k})$ for all ${\rho }_{i}\in {S}_{n}$, there exists a POVM that performs an unambiguous discrimination on ${S}_{n}$.

If ${S}_{n}$ satisfies Theorem 1, the support of ${M}_{i}$ can be spanned by the support of ${\rho }_{i}$, orthogonal to ${\cup }_{j\ne i}\,\text{supp}({\rho }_{k})$. For example, let us consider ${S}_{2}=\{{\rho }_{1},{\rho }_{2}\}$. Then, the support of ${M}_{1}({M}_{2})$ is spanned by the kernel of ${\rho }_{2}({\rho }_{1})$^7^. For ${\bar{S}}_{n}=\{{\psi }_{1},{\psi }_{2},\ldots ,{\psi }_{n}\}$, Theorem 1 is simply stated as follows.

##### Theorem 2

If^6^ ${\bar{S}}_{n}$ is a set of linearly independent pure states, there exists a POVM that performs an unambiguous discrimination on ${\bar{S}}_{n}$.

*Proof*. Suppose ${\bar{S}}_{n}$ is a set of linearly independent pure states. Then, Gram matrix $G={\{\langle {\psi }_{i}|{\psi }_{j}\rangle \}}_{i,j=1}^{n}$ is positive definite^47^. Thus, there exists an inverse of *G*. Using *G*^−1^, we can construct *M*_*i*_ as

2 $${M}_{i}={\alpha }_{i}|{\tilde{\psi }}_{i}\rangle \langle {\tilde{\psi }}_{i}|,\,|{\tilde{\psi }}_{i}\rangle =\mathop{\sum }\limits_{j=1}^{n}\,{G}_{ji}^{-1}|{\psi }_{j}\rangle .$$

Here, ${\alpha }_{i}\ge 0$. The inner product between $|{\psi }_{j}\rangle$ and $|{\tilde{\psi }}_{i}\rangle$ is simply calculated as

$$\langle {\psi }_{j}|{\tilde{\psi }}_{i}\rangle =\mathop{\sum }\limits_{j=1}^{n}\,{G}_{ki}^{-1}{G}_{jk}={(G{G}^{-1})}_{ji}={\delta }_{ji}.$$

Therefore, the equality $\langle {\psi }_{j}|{M}_{i}|{\psi }_{j}\rangle ={\delta }_{ij}\langle {\psi }_{i}|{M}_{i}|{\psi }_{i}\rangle$ holds for all POVM elements ${M}_{i}\in \{{M}_{1},\ldots ,{M}_{n}\}$. We notice that ${M}_{1},\ldots ,{M}_{n}$, in Eq. (2), are Hermitian and positive-semidefinite. According to the completeness condition, ${M}_{0}=I-{M}_{1}-\cdots -{M}_{n}$ is also Hermitian. Now, we show that ${M}_{0}$ can be positive-semidefinite. If ${\alpha }_{1},\ldots ,{\alpha }_{n}$ are efficiently small, ${M}_{0}$ tends to converge to $I$. In this case, ${M}_{0}$ is obviously positive-semidefinite, which completes the proof of Theorem. _□_

By Theorem 2, every POVM $\{{M}_{0},{M}_{1},\ldots ,{M}_{n}\}$ has a one-to-one correspondance with an $n$-dimensional real vector $({\alpha }_{1},{\alpha }_{2},\ldots ,{\alpha }_{n})$. Moreover, the POVM conditions can be expressed, in terms of every component in this real vector. The positive-semidefiniteness condition of ${M}_{0}$ can be obtained through the following theorem.

##### Theorem 3.

Let^32^ us define a Hermitian matrix $\bar{M}={\{\langle {\psi }_{i}|{M}_{0}|{\psi }_{j}\rangle \}}_{i,j=1}^{n}$ and all $m\times m(m < n)$ principal submatrices ${\bar{M}}_{m}$. ${M}_{0}$ is positive-semidefinite if and only if every $\bar{M}$ and $\forall {\bar{M}}_{m}$ is positive-semidefinite.

*Proof*. We exploit the fact that ${M}_{0}$ is positive-semidefinite if and only if $\langle \psi |{M}_{0}|\psi \rangle \ge 0$ for all $|\psi \rangle \in {\mathscr{H}}$^47^. Because ${\bar{S}}_{n}$ is a set of linearly independent pure states, every $|\psi \rangle \in {\mathscr{H}}$ can be expressed as $|\psi \rangle ={v}_{1}|{\psi }_{1}\rangle +\cdots +{v}_{n}|{\psi }_{n}\rangle$. We can easily obtain the following equality:

$$\langle \psi |{M}_{0}|\psi \rangle ={v}^{\dagger }\bar{M}v.$$

Here, $v=({v}_{1},{v}_{2},\ldots ,{v}_{n})\in {{\mathbb{C}}}^{n}$. In other words, $\langle \psi |{M}_{0}|\psi \rangle \ge 0$ for all $|\psi \rangle \in {\mathscr{H}}$, if and only if ${v}^{\dagger }\bar{M}v\ge 0$ for all $v\in {{\mathbb{C}}}^{n}$. Every components in $V$ needs not to be nonzero. That is, ${v}^{\dagger }\bar{M}v\ge 0$ if and only if $\forall \bar{M},{\bar{M}}_{m}$ are positive-semidefinite. □

According to Rule 2, no receiver can perform any classical communication. In sequential state discrimination, the post-measurement state contains a measurement outcome. Hence, we should construct a Kraus operator, corresponding to POVM^48^. According to Eq. (2), every POVM, corresponding to conclusive result, is rank-1. Therefore, ${K}_{i}$ is expressed as ${K}_{i}={U}_{i}\sqrt{{M}_{i}}$, from singular value decomposition. Here, ${U}_{i}$ is unitary operator and $\sqrt{{M}_{i}}$ is a square-root operator of ${M}_{i}$. Then, the Kraus operator, corresponding to conclusive result, is constructed as

$${K}_{i}={U}_{i}\sqrt{{M}_{i}}={U}_{i}\sqrt{{\alpha }_{i}}|{\tilde{\psi }}_{i}\rangle \langle {\tilde{\psi }}_{i}|=\sqrt{{\alpha }_{i}}({U}_{i}|{\tilde{\psi }}_{i}\rangle )\langle {\tilde{\psi }}_{i}|=\sqrt{{\alpha }_{i}}|{\phi }_{i}\rangle \langle {\tilde{\psi }}_{i}|.$$

Then, the post-measurement state, corresponding to conclusive result $i$, is expressed as $|{\phi }_{i}\rangle \propto {K}_{i}|{\psi }_{i}\rangle$^48^. Now, we construct the Kraus operator ${K}_{0}$, which satisfies ${M}_{0}={K}_{0}^{\dagger }{K}_{0}$. It is complicated to obtain ${K}_{0}$ from ${M}_{0}$. If we assume that every pure state in ${\bar{S}}_{n}$ spans ${\mathscr{H}}$, when ${K}_{0}|{\psi }_{i}\rangle$, for some $i$, is not involved in $\{{K}_{j}|{\psi }_{j}\rangle {\}}_{j=1}^{n}$, every post-meaurement state $\{{K}_{0}|{\psi }_{i}\rangle {\}}_{i=1}^{n}\cup \{{K}_{i}|{\psi }_{i}\rangle {\}}_{i=1}^{n}$ is linearly dependent. Therefore, post-measurement states cannot be discriminated unambiguously. This implies that every post-measurement state ${K}_{0}|{\psi }_{i}\rangle$ should be involved in $\{{K}_{i}|{\psi }_{i}\rangle {\}}_{i=1}^{n}$. We construct ${K}_{0}$ that maps $|{\psi }_{i}\rangle$ into ${K}_{i}|{\psi }_{i}\rangle$, and it is expressed as^32,33^

$${K}_{0}=\sqrt{{\gamma }_{1}}|{\phi }_{1}\rangle \langle {\tilde{\psi }}_{1}|+\sqrt{{\gamma }_{2}}|{\phi }_{2}\rangle \langle {\tilde{\psi }}_{2}|+\cdots +\sqrt{{\gamma }_{n}}|{\phi }_{n}\rangle \langle {\tilde{\psi }}_{n}|.$$

Here, ${\gamma }_{i}\ge 0$. From ${\gamma }_{i}=1-{\alpha }_{i}$, we can see that ${\alpha }_{i}$ is less than 1. We should find ${\gamma }_{i}$ to satisfy ${M}_{0}={K}_{0}^{\dagger }{K}_{0}$. To solve this problem, we exploit the following theorem.

##### Theorem 4.

Let^33^ ${\bar{S}}_{n}$ be a set of linearly independent pure states. Then, $A=B$ if and only if $\langle {\psi }_{i}|A|{\psi }_{j}\rangle =\langle {\psi }_{i}|B|{\psi }_{j}\rangle (\forall i,j)$.

Substituting both $A$ and $B$, in Theorem 4, into each ${M}_{0}$ and ${K}_{0}^{\dagger }{K}_{0}$, we obtain

3 $${\gamma }_{i}=1-{\alpha }_{i},\,\sqrt{{\gamma }_{i}{\gamma }_{j}}\langle {\phi }_{i}|{\phi }_{j}\rangle =\langle {\psi }_{i}|{\psi }_{j}\rangle .$$

Equation (3) includes an argument from Bergou *et al*.^27^ Combining these two equalities, we derive an overlap between two post-measurement states as

4 $$\langle {\phi }_{i}|{\phi }_{j}\rangle =\frac{\langle {\psi }_{i}|{\psi }_{j}\rangle }{\sqrt{(1-{\alpha }_{i})(1-{\alpha }_{j})}}.$$

According to Eq. (4), the overlap $\langle {\phi }_{i}|{\phi }_{j}\rangle$ is larger than or equal to $\langle {\psi }_{i}|{\psi }_{j}\rangle$. Because $|\langle {\phi }_{i}|{\phi }_{j}\rangle |\le 1$, we obtain

5 $$(1-{\alpha }_{i})(1-{\alpha }_{j})\ge |\langle {\psi }_{i}|{\psi }_{j}\rangle {|}^{2},\,\forall i,j.$$

This equality corresponds to the POVM condition, which performs an unambiguous discrimination on a pure state (${\bar{S}}_{2}$). If every receiver performs an optimal unambiguous discrimination, Eq. (5) becomes a strict equality. Then, the overlap between post-measurement states becomes one, according to Eq. (4). Hence, to obey Rule 1, the POVM of Bob 1, Bob 2, $\ldots$, Bob $N-1$ should not satisfy the equality of Eq. (5). Furthermore, because every submatrix ${\bar{M}}_{ab}$ should be positive-semidefinite, Eq. (5) is also involved in the POVM condition, performing an unambiguous discrimination of $n$ pure states.

#### Generalizing POVM for mixed state discrimination

In this section, we investigate the generalized sequential state discrimination of mixed quantum states. Unfortunately, an explicit form of POVM, that performs an unambiguous discrimination of arbitrary mixed states is unknown. That is because we do not know how to deal with Theorem 1. When mixed states can be expressed in the form given by Herzog’s work^36^, POVM can be constructed explicitly. Suppose that every mixed state has the same rank. Then, each mixed state on Hilbert space ${\mathscr{H}}={{\mathscr{H}}}_{1}\oplus {{\mathscr{H}}}_{2}\oplus \cdots \oplus {{\mathscr{H}}}_{m}$ has the following form:

6 $${\rho }_{i}={r}_{i1}|{r}_{i1}\rangle \langle {r}_{i1}|\oplus {r}_{i2}|{r}_{i2}\rangle \langle {r}_{i2}|\oplus \cdots \oplus {r}_{im}|{r}_{im}\rangle \langle {r}_{im}|,\,{r}_{i1},{r}_{i2},\ldots ,{r}_{im} > 0.$$

Here, $m=\text{rank}({\rho }_{i})$. According to the trace condition of ${\rho }_{i}$, ${\sum }_{j=1}^{m}\,{r}_{ij}=1$ holds for all $i$. If we consider that every mixed state has a form like that of Eq. (6), then Theorem 1 can be explicitly expressed as follows:

##### Theorem 5.

Suppose^33^ that all elements in *S*_*n*_ have the same form as that of Eq. (6). If $\{|{r}_{1j}\rangle ,|{r}_{2j}\rangle ,\ldots ,|{r}_{nj}\rangle \}$ are linearly independent for every $j\in \{1,\ldots ,m\}$, then there exists a POVM that performs an unambiguous discrimination on *S*_*n*_.

*Proof*. Let us construct the POVM element *M*_*i*_, corresponding to measurement outcome *i* as

$${M}_{i}={M}_{i1}\oplus {M}_{i2}\oplus \cdots \oplus {M}_{im}.$$

Here, $\{{M}_{0j},{M}_{1j},\ldots ,{M}_{nj}\}$ is a sub-POVM, defined on sub-Hilbert space ${{\mathscr{H}}}_{j}$. Because every ${M}_{ij}$ is positive-semidefinite, ${M}_{i}$ is also positive-semidefinite. The completeness condition of POVM $\{{M}_{0},{M}_{1},\ldots ,{M}_{n}\}$ is straightforwardly proved as

$$\begin{array}{rcl}\mathop{\sum }\limits_{i=0}^{n}\,{M}_{i} & = & \mathop{\sum }\limits_{i=0}^{n}\,{M}_{i1}\oplus {M}_{i2}\oplus \cdots \oplus {M}_{im}\\  & = & (\mathop{\sum }\limits_{i=0}^{n}\,{M}_{i1})\oplus (\mathop{\sum }\limits_{i=0}^{n}\,{M}_{i2})\oplus \cdots \oplus (\mathop{\sum }\limits_{i=0}^{n}\,{M}_{im})\\  & = & {I}_{1}\oplus {I}_{2}\oplus \cdots \oplus {I}_{m}\\  & = & I.\end{array}$$

Here, ${I}_{j}$ is an identity operator, defined on sub-Hilbert space ${{\mathscr{H}}}_{j}$. Sub-POVM $\{{M}_{0j},{M}_{1j},\ldots ,{M}_{nj}\}$ only acts on $\{|{r}_{1j}\rangle ,|{r}_{2j}\rangle ,\ldots ,|{r}_{nj}\rangle \}$. If $\{|{r}_{1j}\rangle ,|{r}_{2j}\rangle ,\ldots ,|{r}_{nj}\rangle \}$ are linearly independent, every sub-POVM is obtained, using similar process as Theorems 2 and 3. Therefore, we obtain POVM that performs unambiguous discrimination, which completes the proof of Theorem. □

With the help of Theorem 5, we can apply a method that deals with the discrimination of pure states into a mixed-state case. If $i\ne 0$, the POVM element ${M}_{i}$ can be expressed as

7 $${M}_{i}={\alpha }_{i1}|{\tilde{r}}_{i1}\rangle \langle {\tilde{r}}_{i1}|\oplus {\alpha }_{i2}|{\tilde{r}}_{i2}\rangle \langle {\tilde{r}}_{i2}|\oplus \cdots \oplus {\alpha }_{im}|{\tilde{r}}_{im}\rangle \langle {\tilde{r}}_{im}|.$$

According to the completeness condition, ${M}_{0}$ is given as

8 $$\begin{array}{rcl}{M}_{0} & = & {M}_{01}\oplus {M}_{02}\oplus \cdots \oplus {M}_{0m}\\  & = & ({I}_{1}-\mathop{\sum }\limits_{i=0}^{n}\,{M}_{01})\oplus ({I}_{2}-\mathop{\sum }\limits_{i=0}^{n}\,{M}_{02})\oplus \cdots \oplus ({I}_{{m}_{0}}-\mathop{\sum }\limits_{i=0}^{n}\,{M}_{0{m}_{0}})\\  & = & ({I}_{1}-\mathop{\sum }\limits_{i=0}^{n}\,{\alpha }_{01}{\tilde{r}}_{01}{\tilde{r}}_{01})\oplus \cdots \oplus ({I}_{{m}_{0}}-\mathop{\sum }\limits_{i=0}^{n}\,{\alpha }_{0m}{\tilde{r}}_{0m}{\tilde{r}}_{0m}).\end{array}$$

Then, the Kraus operator ${K}_{i}$, corresponding to ${M}_{i}$ of Eq. (8), is given as

$${K}_{i}=\sqrt{{\alpha }_{i1}}|{s}_{i1}\rangle \langle {\tilde{r}}_{i1}|\oplus \sqrt{{\alpha }_{i2}}|{s}_{i2}\rangle \langle {\tilde{r}}_{i2}|\oplus \cdots \oplus \sqrt{{\alpha }_{im}}|{s}_{im}\rangle \langle {\tilde{r}}_{im}|.$$

Hence, the post-measurement state ${\sigma }_{i}$ is expressed as

$$\begin{array}{rcl}{\sigma }_{i} & = & \frac{{K}_{i}{\rho }_{i}{K}_{i}^{\dagger }}{Tr[{K}_{i}{\rho }_{i}{K}_{i}^{\dagger }]}\\  & = & \frac{{r}_{i1}{\alpha }_{i1}|{s}_{i1}\rangle \langle {s}_{i1}|\oplus {r}_{i2}{\alpha }_{i2}|{s}_{i2}\rangle \langle {s}_{i2}|\oplus \cdots \oplus {r}_{im}{\alpha }_{im}|{s}_{im}\rangle \langle {s}_{im}|}{{r}_{i1}{\alpha }_{i1}+{r}_{i2}{\alpha }_{i2}+\cdots +{r}_{im}{\alpha }_{im}}.\end{array}$$

We can obtain the Kraus operator ${K}_{0}$, corresponding to ${M}_{0}$, by exploiting Theorem 3. Because every eigenvector of ${\sigma }_{i}$ should satisfy

9 $$\langle {s}_{ij}|{s}_{kj}\rangle =\frac{\langle {r}_{ij}|{r}_{kj}\rangle }{\sqrt{(1-{\alpha }_{ij})(1-{\alpha }_{kj})}},$$

from Eq. (9) we can obtain

10 $$(1-{\alpha }_{ij})(1-{\alpha }_{kj})\ge |{r}_{ij}|{r}_{kj}{|}^{2}.$$

Both Eqs. (9) and (10) imply the following meaning. if either ${\alpha }_{ij}$ or ${\alpha }_{kj}$ is nonzero, $|\langle {s}_{ij}|{s}_{kj}\rangle |$ is larger than $|\langle {r}_{ij}|{r}_{kj}\rangle |$. That is, the support of two post-measurement states is more overlapped than that of Alice’s mixed states. If an optimal unambiguous discrimination is performed, Eq. (10) becomes a strict equality. Moreover, according to Eq. (9), $|\langle {s}_{ij}|{s}_{kj}\rangle |$ becomes equal to 1. Therefore, for all $i$, $\text{supp}({\sigma }_{i})={\cup }_{j\ne i}\,\text{supp}({\sigma }_{j})$ holds, which implies that $\{{\sigma }_{1},\ldots ,{\sigma }_{n}\}$ cannot be discriminated, without any error.

Now, let us consider the case where every ${\rho }_{i}$ has a different rank. Without loss of generality, we can assume an inequality such as $\text{rank}({\rho }_{1}) > \text{rank}({\rho }_{2}) > \cdots  > \text{rank}({\rho }_{n})$. Then, a POVM element can be constructed as

$${M}_{i}={M}_{i1}\oplus {M}_{i2}\oplus \cdots \oplus {M}_{i{m}_{i}},$$

where ${m}_{i}=\text{rank}({\rho }_{i})$. Each POVM element can be constructed in the following manner: If $1\le j\le {m}_{1}$, sub-POVM $\{{M}_{0j},{M}_{1j},\ldots ,{M}_{nj}\}$ discriminates $\{|{r}_{1j}\rangle ,|{r}_{2j}\rangle ,\ldots ,|{r}_{nj}\rangle \}$ without any error. If ${m}_{1} < j\le {m}_{2}$, sub-POVM $\{{M}_{0j},{M}_{1j},\ldots ,{M}_{nj}\}$ discriminates $\{|{r}_{1j}\rangle ,|{r}_{2j}\rangle ,\ldots ,|{r}_{n-1j}\rangle \}$. The remaining part of the POVM elements can also be constructed inductively.

#### Optimization problem for pure states case

Assume that $|{\psi }_{i}\rangle \in {\bar{S}}_{n}$ is prepared, with a prior probability ${q}_{i}$. Then, the POVM $\{{M}_{0}^{(I)},{M}_{1}^{(I)},\ldots ,{M}_{n}^{(I)}\}$ of Bob $I\in \{1,\ldots ,N\}$ corresponds to a vector $({\alpha }_{1}^{(I)},\ldots ,{\alpha }_{n}^{(I)})$ in an *n*–dimensional real vector space. Using this vector, we can express Eq. (1) as

11 $${P}_{s}^{({B}_{1},\ldots ,{B}_{N})}=\mathop{\sum }\limits_{i=1}^{n}\,{q}_{i}{\alpha }_{i}^{(1)}{\alpha }_{i}^{(2)}{\alpha }_{i}^{(3)}\times \cdots \times {\alpha }_{i}^{(N)},$$

where ${\alpha }_{i}^{(1)}=\langle {\psi }_{i}|{M}_{i}^{(1)}|{\psi }_{i}\rangle$, ${\alpha }_{i}^{(I)}=\langle {\phi }_{i}^{(I-1)}|{M}_{i}^{(I)}|{\phi }_{i}^{(I-1)}\rangle$. The constraints on POVM can be expressed in terms of $({\alpha }_{1}^{(I)},\ldots ,{\alpha }_{n}^{(I)})$. Applying Rule 1 and Rule 2^32^, we can construct POVM conditions as

12 $$\begin{array}{lll}({\alpha }_{1}^{(I)},\ldots ,{\alpha }_{n}^{(I)}) & \in  & {C}_{\text{int}}^{(I)},\,I\le N-1\\ ({\alpha }_{1}^{(N)},\ldots ,{\alpha }_{n}^{(N)}) & \in  & \partial {C}^{(N)}.\end{array}$$

Here, ${C}_{\text{int}}^{(I)}$ and $\partial {C}^{(I)}$ are defined as

$$\begin{array}{rcl}{C}^{(I)} & = & \{({\alpha }_{1}^{(I)},\ldots ,{\alpha }_{n}^{(I)})\in {{\mathbb{R}}}^{n}|\bar{M}\ge 0\wedge {\bar{M}}_{m}\ge 0\,\forall m < n\},\\ {C}_{\text{int}}^{(I)} & = & \{({\alpha }_{1}^{(I)},\ldots ,{\alpha }_{n}^{(I)})\in {{\mathbb{R}}}^{n}|\bar{M} > 0\wedge {\bar{M}}_{m}\ge 0\,\forall m < n\},\\ \partial {C}^{(I)} & = & \{({\alpha }_{1}^{(I)},\ldots ,{\alpha }_{n}^{(I)})\in {{\mathbb{R}}}^{n}|\bar{M}=0\wedge {\bar{M}}_{m}\ge 0\,\forall m < n\}.\end{array}$$

Then, Bob $I$’s POVM condition is expressed as ${C}^{(I)}={C}_{\text{int}}^{(I)}\cup \partial {C}^{(I)}$. Combining Eq. (11) with Eq. (12), we can express generalized sequential state discrimination as following optimization problem^32^:

13 $$\begin{array}{ll}\text{maximize} & {P}_{s}^{({B}_{1},\ldots ,{B}_{N})}=\mathop{\sum }\limits_{i=1}^{n}\,{q}_{i}{\alpha }_{i}^{(1)}{\alpha }_{i}^{(2)}{\alpha }_{i}^{(3)}\times \cdots \times {\alpha }_{i}^{(N)}\\ \text{subject}\,\text{to} & ({\alpha }_{1}^{(I)},\ldots ,{\alpha }_{n}^{(I)})\in {C}_{\text{int}}^{(I)},\,\forall I\le N-1\\  & ({\alpha }_{1}^{(N)},\ldots ,{\alpha }_{n}^{(N)})\in \partial {C}^{(N)}.\end{array}$$

Now, let us investigate the geometric properties of each ${C}^{(I)}$. The set of POVM that performs an unambiguous discrimination is convex and ${C}^{(I)}$ is also convex:

##### Theorem 6.

Every^32^ *C*^(*I*)^ is convex.

It is important to investigate the relation between ${C}^{(I)}$ and ${C}^{(I+\mathrm{1)}}$, to analyze the generalized sequential state discrimination. In our previous work^32^, we proposed the following conjecture:

##### Conjecture 1.

If^32^ there exists nonzero ${\alpha }_{i}^{(I)}$ in $({\alpha }_{1}^{(I)},\ldots ,{\alpha }_{n}^{(I)})$, the size of set ${C}^{(I+1)}$ is smaller than that of ${C}^{(I)}$.

Considering the discrimination problem of two pure states, we can confirm that Conjecture 1 holds. Conjecture 1 has the following meaning. When the real vector $({\alpha }_{1}^{(I)},\ldots ,{\alpha }_{n}^{(I)})$ has at least one nonzero component ${\alpha }_{1}^{(I)}$, Bob $I$ can obtain partial information of Alice’s quantum state, by performing a measurement on Bob $I-1$’s post-measurement state, with a nonzero probability. Conjecture 1 implies that options for POVM that Bob $I+1$ can choose are limited, as Bob $I$ obtains the information. In the extreme case, if Bob $I$ obtains the maximal information, then Bob $I+1$ cannot construct a POVM to unambiguously discriminate Bob $I$’s post-measurement state, which means that Bob $I+1$ cannot obtain any information from Bob $I$’s post-measurement state. Hence, we can propose the following conjecture:

##### Conjecture 2.

If^32^ $({\alpha }_{1}^{(I)},\ldots ,{\alpha }_{n}^{(I)})\in \partial {C}^{(I)}$, $({\alpha }_{1}^{(I+1)},\ldots ,{\alpha }_{n}^{(I+1)})=(0,\ldots ,0)$ is the only element of ${C}^{(I+\mathrm{1)}}$.

If Alice prepares a pure state from ${\bar{S}}_{2}$, both Conjecture 1 and Conjecture 2 hold^32^. In the case of $N=3$, we can numerically check that both conjectures 1 and 2 are correct.

#### Optimization problem for the mixed states case

Now, let us consider a mixed states case. When Alice prepares ${\rho }_{i}$, which is expressed as Eq. (6), Bob $I\in \{1,\ldots ,N\}$’s POVM $\{{M}_{0}^{(I)},{M}_{1}^{(I)},\ldots ,{M}_{n}^{(I)}\}$ corresponds to the real vector $({\alpha }_{11},\ldots ,{\alpha }_{nm})\in {{\mathbb{R}}}^{nm}$. This real vector is included in ${\tilde{C}}^{(I)}={\tilde{C}}_{\text{int}}^{(I)}\cup \partial {\tilde{C}}^{(I)}$, where ${\tilde{C}}_{\text{int}}^{(I)}$ and $\partial {\tilde{C}}^{(I)}$ are respectively defined as

$$\begin{array}{rcl}{\tilde{C}}_{\text{int}}^{(I)} & = & {\tilde{C}}_{\text{int},1}^{(I)}\cap {\tilde{C}}_{\text{int},2}^{(I)}\cap \cdots \cap {\tilde{C}}_{\text{int},m}^{(I)},\\ \partial {\tilde{C}}^{(I)} & = & \partial {\tilde{C}}_{1}^{(I)}\cap \partial {\tilde{C}}_{2}^{(I)}\cap \cdots \cap \partial {\tilde{C}}_{m}^{(I)}.\end{array}$$

Here, ${\tilde{C}}_{\text{int},j}^{(I)}$ and $\partial {\tilde{C}}^{(I)}$ are respectively defined as

$$\begin{array}{rcl}{\tilde{C}}_{\text{int},j}^{(I)} & = & \{({\alpha }_{1j},\ldots ,{\alpha }_{nj})\in {{\mathbb{R}}}^{n}|{\bar{M}}_{j} > 0\wedge {\bar{M}}_{j,m}\ge 0\,\forall m < n\},\\ \partial {\tilde{C}}_{j}^{(I)} & = & \{({\alpha }_{1j},\ldots ,{\alpha }_{nj})\in {{\mathbb{R}}}^{n}|{\bar{M}}_{j}=0\wedge {\bar{M}}_{j,m}\ge 0\,\forall m < n\}.\end{array}$$

${\bar{M}}_{j}={\{\langle {r}_{ij}|{M}_{0j}|{r}_{kj}\rangle \}}_{i,k=1}^{n}$ and ${\bar{M}}_{j,m}$ are $m\times m$ principal submatrices. Then, we can express generalized sequential state discrimination of mixed states, as following optimization problem^33^:

$$\begin{array}{ll}\text{maximize} & {P}_{s}^{({B}_{1},\ldots ,{B}_{N})}=\mathop{\sum }\limits_{i=1}^{n}\,{q}_{i}({r}_{i1}\,\mathop{\prod }\limits_{I=1}^{N}\,{\alpha }_{i1}^{(I)}+{r}_{i2}\,\mathop{\prod }\limits_{I=1}^{N}\,{\alpha }_{i2}^{(I)}+\cdots {r}_{im}\,\mathop{\prod }\limits_{I=1}^{N}\,{\alpha }_{im}^{(I)})\\ \text{subject}\,\text{to} & ({\alpha }_{1j}^{(I)},\ldots ,{\alpha }_{nj}^{(I)})\in {\tilde{C}}_{\text{int},j}^{(I)},\,\forall j,\,\forall I\le N-1\\  & ({\alpha }_{1j}^{(N)},\ldots ,{\alpha }_{nj}^{(N)})\in \partial {\tilde{C}}_{j}^{(N)}.\,\forall j\end{array}$$

When $m=1$, this optimization problem describes the generalized sequential state discrimination of pure states. Note that the *j*-th constraint only affects sub-POVM ${\sum }_{i=1}^{n}\,{q}_{i}\,{\prod }_{I=1}^{N}\,{\alpha }_{ij}^{(I)}$. From this property, this optimization problem can be partitioned into the following sub-optimization problems^33^:

$$\begin{array}{ll}\text{maximize} & {p}_{s,j}=\mathop{\sum }\limits_{i=1}^{n}\,{q}_{i}{r}_{ij}(\mathop{\prod }\limits_{I=1}^{N}\,{\alpha }_{ij}^{(I)})\\ \text{subject}\,\text{to} & ({\alpha }_{1j}^{(I)},\ldots ,{\alpha }_{nj}^{(I)})\in {\tilde{C}}_{\text{int},j}^{(I)},\,\forall I\le N-1\\  & ({\alpha }_{1j}^{(N)},\ldots ,{\alpha }_{nj}^{(N)})\in \partial {\tilde{C}}_{j}^{(N)}.\end{array}$$

Then, the optimal success probability is expressed as $\text{max}\,{P}_{s}^{({B}_{1},\ldots ,{B}_{N})}=\text{max}\,{p}_{s,1}+\text{max}\,{p}_{s,2}+\cdots +\text{max}\,{p}_{s,m}$.

Next, we consider the case that no mixed state has the same rank. More precisely, we assume that $\text{rank}({\rho }_{1}) > \text{rank}({\rho }_{1}) > \cdots  > \text{rank}({\rho }_{n})$, without loss of generality. If $\text{rank}({\rho }_{l}) < j\le \text{rank}({\rho }_{l+1})$, the *j*-th sub-optimization problem is given as

$$\begin{array}{ll}\text{maximize} & {p}_{s,j}=\mathop{\sum }\limits_{i=1}^{n-l}\,{q}_{i}{r}_{ij}(\mathop{\prod }\limits_{I=1}^{N}\,{\alpha }_{ij}^{(I)})\\ \text{subject}\,\text{to} & ({\alpha }_{1j}^{(I)},\ldots ,{\alpha }_{nj}^{(I)})\in {\tilde{C}}_{\text{int},j}^{(I)},\,\forall I\le N-1\\  & ({\alpha }_{1j}^{(N)},\ldots ,{\alpha }_{nj}^{(N)})\in \partial {\tilde{C}}_{j}^{(N)}.\end{array}$$

Because every sub-optimization problem in the case of mixed states is the same as that in the pure state case, if every mixed state is expressed as Eq. (6), we can apply the method for pure states to the generalized sequential state discrimination of mixed states. Unfortunately, Eq. (6) is not the case of the most general mixed state. However, if we use these mixed states as an information carrier, the optimal success probability of the generalized sequential state discrimination can exceed that of the quantum reproducing^27^ and the quantum broadcasting strategy^39^. This implies that generalized sequential state discrimination is a more suitable strategy for application to multiparty QKD than the other two strategies. Furthermore, Eq. (6) can be implemented using linear optics. In the next sections, we explain these advantages in detail.

### Generalized Sequential State Discrimination of Two Quantum States

In this section, we consider the optimization problem proposed in the previous section. We deal not only with the problem of two pure states but also with the problem of two mixed states in a multi-receiver case.

#### Generalized sequential state discrimination of two pure states

Here, we consider the generalized sequential state discrimination with an arbitrary $N$. First, let us consider the case of $N=3$. The three receivers are denoted as Bob, Charlie, and David, and each POVM of Bob, Charlie, and David corresponds to the two dimensional real vectors $({\alpha }_{1},{\alpha }_{2})$, $({\beta }_{1},{\beta }_{2})$, and $({\gamma }_{1},{\gamma }_{2})$, respectively. According to Eq. (12), each real vector should satisfy

14 $$({\alpha }_{1},{\alpha }_{2})\in {C}_{\text{int}}^{(\text{B})},\,({\beta }_{1},{\beta }_{2})\in {C}_{\text{int}}^{(\text{C})},\,({\gamma }_{1},{\gamma }_{2})\in \partial {C}^{(\text{D})}.$$

where ${C}_{\text{int}}^{(X)}$ and $\partial {C}^{(X)}(X\in \{B,C,D\})$ are respectively defined as^32^

$$\begin{array}{rcl}{C}_{\text{int}}^{(\text{B})} & = & \{({\alpha }_{1},{\alpha }_{2})\in {{\mathbb{R}}}^{2}|(1-{\alpha }_{1})(1-{\alpha }_{2}) > |\langle {\psi }_{1}|{\psi }_{2}\rangle {|}^{2}\},\\ {C}_{\text{int}}^{(\text{C})} & = & \{({\beta }_{1},{\beta }_{2})\in {{\mathbb{R}}}^{2}|(1-{\beta }_{1})(1-{\beta }_{2}) > |\langle {\phi }_{1}^{(\text{B})}|{\phi }_{2}^{(\text{B})}\rangle {|}^{2}\},\\ {C}_{\text{int}}^{(\text{D})} & = & \{({\gamma }_{1},{\gamma }_{2})\in {{\mathbb{R}}}^{2}|(1-{\gamma }_{1})(1-{\gamma }_{2}) > |\langle {\phi }_{1}^{(\text{C})}|{\phi }_{2}^{(\text{C})}\rangle {|}^{2}\},\\ \partial {C}^{(\text{B})} & = & \{({\alpha }_{1},{\alpha }_{2})\in {{\mathbb{R}}}^{2}|(1-{\alpha }_{1})(1-{\alpha }_{2})=|\langle {\psi }_{1}|{\psi }_{2}\rangle {|}^{2}\},\\ \partial {C}^{(\text{C})} & = & \{({\beta }_{1},{\beta }_{2})\in {{\mathbb{R}}}^{2}|(1-{\beta }_{1})(1-{\beta }_{2})=|\langle {\phi }_{1}^{(\text{B})}|{\phi }_{2}^{(\text{B})}\rangle {|}^{2}\},\\ \partial {C}^{(\text{D})} & = & \{({\gamma }_{1},{\gamma }_{2})\in {{\mathbb{R}}}^{2}|(1-{\gamma }_{1})(1-{\gamma }_{2})=|\langle {\phi }_{1}^{(\text{C})}|{\phi }_{2}^{(\text{C})}\rangle {|}^{2}\}.\end{array}$$

The set of POVM, labeled as $\text{X}\in \{\text{B},\text{C},\text{D}\}$, can be expressed as ${C}^{(\text{X})}={C}_{\text{int}}^{(\text{X})}\cup \partial {C}^{(\text{X})}$. Therefore, Eq. (13) becomes^32^

15 $$\begin{array}{ll}\text{maximize} & {P}_{s}^{(\text{B},\text{C},\text{D})}={q}_{1}{\alpha }_{1}{\beta }_{1}{\gamma }_{1}+{q}_{2}{\alpha }_{2}{\beta }_{2}{\gamma }_{2}\\ \text{subject}\,\text{to} & (1-{\alpha }_{1})(1-{\alpha }_{2}) > |\langle {\psi }_{1}|{\psi }_{2}\rangle {|}^{2}\\  & (1-{\beta }_{1})(1-{\beta }_{2}) > |\langle {\phi }_{1}^{(\text{B})}|{\phi }_{2}^{(\text{B})}\rangle {|}^{2}\\  & (1-{\gamma }_{1})(1-{\gamma }_{2})=|\langle {\phi }_{1}^{(\text{C})}|{\phi }_{2}^{(\text{C})}\rangle {|}^{2}\end{array}$$

To solve this problem, we need to consider the equality constraint of Eq. (15). David’s optimal condition of generalized sequential state discrimination can be obtained by finding a tangential point $({\gamma }_{1},{\gamma }_{2})$ between a plane ${P}_{s}^{(\text{B},\text{C},\text{D})}=({q}_{1}{\alpha }_{1}{\beta }_{1}){\gamma }_{1}+({q}_{2}{\alpha }_{2}{\beta }_{2}){\gamma }_{2}$ and a surface $(1-{\gamma }_{1})(1-{\gamma }_{2})=|\langle {\phi }_{1}^{(C)}|{\phi }_{2}^{(C)}\rangle {|}^{2}$. When this tangential point is substituted into Eq. (15), Eq. (15) becomes the following optimization problem:

16 $$\begin{array}{ll}\text{maximize} & {P}_{s}^{(\text{B},\text{C},\text{D})}={q}_{1}{\alpha }_{1}{\beta }_{1}+{q}_{2}{\alpha }_{2}{\beta }_{2}-2|\langle {\psi }_{1}|{\psi }_{2}\rangle |\sqrt{\frac{{q}_{1}{q}_{2}{\alpha }_{1}{\alpha }_{2}{\beta }_{1}{\beta }_{2}}{(1-{\alpha }_{1})(1-{\alpha }_{2})(1-{\beta }_{1})(1-{\beta }_{2})}}\\ \text{subject}\,\text{to} & (1-{\alpha }_{1})(1-{\alpha }_{2}) > |\langle {\psi }_{1}|{\psi }_{2}\rangle {|}^{2}\\  & {\beta }_{2}\le \frac{{\beta }_{1}(1-{\beta }_{1})}{{\beta }_{1}(1-{\beta }_{1})+{\mathscr{X}}({\alpha }_{1},{\alpha }_{2})},\,{\mathscr{X}}({\alpha }_{1},{\alpha }_{2})=\frac{{q}_{2}{\alpha }_{2}}{{q}_{1}{\alpha }_{1}}\frac{|\langle {\psi }_{1}|{\psi }_{2}\rangle {|}^{2}}{(1-{\alpha }_{1})(1-{\alpha }_{2})},\\  & {\beta }_{1}\le \frac{{\beta }_{2}(1-{\beta }_{2})}{{\beta }_{2}(1-{\beta }_{2})+{\mathscr{Y}}({\alpha }_{1},{\alpha }_{2})},\,{\mathscr{Y}}({\alpha }_{1},{\alpha }_{2})=\frac{{q}_{1}{\alpha }_{1}}{{q}_{2}{\alpha }_{2}}\frac{|\langle {\psi }_{1}|{\psi }_{2}\rangle {|}^{2}}{(1-{\alpha }_{1})(1-{\alpha }_{2})}.\end{array}$$

The detailed derivation of Eq. (16) can be found in the Methods section. Because this optimization problem is difficult to solve analytically, one may apply a numerical method to solve it. Therefore, for the numerical method, a penalty function may be used to solve this constrained optimization problem^49^.

To search for the optimal condition of $({\alpha }_{1},{\alpha }_{2},{\beta }_{1},{\beta }_{2})$, we need to find the condition where the derivative $\partial {P}_{s}^{(\text{B},\text{C},\text{D})}/\partial {\beta }_{i}$ becomes zero. The condition that $({\beta }_{1},{\beta }_{2})$ satisfies the zero derivative is given as

17 $${\beta }_{2}=\frac{{\beta }_{1}{(1-{\beta }_{1})}^{3}}{{\beta }_{1}{(1-{\beta }_{1})}^{3}+{\mathscr{X}}({\alpha }_{1},{\alpha }_{2})},\,{\beta }_{1}=\frac{{\beta }_{2}{(1-{\beta }_{2})}^{3}}{{\beta }_{2}{(1-{\beta }_{2})}^{3}+{\mathscr{Y}}({\alpha }_{1},{\alpha }_{2})}.$$

In general, it is difficult to find $({\beta }_{1},{\beta }_{2})$ that satisfies Eq. (17). When ${q}_{1}={q}_{2}$ and ${\alpha }_{1}={\alpha }_{2}=\alpha$, $({\beta }_{1},{\beta }_{2})$ is analytically expressed as

18 $${\beta }_{1}={\beta }_{2}=1-\sqrt{\frac{|\langle {\psi }_{1}|{\psi }_{2}\rangle |}{1-\alpha }}=\beta .$$

Because ${\alpha }_{1}={\alpha }_{2}$ and ${\beta }_{1}={\beta }_{2}$, ${\gamma }_{1}={\gamma }_{2}$ also holds (see the Methods section). This condition is equal to that obtained by Bergou *et al*.^27^ In this case, the objective function of Eq. (16) is expressed as

19 $$\begin{array}{rcl}{P}_{s}^{(\text{B},\text{C},\text{D})} & = & \alpha \beta -|\langle {\psi }_{1}|{\psi }_{2}\rangle |\frac{\alpha \beta }{(1-\alpha )(1-\beta )}\\  & = & \alpha {\left\{1-\sqrt{\frac{|\langle {\psi }_{1}|{\psi }_{2}\rangle |}{1-\alpha }}\right\}}^{2}.\end{array}$$

**Figure 2:**
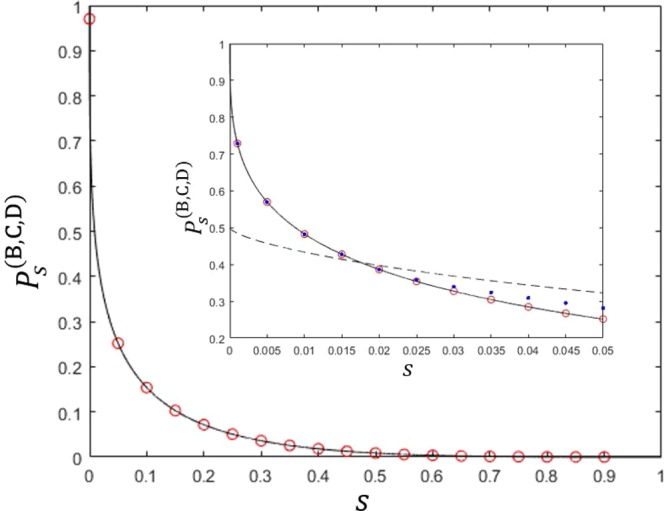
The success probability of generalized sequential state discrimination when Bob, Charlie, and David participate as receivers. Here, $s=|\langle {\psi }_{1}|{\psi }_{2}\rangle |$ denotes the overlap between two quantum states, and we use the identical prior probability (${q}_{1}={q}_{2}$). The small graph shows the optimal success probability in the region of $0 < s < 0.05$. The solid black line shows the optimal success probability when three receivers discriminate every two pure state of Alice’s^27^. The black dashed line as Eq. (21) shows the optimal success probability when three receivers discriminate only one of two pure states of Alice’s (Eq. (21) is a generalization of the result of Pang *et al*.^28^). The red circles and the blue dots display the optimal success probability from Eq. (16). More specifically, the red circles (the blue dots) shows the optimal success probability when the constraint conditions of ${\alpha }_{1}={\alpha }_{2}$ and ${\beta }_{1}={\beta }_{2}$ are (are not) added to Eq. (16). If $s\le 0.017559$, the red circles and the blue dots coincide with the solid black line, which shows that our result agrees with that of Bergou *et al*.^27^. Further, the blue dots are larger than the solid black line, but are smaller than the black dashed line. Therefore, if $s > 0.017559$, the optimal condition of generalized sequential state discrimination does not include the constraint conditions of ${\alpha }_{1}={\alpha }_{2}$ and ${\beta }_{1}={\beta }_{2}$.

We can easily check that Eq. (19) is maximized when $\alpha =1-{s}^{1/3}$ holds. In that case, an optimal success probability can be analytically described as ${P}_{s}^{(\text{B},\text{C},\text{D}),\text{opt}}={(1-{s}^{1/3})}^{3}$. This success probability is equal to the result of Bergou *et al*.^27^ However, this success probability is not optimal in general. That is because the equality condition $(\partial /\partial {\beta }_{1},\partial /\partial {\beta }_{2}){P}_{s}^{(\text{B},\text{C},\text{D})}=0$ does not guarantee optimum. Furthermore, we cannot confirm that the optimal condition satisfies the additional constraints ${\alpha }_{1}={\alpha }_{2}$ and ${\beta }_{1}={\beta }_{2}$. Therefore, we should check whether the maximum of Eq. (19) is really equal to that of Eq. (16). We plot the maxima of both Eqs. (16) and (19) in Fig. 2. In the Fig. 2, the solid black line shows the maximum of Eq. (19). The red circles (blue dots) shows the maximum of Eq. (16) with (without) the additional constraint ${\alpha }_{1}={\alpha }_{2}$ and ${\beta }_{1}={\beta }_{2}$. If the overlap $|\langle {\psi }_{1}|{\psi }_{2}\rangle |$ becomes smaller, the maxima of both Eqs. (16) and (19) become to coincide with. When the overlap is small, the optimal strategy of every receiver is to discriminate two pure states of Alice. Because the prior probabilities of two pure states are identical, the optimal measurement of every receiver does not show any bias to a specific pure state. Therefore, the condition of ${\alpha }_{1}={\alpha }_{2},{\beta }_{1}={\beta }_{2},{\gamma }_{1}={\gamma }_{2}$ should be included in the optimality conditions. If the overlap $|\langle {\psi }_{1}|{\psi }_{2}\rangle |$ becomes larger, the maximum of Eq. (16) becomes larger than that of Eq. (19). In this case, ${\alpha }_{1}={\alpha }_{2},{\beta }_{1}={\beta }_{2}$, and ${\gamma }_{1}={\gamma }_{2}$ are not optimal conditions anymore for the generalized sequential state discrimination. Especially, we observe that at least one of ${\gamma }_{1}$ and ${\gamma }_{2}$ becomes zero. This means that it is an optimal strategy when Bob, Charlie, and David discriminate one of Alice’s two pure states.

If Bob, Charlie, and David only discriminate one of Alice’s pure state, the generalized sequential state discrimination can be expressed as the following optimization problem:

20 $$\text{maximize}\,{P}_{s}^{(\text{B},\text{C},\text{D})}={q}_{i}{\alpha }_{i}{\beta }_{i}\left\{1-\frac{|\langle {\phi }_{1}^{(\text{B})}|{\phi }_{2}^{(\text{B})}\rangle {|}^{2}}{1-{\beta }_{i}}\right\}.$$

From the equality $\partial {P}_{s}^{(\text{B},\text{C},\text{D})}/\partial {\beta }_{i}=0$, we obtain ${\beta }_{i}=1-|\langle {\psi }_{1}|{\psi }_{2}\rangle |/\sqrt{(1-{\alpha }_{1})(1-{\alpha }_{2})}$. Substituting it into Eq. (20), we derive

21 $$\begin{array}{rcl}{P}_{s}^{(\text{B},\text{C},\text{D})} & = & {q}_{i}{\alpha }_{i}{\left\{1-\frac{|\langle {\psi }_{1}|{\psi }_{2}\rangle |}{\sqrt{(1-{\alpha }_{1})(1-{\alpha }_{2})}}\right\}}^{2}\\  & \le  & {q}_{i}{\alpha }_{i}{\left\{1-\frac{|\langle {\psi }_{1}|{\psi }_{2}\rangle |}{\sqrt{1-{\alpha }_{i}}}\right\}}^{2}\\  & \le  & {q}_{i}{(1-{s}^{2/3})}^{3}.\end{array}$$

The first inequality of Eq. (21) becomes equality at ${\alpha }_{j}=0(j\ne i)$. The second inequality becomes equality when ${\alpha }_{i}=1-|\langle {\psi }_{1}|{\psi }_{2}\rangle {|}^{2/3}$ holds. Therefore, the optimal success probability is given as ${P}_{s}^{(\text{B},\text{C},\text{D}),\text{opt}}=\text{max}\,\{{q}_{1},{q}_{2}\}{(1-|\langle {\psi }_{1}|{\psi }_{2}\rangle {|}^{2/3})}^{3}$, as shown in Fig. 2. In case of ${q}_{1}={q}_{2}$, when $|\langle {\psi }_{1}|{\psi }_{2}\rangle | < {({2}^{1/3}-1)}^{3}\simeq 0.0175599$ is satisfied, it is optimal for the three receivers to discriminate two of Alice’s pure states. However, if $|\langle {\psi }_{1}|{\psi }_{2}\rangle |\ge {({2}^{1/3}-1)}^{3}\simeq 0.0175599$ is fulfilled, discriminating only one of Alice’s pure state is optimal.

Next, we consider the case of $N=4$. In this case, let us denote the four receivers as Bob, Charlie, David, and Eliot. Each POVM of the four receivers corresponds to the two dimensional real vectors $({\alpha }_{1},{\alpha }_{2})$, $({\beta }_{1},{\beta }_{2})$, $({\gamma }_{1},{\gamma }_{2})$, and $({\delta }_{1},{\delta }_{2})$. According to Eq. (12), each real vector should satisfy

22 $$({\alpha }_{1},{\alpha }_{2})\in {C}_{\text{int}}^{(\text{B})},\,({\beta }_{1},{\beta }_{2})\in {C}_{\text{int}}^{(\text{C})},\,({\gamma }_{1},{\gamma }_{2})\in {C}_{\text{int}}^{(\text{D})},\,({\delta }_{1},{\delta }_{2})\in \partial {C}^{(\text{E})}.$$

Here, ${C}_{\text{int}}^{(\text{E})}$ and $\partial {C}^{(\text{E})}$ are respectively defined as

$$\begin{array}{rcl}{C}_{\text{int}}^{(\text{E})} & = & \{({\delta }_{1},{\delta }_{2})\in {{\mathbb{R}}}^{2}|(1-{\delta }_{1})(1-{\delta }_{2}) > |\langle {\phi }_{1}^{(\text{D})}|{\phi }_{2}^{(\text{D})}\rangle {|}^{2}\},\\ \partial {C}^{(\text{E})} & = & \{({\delta }_{1},{\delta }_{2})\in {{\mathbb{R}}}^{2}|(1-{\delta }_{1})(1-{\delta }_{2})=|\langle {\phi }_{1}^{(\text{D})}|{\phi }_{2}^{(\text{D})}\rangle {|}^{2}\}.\end{array}$$

Moreover, the POVM condition for Eliot is expressed as ${C}^{(\text{E})}={C}_{\text{int}}^{(\text{E})}\cup \partial {C}^{(\text{E})}$. Hence, we can obtain the following optimization problem:

23 $$\begin{array}{ll}\text{maximize} & {P}_{s}^{(\text{B},\text{C},\text{D})}={q}_{1}{\alpha }_{1}{\beta }_{1}{\gamma }_{1}{\delta }_{1}+{q}_{2}{\alpha }_{2}{\beta }_{2}{\gamma }_{2}{\delta }_{2}\\ \text{subject}\,\text{to} & (1-{\alpha }_{1})(1-{\alpha }_{2}) > |\langle {\psi }_{1}|{\psi }_{2}\rangle {|}^{2},\\  & (1-{\beta }_{1})(1-{\beta }_{2}) > |\langle {\phi }_{1}^{(\text{B})}|{\phi }_{2}^{(\text{B})}\rangle {|}^{2},\\  & (1-{\gamma }_{1})(1-{\gamma }_{2}) > |\langle {\phi }_{1}^{(\text{C})}|{\phi }_{2}^{(\text{C})}\rangle {|}^{2},\\  & (1-{\delta }_{1})(1-{\delta }_{2})=|\langle {\phi }_{1}^{(\text{D})}|{\phi }_{2}^{(\text{D})}\rangle {|}^{2}.\end{array}$$

Now, let us consider the equality constraint of Eq. (23). The optimal condition of the generalized sequential state discrimination for Eliot is given as a tangential point $({\delta }_{1},{\delta }_{2})$ between a plane ${P}_{s}^{(\text{B},\text{C},\text{D},\text{E})}=({q}_{1}{\alpha }_{1}{\beta }_{1}{\gamma }_{1}){\delta }_{1}+({q}_{2}{\alpha }_{2}{\beta }_{2}{\gamma }_{2}){\delta }_{2}$ and a surface $(1-{\delta }_{1})(1-{\delta }_{2})=|\langle {\phi }_{1}^{(\text{D})}|{\phi }_{2}^{(\text{D})}\rangle {|}^{2}$. If this tangential point is substituted into the objective function of Eq. (23), the following optimization problem can be obtained:

24 $$\begin{array}{ll}\text{maximize} & {P}_{s}^{(\text{B},\text{C},\text{D},\text{E})}={q}_{1}{\alpha }_{1}{\beta }_{1}{\gamma }_{1}+{q}_{2}{\alpha }_{2}{\beta }_{2}{\gamma }_{2}-2|\langle {\psi }_{1}|{\psi }_{2}\rangle |\sqrt{\frac{{q}_{1}{q}_{2}{\alpha }_{1}{\alpha }_{2}{\beta }_{1}{\beta }_{2}{\gamma }_{1}{\gamma }_{2}}{(1-{\alpha }_{1})(1-{\alpha }_{2})(1-{\beta }_{1})(1-{\beta }_{2})(1-{\gamma }_{1})(1-{\gamma }_{2})}}\\ \text{subject}\,\text{to} & (1-{\alpha }_{1})(1-{\alpha }_{2}) > |\langle {\psi }_{1}|{\psi }_{2}\rangle {|}^{2}\\  & (1-{\alpha }_{1})(1-{\alpha }_{2})(1-{\beta }_{1})(1-{\beta }_{2}) > |\langle {\psi }_{1}|{\psi }_{2}\rangle {|}^{2}\\  & {\gamma }_{2}\le \frac{{\gamma }_{1}(1-{\gamma }_{1})}{{\gamma }_{1}(1-{\gamma }_{1})+{\mathscr{X}}({\alpha }_{1},{\alpha }_{2},{\beta }_{1},{\beta }_{2})},\,{\mathscr{X}}({\alpha }_{1},{\alpha }_{2},{\beta }_{1},{\beta }_{2})=\frac{{q}_{2}{\alpha }_{2}{\beta }_{2}}{{q}_{1}{\alpha }_{1}{\beta }_{1}}\frac{|\langle {\psi }_{1}|{\psi }_{2}\rangle {|}^{2}}{(1-{\alpha }_{1})(1-{\alpha }_{2})(1-{\beta }_{1})(1-{\beta }_{2})}\\  & {\gamma }_{1}\le \frac{{\gamma }_{2}(1-{\gamma }_{2})}{{\gamma }_{2}(1-{\gamma }_{2})+{\mathscr{Y}}({\alpha }_{1},{\alpha }_{2},{\beta }_{1},{\beta }_{2})},\,{\mathscr{Y}}({\alpha }_{1},{\alpha }_{2},{\beta }_{1},{\beta }_{2})=\frac{{q}_{1}{\alpha }_{1}{\beta }_{1}}{{q}_{2}{\alpha }_{2}{\beta }_{2}}\frac{|\langle {\psi }_{1}|{\psi }_{2}\rangle {|}^{2}}{(1-{\alpha }_{1})(1-{\alpha }_{2})(1-{\beta }_{1})(1-{\beta }_{2})}\end{array}$$

**Figure 3:**
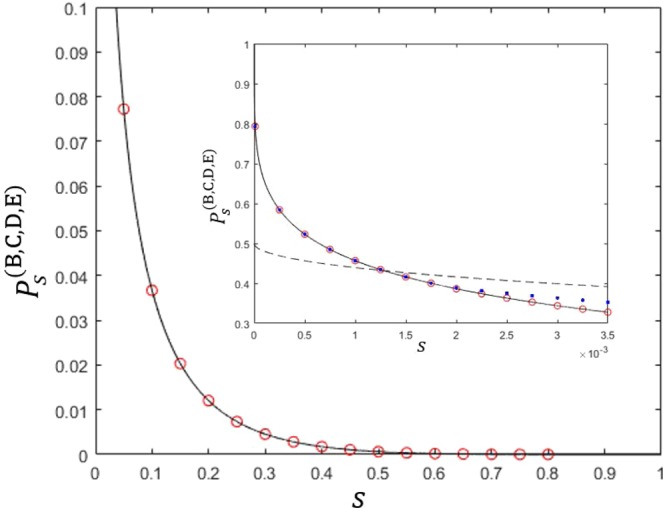
The success probability of generalized sequential state discrimination when Bob, Charlie, David, and Eliot participate as receivers. Here, $s=|\langle {\psi }_{1}|{\psi }_{2}\rangle |$ denotes the overlap between two quantum states, and we use the identical prior probability (${q}_{1}={q}_{2}$). The small graph shows the optimal success probability in the region of $0 < s < 0.0035$. The solid black line shows the optimal success probability when four receivers discriminate every two pure state of Alice’s^27^. The black dashed line as Eq. (26) shows the optimal success probability when four receivers discriminate only one of two pure states of Alice’s (Eq. (26) is a generalization of the result of Pang *et al*.^28^). The red circles and the blue dots display the optimal success probability from Eq. (24). More specifically, the red circles (the blue dots) shows the optimal success probability when the constraint conditions of ${\alpha }_{1}={\alpha }_{2}$, ${\beta }_{1}={\beta }_{2}$, and ${\gamma }_{1}={\gamma }_{2}$ are (are not) added to Eq. (16). If $s\le 0.001282$, the red circles and the blue dots coincide with the solid black line, which shows that our result agrees with that of Bergou *et al*.^27^. Further, the blue dots are larger than the solid black line, but are smaller than the black dashed line. Therefore, if $s > 0.001282$, the optimal condition of generalized sequential state discrimination does not include the constraint conditions of ${\alpha }_{1}={\alpha }_{2}$, ${\beta }_{1}={\beta }_{2}$, and ${\gamma }_{1}={\gamma }_{2}$.

The detailed derivation of Eq. (24) is provided in the Methods section. When the constraints ${\alpha }_{1}={\alpha }_{2},{\beta }_{1}={\beta }_{2},{\gamma }_{1}={\gamma }_{2}$, and ${\delta }_{1}={\delta }_{2}$ are added, this optimization problem is difficult to solve analytically. Thus we solve this problem numerically. We show the maximum of Eq. (24) in Fig. 3. When the constraints ${\alpha }_{1}={\alpha }_{2},{\beta }_{1}={\beta }_{2},{\gamma }_{1}={\gamma }_{2}$, and ${\delta }_{1}={\delta }_{2}$ are added to Eq. (24), the optimal success probability becomes equal to the result by Bergou *et al*.^27^ If the overlap is small, this equality constraint is included in the optimal condition of sequential state discrimination. Because the prior probabilities of two pure states are identical, the optimal measurement of every receiver does not show any bias to a specific pure state. If we do not add these constraints, the optimal success probability becomes larger than that provided by Bergou *et al*.^27^. In this case, we observe that at least one of ${\delta }_{1}$ and ${\delta }_{2}$ becomes zero. This implies that it is optimal only when four receivers discriminate only one out of two pure states of Alice.

If Bob, Charlie, David, and Eliot only discriminate one out of two pure states, the generalized sequential state discrimination is described as the following optimization problem:

25 $$\text{maximize}\,{P}_{s}^{(\text{B},\text{C},\text{D},\text{E})}={q}_{i}{\alpha }_{i}{\beta }_{i}{\left\{1-\frac{|\langle {\phi }_{1}^{(\text{B})}|{\phi }_{2}^{(\text{B})}\rangle |}{\sqrt{(1-{\beta }_{1})(1-{\beta }_{2})}}\right\}}^{2}.$$

The success probability satisfies the following inequalities:

26 $$\begin{array}{rcl}{P}_{s}^{(\text{B},\text{C},\text{D},\text{E})} & \le  & {q}_{i}{\alpha }_{i}{\beta }_{i}{\left\{1-\frac{|\langle {\phi }_{1}^{(\text{B})}|{\phi }_{2}^{(\text{B})}\rangle |}{\sqrt{1-{\beta }_{i}}}\right\}}^{2}\\  & \le  & {q}_{i}{\alpha }_{i}{(1-|\langle {\phi }_{1}^{(\text{B})}|{\phi }_{2}^{(\text{B})}\rangle {|}^{2/3})}^{3}\\  & = & {q}_{i}{\alpha }_{i}{\left\{1-{\left(\frac{|\langle {\psi }_{1}|{\psi }_{2}\rangle |}{\sqrt{(1-{\alpha }_{1})(1-{\alpha }_{2})}}\right)}^{2/3}\right\}}^{3}\\  & \le  & {q}_{i}{\alpha }_{i}{\left\{1-{\left(\frac{|\langle {\psi }_{1}|{\psi }_{2}\rangle |}{\sqrt{1-{\alpha }_{i}}}\right)}^{2/3}\right\}}^{3}\\  & \le  & {q}_{i}{(1-\sqrt{|\langle {\psi }_{1}|{\psi }_{2}\rangle |})}^{4}\end{array}$$

In Eq. (26), the first inequality becomes equality at ${\beta }_{j}=0(j\ne i)$. Likewise, the third inequality becomes equality at ${\alpha }_{j}=0(j\ne i)$. The second and fourth inequality become zero when the partial derivatives $\partial {P}_{s}^{(\text{B},\text{C},\text{D},\text{E})}/\partial {\alpha }_{i}$ and $\partial {P}_{s}^{(\text{B},\text{C},\text{D},\text{E})}/\partial {\beta }_{i}$ become zero. Therefore, the maximum of Eq. (25) becomes ${P}_{s}^{(\text{B},\text{C},\text{D},\text{E}),\text{opt}}=\text{max}\{{q}_{1},{q}_{2}\}(1-\sqrt{|\langle {\psi }_{1}|{\psi }_{2}\rangle |})$. When ${q}_{1}={q}_{2}$, if $|\langle {\psi }_{1}|{\psi }_{2}\rangle | < {({2}^{1/4}-1)}^{4}\simeq 0.00128159$, it is optimal for the four receivers to discriminate every two pure state of Alice. If $|\langle {\psi }_{1}|{\psi }_{2}\rangle |\ge {({2}^{1/4}-1)}^{4}\simeq 0.00128159$, it is optimal for the four receivers to discriminate only one out of two pure states.

From the previous results, we can consider the generalized sequential state discrimination for arbitrary $N$, in an inductive manner. We can construct an optimization problem of the generalized sequential state discrimination for any $N$ as follows:

27 $$\begin{array}{ll}\text{maximize} & {P}_{s}^{({B}_{1},\ldots ,{B}_{N})}={q}_{1}\,\mathop{\prod }\limits_{I=1}^{N-1}\,{\alpha }_{1}^{(I)}+{q}_{2}\,\mathop{\prod }\limits_{I=1}^{N-1}\,{\alpha }_{2}^{(I)}-2|\langle {\psi }_{1}|{\psi }_{2}\rangle |\sqrt{{q}_{1}{q}_{2}}\,\mathop{\prod }\limits_{I=1}^{N-1}\,\sqrt{\frac{{\alpha }_{1}^{(I)}{\alpha }_{2}^{(I)}}{(1-{\alpha }_{1}^{(I)})(1-{\alpha }_{2}^{(I)})}}\\ \text{subject}\,\text{to} & \mathop{\prod }\limits_{I=1}^{J}\,(1-{\alpha }_{1}^{(I)})(1-{\alpha }_{2}^{(I)}) > |\langle {\psi }_{1}|{\psi }_{2}\rangle {|}^{2}\,\forall J\in \{1,\ldots ,N-2\}\\  & {\alpha }_{2}^{(N-1)}\le \frac{{\alpha }_{1}^{(N-1)}(1-{\alpha }_{1}^{(N-1)})}{{\alpha }_{1}^{(N-1)}(1-{\alpha }_{1}^{(N-1)})+{\mathscr{X}}(\overrightarrow{\alpha },\overrightarrow{\beta })},\,{\mathscr{X}}(\overrightarrow{\alpha },\overrightarrow{\beta })=\frac{{q}_{2}}{{q}_{1}}\left(\mathop{\prod }\limits_{I=1}^{N-1}\,\frac{{\alpha }_{2}^{(I)}}{{\alpha }_{1}^{(I)}}\right)\frac{|\langle {\psi }_{1}|{\psi }_{2}\rangle {|}^{2}}{{\prod }_{I{\prime} =1}^{N-1}\,(1-{\alpha }_{1}^{(I{\prime} )})(1-{\alpha }_{2}^{(I{\prime} )})}\\  & {\alpha }_{1}^{(N-1)}\le \frac{{\alpha }_{2}^{(N-1)}(1-{\alpha }_{2}^{(N-1)})}{{\alpha }_{2}^{(N-1)}(1-{\alpha }_{2}^{(N-1)})+{\mathscr{Y}}(\overrightarrow{\alpha },\overrightarrow{\beta })}.\,{\mathscr{Y}}(\overrightarrow{\alpha },\overrightarrow{\beta })=\frac{{q}_{1}}{{q}_{2}}\left(\mathop{\prod }\limits_{I=1}^{N-1}\,\frac{{\alpha }_{1}^{(I)}}{{\alpha }_{2}^{(I)}}\right)\frac{|\langle {\psi }_{1}|{\psi }_{2}\rangle {|}^{2}}{{\prod }_{I{\prime} =1}^{N-1}\,(1-{\alpha }_{1}^{(I{\prime} )})(1-{\alpha }_{2}^{(I{\prime} )})}\end{array}$$

where $\overrightarrow{\alpha }=({\alpha }_{1}^{(1)},{\alpha }_{2}^{(1)},\cdots ,{\alpha }_{1}^{(N)},{\alpha }_{2}^{(N)})$ and $\overrightarrow{\beta }=({\beta }_{1}^{(1)},{\beta }_{2}^{(1)},\cdots ,{\beta }_{1}^{(N)},{\beta }_{2}^{(N)})$. If prior probabilities are all equal, the optimal success probability is obtained as

28 $$\begin{array}{rcl}{P}_{s}^{({B}_{1},\ldots ,{B}_{N}),\text{opt}} & = & {(1-|\langle {\psi }_{1}|{\psi }_{2}\rangle {|}^{1/N})}^{N},\,|\langle {\psi }_{1}|{\psi }_{2}\rangle | < S(N)\\ {P}_{s}^{({B}_{1},\ldots ,{B}_{N}),\text{opt}} & = & \frac{1}{2}{(1-|\langle {\psi }_{1}|{\psi }_{2}\rangle {|}^{2/N})}^{N}.\,|\langle {\psi }_{1}|{\psi }_{2}\rangle |\ge S(N)\end{array}$$

where $S(N)={({2}^{1/N}-1)}^{N}$. Equation (28) satisfies the result of Bergou^27^ and Pang^28^. If $|\langle {\psi }_{1}|{\psi }_{2}\rangle | < S(N)$, the optimal condition contains ${\alpha }_{1}^{(I)}={\alpha }_{2}^{(I)}(\forall I)$. This property was argued by Bergou *et al*.^27^ When $N\in \{2,3,\ldots ,7\}$, the value of $S(N)$ is numerically given as

$$\begin{array}{rcl}S(2) & = & 0.171572875253810\\ S(3) & = & 0.017559993780021\\ S(4) & = & 0.001281592197970\\ S(5) & = & 7.269939897187259\times {10}^{-5}\\ S(6) & = & 3.372943879071272\times {10}^{-6}\\ S(7) & = & 1.323880715612381\times {10}^{-7}\end{array}$$

When $N$ becomes larger, $S(N)$ rapidly converges to zero. This implies that when the overlap between two pure states is not small enough, discriminating two pure states when many receivers participate is not a good strategy. Therefore, sequential state discrimination can be applicable for multiparty QKD in the case of a suitable number of receivers. That is because, in multiparty QKD, all receivers are required to discriminate every quantum state^24,27^.

#### Generalized sequential state discrimination of two mixed states

Here, let us consider the generalized sequential state discrimination of two mixed states. For convenience, we will consider rank-2 mixed states such as

29 $$\begin{array}{c}{\rho }_{1}={r}_{1}|{r}_{1}\rangle \langle {r}_{1}|\oplus {\bar{r}}_{1}|{\bar{r}}_{1}\rangle \langle {\bar{r}}_{1}|,\\ {\rho }_{2}={r}_{2}|{r}_{2}\rangle \langle {r}_{2}|\oplus {\bar{r}}_{2}|{\bar{r}}_{2}\rangle \langle {\bar{r}}_{2}|.\end{array}$$

Equation (29) has the same form as that of Eq. (6). Like in Eq. (6), every POVM corresponds to a real vector $({\alpha }_{1}^{(I)},{\alpha }_{2}^{(I)},{\bar{\alpha }}_{1}^{(I)},{\bar{\alpha }}_{2}^{(I)})$. We can describe the generalized sequential state discrimination of two mixed states as the following optimization problem^33^:

30 $$\begin{array}{ll}\text{maximize} & {P}_{s}^{({B}_{1},\ldots ,{B}_{N})}={q}_{1}({r}_{1}\mathop{\prod }\limits_{I=1}^{N}{\alpha }_{1}^{(I)}+{\bar{r}}_{1}\mathop{\prod }\limits_{I=1}^{N}{\bar{\alpha }}_{1}^{(I)})+{q}_{2}({r}_{2}\mathop{\prod }\limits_{I=1}^{N}{\alpha }_{2}^{(I)}+{\bar{r}}_{2}\mathop{\prod }\limits_{I=1}^{N}{\bar{\alpha }}_{2}^{(I)})\\ \text{subject}\,\text{to} & (1-{\alpha }_{1}^{(1)})(1-{\alpha }_{2}^{(1)}) > |\langle {r}_{1}|{r}_{2}\rangle {|}^{2},\,(1-{\bar{\alpha }}_{1}^{(1)})(1-{\bar{\alpha }}_{2}^{(1)}) > |\langle {\bar{r}}_{1}|{\bar{r}}_{2}\rangle {|}^{2}\\  & (1-{\alpha }_{1}^{(I+1)})(1-{\alpha }_{2}^{(I+1)}) > |\langle {s}_{1}^{(I)}|{s}_{2}^{(I)}\rangle {|}^{2},\,(1-{\bar{\alpha }}_{1}^{(I+1)})(1-{\bar{\alpha }}_{2}^{(I+1)}) > |\langle {\bar{s}}_{1}^{(I)}|{\bar{s}}_{2}^{(I)}\rangle {|}^{2},\,\forall I\in \{1,N-2\}(1-{\alpha }_{1}^{(N)})(1-{\alpha }_{2}^{(N)})\\  & =|\langle {s}_{1}^{(N-1)}|{s}_{2}^{(N-1)}\rangle {|}^{2},\,(1-{\bar{\alpha }}_{1}^{(N)})(1-{\bar{\alpha }}_{2}^{(N)})=|\langle {\bar{s}}_{1}^{(N-1)}|{\bar{s}}_{2}^{(N-1)}\rangle {|}^{2}\end{array}$$

Then, Eq. (30) can be divided into the following two optimization problems:

$$\begin{array}{ll}\text{maximize} & {p}_{s}={q}_{1}{r}_{1}\mathop{\prod }\limits_{I=1}^{N}{\alpha }_{1}^{(I)}+{q}_{2}{r}_{2}\mathop{\prod }\limits_{I=1}^{N}{\alpha }_{2}^{(I)}\\ \text{subject}\,\text{to} & (1-{\alpha }_{1}^{(1)})(1-{\alpha }_{2}^{(1)}) > |\langle {r}_{1}|{r}_{2}\rangle {|}^{2}\\  & (1-{\alpha }_{1}^{(I+1)})(1-{\alpha }_{2}^{(I+1)}) > |\langle {s}_{1}^{(I)}|{s}_{2}^{(I)}\rangle {|}^{2},\,\forall \,I\in \{1,\ldots ,N-2\}\\  & (1-{\alpha }_{1}^{(N)})(1-{\alpha }_{2}^{(N)})=|\langle {s}_{1}^{(N-1)}|{s}_{2}^{(N-1)}\rangle {|}^{2}.\end{array}$$

$$\begin{array}{ll}\text{maximize} & {\bar{p}}_{s}={q}_{1}{\bar{r}}_{1}\mathop{\prod }\limits_{I=1}^{N}{\bar{\alpha }}_{1}^{(I)}+{q}_{2}{\bar{r}}_{2}\mathop{\prod }\limits_{I=1}^{N}{\bar{\alpha }}_{2}^{(I)}\\ \text{subject}\,\text{to} & (1-{\bar{\alpha }}_{1}^{(1)})(1-{\bar{\alpha }}_{2}^{(1)}) > |\langle {\bar{r}}_{1}|{\bar{r}}_{2}\rangle {|}^{2}\\  & (1-{\bar{\alpha }}_{1}^{(I+1)})(1-{\bar{\alpha }}_{2}^{(I+1)}) > |\langle {\bar{s}}_{1}^{(I)}|{\bar{s}}_{2}^{(I)}\rangle {|}^{2},\,\forall \,I\in \{1,\ldots ,N-2\}\\  & (1-{\bar{\alpha }}_{1}^{(N)})(1-{\bar{\alpha }}_{2}^{(N)})=|\langle {\bar{s}}_{1}^{(N-1)}|{\bar{s}}_{2}^{(N-1)}\rangle {|}^{2}.\end{array}$$

That is, the maximum of Eq. (30) becomes $\text{max}\,{P}_{s}^{({B}_{1},\ldots ,{B}_{N}),\text{opt}}=\text{max}\,{p}_{s}+\text{max}\,{\bar{p}}_{s}$. Although we consider rank-2 mixed states as Eq. (29), we can generalize this argument to any mixed states, with an arbitrary rank. If ${q}_{1}={q}_{2}$, ${r}_{1}={r}_{2}=r$, and ${\bar{r}}_{1}={\bar{r}}_{2}=\bar{r}$ are assumed, then the optimal success probability can be found as^33^

$$\begin{array}{ll}\text{max}\,{P}_{s}^{({B}_{1},\cdots ,{B}_{N})}=r{(1-|\langle {r}_{1}|{r}_{2}\rangle {|}^{1/N})}^{N}+\bar{r}{(1-|\langle {\bar{r}}_{1}|{\bar{r}}_{2}\rangle {|}^{1/N})}^{N}, & |\langle {r}_{1}|{r}_{2}\rangle | < S(N)\wedge |\langle {\bar{r}}_{1}|{\bar{r}}_{2}\rangle | < S(N)\\ \text{max}\,{P}_{s}^{({B}_{1},\ldots ,{B}_{N})}=r{(1-|\langle {r}_{1}|{r}_{2}\rangle {|}^{1/N})}^{N}+\bar{r}\frac{1}{2}{(1-|\langle {\bar{r}}_{1}|{\bar{r}}_{2}\rangle {|}^{2/N})}^{N}, & |\langle {r}_{1}|{r}_{2}\rangle | < S(N)\wedge |\langle {\bar{r}}_{1}|{\bar{r}}_{2}\rangle |\ge S(N)\\ \text{max}\,{P}_{s}^{({B}_{1},\ldots ,{B}_{N})}=r\frac{1}{2}{(1-|\langle {r}_{1}|{r}_{2}\rangle {|}^{2/N})}^{N}+\bar{r}{(1-|\langle {\bar{r}}_{1}|{\bar{r}}_{2}\rangle {|}^{1/N})}^{N}, & |\langle {r}_{1}|{r}_{2}\rangle |\ge \bar{S}(N)\wedge |\langle {\bar{r}}_{1}|{\bar{r}}_{2}\rangle | < S(N)\\ \text{max}\,{P}_{s}^{({B}_{1},\ldots ,{B}_{N})}=r\frac{1}{2}{(1-|\langle {r}_{1}|{r}_{2}\rangle {|}^{2/N})}^{N}+\bar{r}\frac{1}{2}{(1-|\langle {\bar{r}}_{1}|{\bar{r}}_{2}\rangle {|}^{2/N})}^{N}. & |\langle {r}_{1}|{r}_{2}\rangle |\ge S(N)\wedge |\langle {\bar{r}}_{1}|{\bar{r}}_{2}\rangle |\ge S(N)\end{array}$$

For either $${q}_{1}\ne {q}_{2}$$ or $${r}_{1}\ne {r}_{2}$$($${\bar{r}}_{1}={\bar{r}}_{2}$$), when $$|\langle {r}_{1}|{r}_{2}\rangle | < S(N)$$($$|\langle {\bar{r}}_{1}|{\bar{r}}_{2}\rangle | < S(N)$$), $$\text{max}\,{p}_{s}$$($$\text{max}\,{\bar{p}}_{s}$$) is difficult to obtain analytically. Therefore, one may evaluate $$\text{max}\,{p}_{s}$$($$\text{max}\,{\bar{p}}_{s}$$) numerically. However, when $$|\langle {r}_{1}|{r}_{2}\rangle |\ge S(N)$$$$(|\langle {\bar{r}}_{1}|{\bar{r}}_{2}\rangle |\ge S(N))$$, one can obtain $$\text{max}\,{p}_{s}=\text{max}\,\{{q}_{1}{r}_{1},{q}_{2}{r}_{2}\}{(1-|\langle {r}_{1}|{r}_{2}\rangle {|}^{2/N})}^{N}$$($$\text{max}\,{\bar{p}}_{s}=\text{max}\,\{{q}_{1}{\bar{r}}_{1},{q}_{2}{\bar{r}}_{2}\}{(1-|\langle {\bar{r}}_{1}|{\bar{r}}_{2}\rangle {|}^{2/N})}^{N}$$).

If $|\langle {r}_{1}|{r}_{2}\rangle |\ge S(N)$ and $|\langle {\bar{r}}_{1}|{\bar{r}}_{2}\rangle |\ge S(N)$, it is optimal that every receiver discriminates Alice’s two pure state. However, If $|\langle {r}_{1}|{r}_{2}\rangle | < S(N)$ or $|\langle {\bar{r}}_{1}|{\bar{r}}_{2}\rangle | < S(N)$, discriminating only one out of Alice’s two pure states is optimal.

#### Comparison with other discrimination strategies

**Figure 4:**
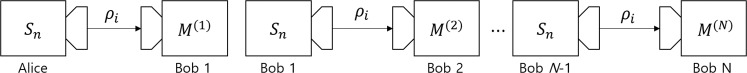
Schematic of the quantum reproducing strategy for Bob 1, Bob 2, $\cdots$, and Bob $N-1$.

There are other strategies for multi-party QKD, besides sequential state discrimination. The first one is the quantum reproducing strategy^27^. This strategy is performed as in the following procedure (see Fig. 4): Bob 1 optimally discriminates Alice’s quantum states, without any error. If Bob 1 obtains a conclusive result, he can reproduce the same quantum state, which corresponds to his conclusive result. Then, Bob 1 sends the quantum state to Bob 2. This procedure is recursively performed from Bob 2 to Bob $N$. If Bob $I$ obtains an inconclusive result, he tells every receiver, through classical communication, that he failed to discriminate the quantum state of Alice. The success probability of the quantum reproducing strategy is expressed as

$$\begin{array}{rcl}{P}_{s,rep}^{({B}_{1},\ldots ,{B}_{N})} & = & {q}_{1}{\text{Pr}}_{{B}_{1}}[1|{\rho }_{1}]{\text{Pr}}_{{B}_{2}}[1|{\rho }_{1}]\times \cdots \times {\text{Pr}}_{{B}_{N}}[1|{\rho }_{1}]\\  &  & +\,{q}_{2}{\text{Pr}}_{{B}_{1}}[2|{\rho }_{2}]{\text{Pr}}_{{B}_{2}}[2|{\rho }_{2}]\times \cdots \times {\text{Pr}}_{{B}_{N}}[2|{\rho }_{2}].\end{array}$$

Here, let us consider the case of ${S}_{2}$. Then, ${\text{Pr}}_{{B}_{I}}[i|{\rho }_{i}]=\text{Tr}[{\rho }_{i}{M}_{i}^{(I)}]$ is the probability that Bob $I$ obtains the measurement outcome $i$. If we assume an equal prior probability, when mixed states have the same eigenvalues, the optimal success probability of the quantum reproducing strategy is derived as (see Method)^21^

$${P}_{rep.}^{({B}_{1},\ldots ,{B}_{N}),\text{opt}}={(1-rs-\bar{r}\bar{s})}^{N}.$$

Here, ${r}_{1}={r}_{2}=r$ and ${\bar{r}}_{1}={\bar{r}}_{2}=\bar{r}$. $s$ and $\bar{s}$ are defined as $s=|\langle {r}_{1}|{r}_{2}\rangle |$ and $\bar{s}=|\langle {\bar{r}}_{1}|{\bar{r}}_{2}\rangle |$, respectively.

**Figure 5:**
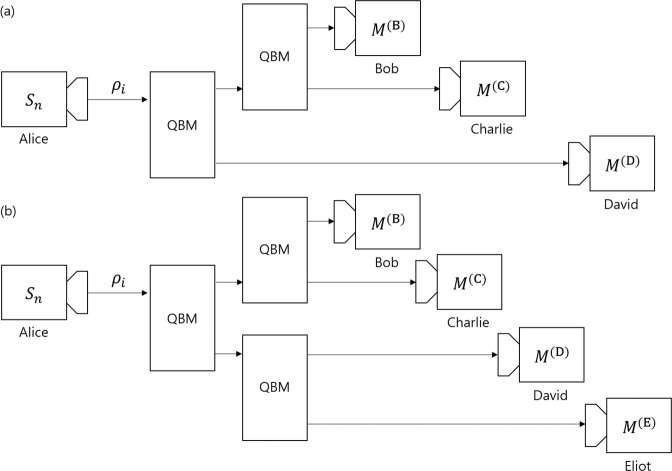
Schematic of the quantum broadcasting strategy. Here, QBM denotes a quantum broadcasting machine^39^. (**a**) consists of Bob, Charlie, and David. (**b**) consists of Bob, Charlie, David, and Eliot.

The second case is the quantum broadcasting strategy^27,32^. This strategy is performed as in the following process (see Fig. 5): Bob 1 puts Alice’s unknown quantum states into a quantum broadcasting machine, which transforms ${\rho }_{i}$ into an $N-$partite state ${\sigma }_{i}^{({B}_{1},\ldots ,{B}_{N})}$, with a probability of less than 1^39^. Here, these states satisfy ${\text{Tr}}_{{B}_{1},\ldots ,{B}_{I-1},{B}_{I+1},\ldots ,{B}_{N}}[{\sigma }_{i}^{({B}_{1},\ldots ,{B}_{N})}]={\rho }_{i}(\,\forall \,I)$. If Bob 1 succeeds in quantum broadcasting, Then, Bob 1, Bob 2,$\cdots$, and Bob $N$ can share ${\sigma }_{i}^{({B}_{1},\ldots ,{B}_{N})}$. Then, every receiver discriminates his partial state ${\rho }_{i}$, without any error. If Bob 1 fails, however, he tells everyone, through classical communication, that quantum broadcasting has failed. The optimal success probability of quantum broadcasting is expressed as

$$\begin{array}{rcl}{P}_{s,broad}^{({B}_{1},\ldots ,{B}_{N})} & = & {q}_{1}{\text{Pr}}_{broad}[{\rho }_{1}]{\text{Pr}}_{{B}_{1}}[1|{\rho }_{1}]{\text{Pr}}_{{B}_{2}}[1|{\rho }_{1}]\times \cdots \times {\text{Pr}}_{{B}_{N}}[1|{\rho }_{1}]\\  &  & +\,{q}_{2}{\text{Pr}}_{broad}[{\rho }_{2}]{\text{Pr}}_{{B}_{1}}[2|{\rho }_{2}]{\text{Pr}}_{{B}_{2}}[2|{\rho }_{2}]\times \cdots \times {\text{Pr}}_{{B}_{N}}[2|{\rho }_{2}]\end{array}$$

Here, ${\text{Pr}}_{broad}[{\rho }_{i}]$ is the maximal success probability to succeed in broadcasting when ${\rho }_{i}$ is given. The optimal success probability of the quantum broadcasting strategy is larger than that of the quantum reproducing strategy. In Fig. 5, quantum broadcasting strategies for three receivers (Bob, Charlie, and David) and four receivers (Bob, Charlie, David, and Eliot) are described. In these cases, the optimal success probabilities of the quantum broadcast strategy are given by

$$\begin{array}{rcl}{P}_{s,broad}^{(\text{B},\text{C},\text{D}),\text{opt}} & = & \text{min}{\left\{\frac{1}{1+s},\frac{1}{1+\bar{s}}\right\}}^{2}{(1-rs-\bar{r}\bar{s})}^{3},\\ {P}_{s,broad}^{(\text{B},\text{C},\text{D},\text{E}),\text{opt}} & = & \text{min}{\left\{\frac{1}{1+s},\frac{1}{1+\bar{s}}\right\}}^{3}{(1-rs-\bar{r}\bar{s})}^{4}.\end{array}$$

Here, $\text{min}\left\{\frac{1}{1+s},\frac{1}{1+\bar{s}}\right\}$ is the optimal probability that once quantum broadcasting succeeds^39^. Because ${\text{Pr}}_{broad}[{\rho }_{1}]={\text{Pr}}_{broad}[{\rho }_{2}]$, the optimal success probability of quantum broadcasting can be derived, similar to that of quantum reproducing. If Alice prepares one out of two pure states, the optimal success probability of the generalized sequential state discrimination is less than that of quantum reproducing or quantum broadcasting^27^. However, if Alice prepares one out of two mixed states, the optimal success probability of the generalized sequential state discrimination can be larger than that of both the quantum reproducing and the quantum broadcasting strategy. Namely, the generalized sequential state discrimination of mixed states has more potential for application to multiparty QKD than those of the quantum reproducing and quantum broadcasting strategies. It should be noted that sequential state discrimination can be a good candidate for application to multiparty QKD when mixed states are used, in contrast to the result of the pure states obtained by Bergou *et al*.^27^.

##### Example 1.

Suppose that Alice prepares one out of two mixed states ${\rho }_{i}\in \{{\rho }_{1},{\rho }_{2}\}$, with equal prior probabilities. Here, two mixed states are expressed as Eq. (29), where $r=0.6,\bar{r}=1-r=0.4$, $s=0.7$, and $\bar{s}=0.001$. In the case of $N=3$(Bob,Charlie, and David), the optimal success probability for each strategy is numerically obtained as

$$\begin{array}{c}\text{max}\,{P}_{seq}^{(\text{B},\text{C},\text{D})}=0.294443356418367,\\ \text{max}\,{P}_{rep}^{(\text{B},\text{C},\text{D})}=0.194708598336000,\\ \text{max}\,{P}_{broad}^{(\text{B},\text{C},\text{D})}=0.067373217417301.\end{array}$$

Here, ${P}_{seq}$, ${P}_{rep}$, and ${P}_{rep}$ denote the success probability of generalized sequential state discrimination, quantum reproducing, and quantum broadcasting, respectively. In the case of $N=4$(Bob, Charlie, David, and Eliot), optimal success probability for each strategy is numerically obtained as

$$\begin{array}{c}\text{max}\,{P}_{seq}^{(\text{B},\text{C},\text{D},\text{E})}=0.182986042905254,\\ \text{max}\,{P}_{rep}^{(\text{B},\text{C},\text{D},\text{E})}=0.112853103595546,\\ \text{max}\,{P}_{broad}^{(\text{B},\text{C},\text{D},\text{E})}=0.022970304008863.\end{array}$$

Therefore, it can be seen that, when two mixed states such as Eq. (29), with $r=0.6$, $\bar{r}=0.7$, $s=0.7$, and $\bar{s}=0.001$, are used, the optimal success probability of generalized sequential state discrimination performs better than quantum reproducing and quantum broadcasting.

##### Example 2.

**Figure 6:**
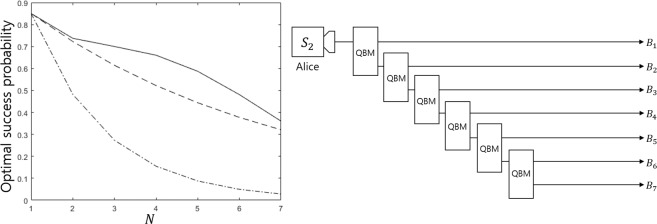
Plot of the optimal success probability of generalized sequential state discrimination (solid black line), quantum reproducing strategy (black dashed line) and quantum broadcasting strategy (dash-dot black line). We also describe a specific way to perform quantum broadcasting strategy.

Suppose Alice prepares one out of two mixed states ${\rho }_{i}\in \{{\rho }_{1},{\rho }_{2}\}$, with equal prior probabilities. Here, two mixed states are expressed as Eq. (29), where $r=0.3,\bar{r}=1-r=0.7$, $s=0.5$, and $\bar{s}=5\times {10}^{-8}$. We plotted the optimal success probability of generalized sequential state discrimination and quantum reproducing strategy in Fig. 6. As can be seen in the figure, the optimal success probability of generalized sequential state discrimination exceeds those of the quantum reproducing and quantum broadcasting strategies. Here, we describe a specific way to perform the quantum broadcasting strategy, as shown in Fig. 6, when seven receivers participate. When the given quantum states are non-orthogonal, because the success probability of quantum broadcasting cannot achieve to one, generalized sequential state discrimination also outperforms quantum broadcasting strategy. In the extreme case, if $N=1$, the optimal success probability in Fig. 6 becomes equal to the optimal unambiguous discrimination^21^.

In conclusion, the generalized sequential state discrimination of two mixed states can outperform the other two strategies. It can be implemented using linear optics. In the next section, we will describe its implementation in detail.

### Optical implementation

Here, we explain the method to implement the generalized sequential state discrimination of two coherent states, using linear optics.

#### Implementation of the POVM for unambiguous discrimination

Suppose that Alice prepares $|{\psi }_{i}\rangle \in {\bar{S}}_{n}$, with a prior probability ${q}_{i}$. When an ancilla state $|{\mathscr{B}}\rangle$ is prepared in Bob, his measurement that performs an unambiguous discrimination can be constructed as

31 $${U}_{\text{AB}}^{(\text{B})}|{\psi }_{i}{\rangle }_{A}\otimes |{\mathscr{B}}{\rangle }_{B}=\sqrt{{\alpha }_{i}}|{\phi }_{i}{\rangle }_{A}\otimes |{{\mathscr{B}}}_{i}{\rangle }_{B}+\sqrt{{\bar{\alpha }}_{i}}|{\phi }_{0}{\rangle }_{A}\otimes |{{\mathscr{B}}}_{0}{\rangle }_{B}.$$

Here, $\{|{{\mathscr{B}}}_{0}\rangle ,|{{\mathscr{B}}}_{1}\rangle ,\ldots ,|{{\mathscr{B}}}_{n}\rangle \}$ is an orthonormal basis. Moreover, ${\alpha }_{i}$(${\bar{\alpha }}_{i}$) is a conditional probability, that Bob obtains conclusive result $i$(inconclusive result), when Alice prepares ${\psi }_{i}$. It is well known that Eq. (31) is equivalent to Eq. (2), in unambiguous discrimination.Moreover, Eq. (31) shows a way to implement an unambiguous discrimination in real-world settings.

If ${\bar{S}}_{2}$ consists of two polarized single photon states $|{\psi }_{i}\rangle ={a}_{i}|H\rangle +{b}_{i}|V\rangle$($i=1,2$), a global unitary operator ${U}_{AB}^{(\text{B})}$ can be implemented using linear optics. Solis-Prosser *et al*.^29^ used this method to perform a sequential state discrimination of two polarized single-photon states, with equal prior probability. In their model, an ancilla state in Eq. (31) corresponds to a single-photon path. If their model is applied to generalized sequential state discrimination, Bob $I$ should prepare ${3}^{I}$ paths. Therefore, if the number of receivers is large, generalized sequential state discrimination should require an exponentially large number of single photon paths.

However, the sequential state discrimination of coherent states does not require many paths^34^. One can perform the sequential state discrimination by using the modified Banaszek or the Huttner model. In the next subsection. we will explain the modified Banaszek model)^40,41^. Both models can perform an unambiguous discrimination of two nonorthogonal coherent states, with general prior probabilities. Furthermore, both models can achieve an IDP limit^8–11^. By simply adding beamsplitters in the Banaszek or the Huttner model, one can perform a generalized measurement, that produces post-measurement states^34^.

#### Implementation of the sequential state discrimination of two pure states

**Figure 7:**
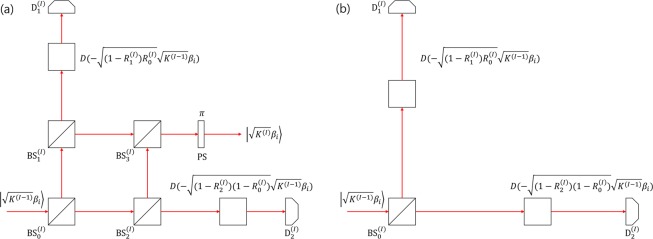
Schematic of the generalized sequential state discrimination based on the Banaszek model^40^. (**a**) Shows the measurement for Bob $I < N$. (**b**) Shows the measurement for Bob $N$. ${\text{BS}}_{i}^{(I)}$ means the beam splitters of Bob $I$’s measurement. ${\text{D}}_{i}^{(I)}$ is Bob $I$’s $i$-th on-off detector, PS is *π*–phase shifter and $D$ is beam splitter, which is mathematically expressed as a displacement operator^42^.

In this subsection, we propose a method to implement the generalized sequential state discrimination of two coherent states. In our previous work^34^, the sequential state discrimination of two coherent states was discussed in the case of two receivers. Here, we deal with sequential state discrimination involving $N$ receivers. Based on the Banaszek model, Bob $I\in \{1,\ldots ,N\}$’s measurement can be designed as shown in Fig. 7. According to the figure, Alice prepares one out of two coherent states $|{\beta }_{i}\rangle (i=1,2)$, where $|{\beta }_{i}\rangle ={e}^{-|{\beta }_{i}{|}^{2}}\,{\sum }_{n=0}^{\infty }\,\frac{{\beta }_{i}^{n}}{\sqrt{n!}}|n\rangle$. Bob $I$’s measurement consists of beam combiners and two on-off detectors. If two on-off detectors ${\text{D}}_{1}^{(I)}$ and ${\text{D}}_{2}^{(I)}$ give outcomes of [off,on]([on,off]), Bob $I$ distinguishes Alice’s coherent state as $|{\beta }_{1}\rangle$($|{\beta }_{2}\rangle$). If two outcomes are [off,off], Bob $I$ cannot distinguish Alice’s coherent state. Here, [off,off] corresponds to an inconclusive result.

If $I < N$, beam splitters ${\text{BS}}_{1}^{(I)},{\text{BS}}_{2}^{(I)}$, and ${\text{BS}}_{3}^{(I)}$ are used to produce the post-measurement state. If ${R}_{i}^{(I)}$ denotes the reflectivity of ${\text{BS}}_{i}^{(I)}$, ${R}_{3}^{(I)}$ can be given as^34^

$${R}_{3}^{(I)}=\frac{{R}_{2}^{(I)}(1-{R}_{0}^{(I)})}{{R}_{1}^{(I)}{R}_{0}^{(I)}+{R}_{2}^{(I)}(1-{R}_{0}^{(I)})}.$$

Bob $I$’s post-measurement state can be expressed as $|\sqrt{{\prod }_{J=1}^{I}\,f({R}_{0}^{(J)},{R}_{1}^{(J)},{R}_{2}^{(J)})}{\beta }_{i}\rangle$, where $f$ is a real function: $f(x,y,z)=xy+(1-x)z$. According to Rule 1, Bob $N$ should perform an optimal unambiguous discrimination on Bob $N-1$’s post-measurement state. Therefore, Bob $N$’s measurement is same as in the Banaszek model (see Fig. 7). In our optical model, the success probability of the generalized sequential state discrimination is obtained as

32 $$\begin{array}{rcl}{P}_{s}^{({B}_{1},\ldots ,{B}_{N})} & = & {q}_{1}\{1-{e}^{-{\bar{R}}_{2}^{(1)}{\bar{R}}_{0}^{(1)}|{\beta }_{1}-{\beta }_{2}{|}^{2}}\}\{1-{e}^{-{\bar{R}}_{2}^{(2)}{\bar{R}}_{0}^{(2)}{K}^{(1)}|{\beta }_{1}-{\beta }_{2}{|}^{2}}\}\\  &  & \times \,\{1-{e}^{-{\bar{R}}_{2}^{(3)}{\bar{R}}_{0}^{(3)}{K}^{(2)}|{\beta }_{1}-{\beta }_{2}{|}^{2}}\}\times \cdots \times \{1-{e}^{-{\bar{R}}_{2}^{(N-1)}{\bar{R}}_{0}^{(N-1)}{K}^{(N-1)}|{\beta }_{1}-{\beta }_{2}{|}^{2}}\}\\  &  & \times \,\{1-{e}^{-{\bar{R}}^{(N)}{\kappa }^{(N)}|{\beta }_{1}-{\beta }_{2}{|}^{2}}\}\\  &  & +\,{q}_{2}\{1-{e}^{-{\bar{R}}_{1}^{(1)}{R}_{0}^{(1)}|{\beta }_{1}-{\beta }_{2}{|}^{2}}\}\{1-{e}^{-{\bar{R}}_{1}^{(2)}{R}_{0}^{(2)}{K}^{(1)}|{\beta }_{1}-{\beta }_{2}{|}^{2}}\}\\  &  & \times \,\{1-{e}^{-{\bar{R}}_{1}^{(3)}{R}_{0}^{(3)}{K}^{(2)}|{\beta }_{1}-{\beta }_{2}{|}^{2}}\}\times \cdots \times \{1-{e}^{-{\bar{R}}_{1}^{(N-1)}{R}_{0}^{(N-1)}{K}^{(N-1)}|{\beta }_{1}-{\beta }_{2}{|}^{2}}\}\\  &  & \times \,\{1-{e}^{-{R}^{(N)}{K}^{(N)}|{\beta }_{1}-{\beta }_{2}{|}^{2}}\}.\end{array}$$

**Figure 8:**
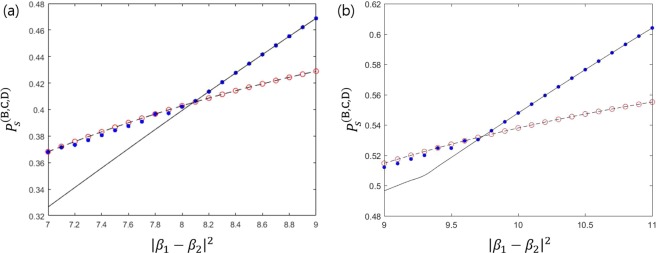
Plots of the success probability of the sequential state discrimination of coherent states, when there are three receivers. The solid black line (dashed black line) shows the optimal success probability when the three receivers discriminate two pure states of Alice (one out of two pure states of Alice). The red circles (blue dots) shows the maximum of Eq. (32), with (without) ${R}_{0}^{(I)}=1$. In (**a**), we use ${q}_{1}={q}_{2}=0.5$, and in (**b**) we use ${q}_{1}=0.4,{q}_{2}=0.6$.

**Figure 9:**
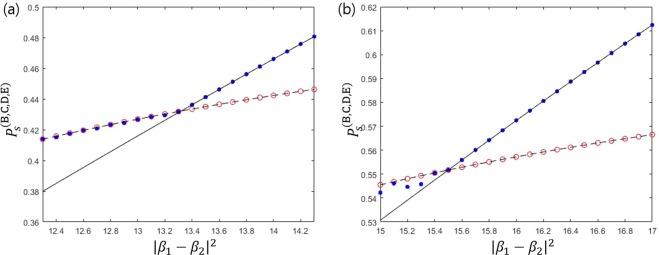
Plots of the success probability of the sequential state discrimination of the coherent states, when four receivers exist. The solid black line (dashed black line) shows the optimal success probability when three receivers discriminate two pure states of Alice (one out of two pure states of Alice). In (**a**), we use ${q}_{1}={q}_{2}=0.5$ and in (**b**), we use ${q}_{1}=0.4,{q}_{2}=0.6$.

Here, $\bar{R}=1-R$ and ${K}^{(I)}={\prod }_{J=1}^{I}\,f({R}_{0}^{(I)},{R}_{1}^{(I)},{R}_{2}^{(I)})$. The optimal success probability of Eq. (32) is shown in Figs. 8 and 9. In Fig. 8, we assume that the prior probabilities are equal. In Fig. 9, we assume that ${q}_{1}=0.4$ and ${q}_{2}=0.6$. The red circles (blue dots) shows the optimal success probability, with (without) the additional constraints ${R}_{0}^{(I)}=1(\,\forall \,I\in \{1,\ldots ,N-1\})$ and ${R}^{(N)}=1$. In Fig. 8, when $|{\beta }_{1}-{\beta }_{2}{|}^{2} < B(N)$, the maximum of Eq. (32) is equal to ${P}_{s}^{({B}_{1},\ldots ,{B}_{N}),\text{opt}}={(1-|\langle {\beta }_{1}|{\beta }_{2}\rangle {|}^{1/N})}^{N}$. When $|{\beta }_{1}-{\beta }_{2}{|}^{2}\ge B(N)$, the maximum of Eq. (32) is equal to ${P}_{s}^{({B}_{1},\ldots ,{B}_{N}),\text{opt}}=\frac{1}{2}{(1-|\langle {\beta }_{1}|{\beta }_{2}\rangle {|}^{2/N})}^{N}$, where $B(N)=-\,2N\,\text{ln}({2}^{1/N}-1)$. We can numerically calculate $B(N)$ as

$$\begin{array}{c}B(2)=3.525494348078171\\ B(3)=8.084264089976305\\ B(4)=13.319304138790477\\ B(5)=19.058354881391878\\ B(6)=25.199449280612455\\ B(7)=31.675056582901121\end{array}$$

In Fig. 9, when $|{\beta }_{1}-{\beta }_{2}{|}^{2}$ is small, the maximum of Eq. (32) is equal to that of the optimization problem in Eq. (27). If $|{\beta }_{1}-{\beta }_{2}{|}^{2}$ is large, the maximum of Eq. (32) is equal to ${P}_{s}^{({B}_{1},\ldots ,{B}_{N}),\text{opt}}=0.6{(1-|\langle {\beta }_{1}|{\beta }_{2}\rangle {|}^{2/N})}^{N}$. Therefore, we conclude that our model can optimally perform a generalized sequential state discrimination of two coherent states. Because the Huttner model provides the same measurement probability distribution as that of the Banaszek model^34^, generalized sequential state discrimination can be performed optimally, using the modified Huttner model. Unlike the Banaszek model, the Huttner model uses a horizontally polarized coherent state $|{\beta }_{i}\rangle$, mixed with a vertically polarized coherent state $|\gamma \rangle$, as an information carrier. Therefore, if an eavesdropper attempts to steal information encoded in coherent light, the eavesdropper will inevitably change at least one of two polarized coherent states. Hence, eavesdropping ruins the unambiguous discrimination and produces an error on the receiver’s measurement. Therefore, all receivers can notice the fact that an eavesdropper exists by checking whether an error occurs.

**Figure 10:**
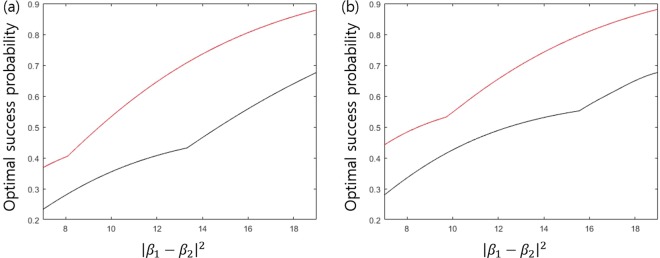
The optimal success probabilities when receivers are three and four. In (**a**), the prior probabilities of two coherent states are identical. In (**b**), the prior probabilities of two coherent states are ${q}_{1}=0.4$ and ${q}_{1}=0.6$. Here, the solid red (black) line denotes the case of $N=3$($N=4$). This result shows that the optimal success probability of $N=3$ is larger than that of $N=4$. It can be understood as follows: For instance, when receivers are three and four, the third receiver called David should use a strategy depending on the existence of an extra receiver. In the case of $N=3$, David is the last receiver and should choose optimal unambiguous discrimination. However, in the case of $N=4$, David is not the last receiver and should use nonoptimal unambiguous discrimination. Therefore, the optimal success probability of $N=3$ is larger than that of $N=4$.

We compare the optimal success probabilities of Figs. 8 and 9. One can see that in Fig. 10, the solid red line which denotes the optimal success probability of sequential state discrimination for three receivers is larger than the solid black line which denotes the optimal success probability of sequential state discrimination for four receivers. The reason for the difference between two success probabilities is due to the strategy of David. In the case of $N=3$, David becomes the last receiver and chooses optimal unambiguous discrimination. However, in the case of $N=4$, David is not the last receiver and should choose nonoptimal unambiguous discrimination.

#### Implementing the sequential state discrimination of two mixed states

**Figure 11:**
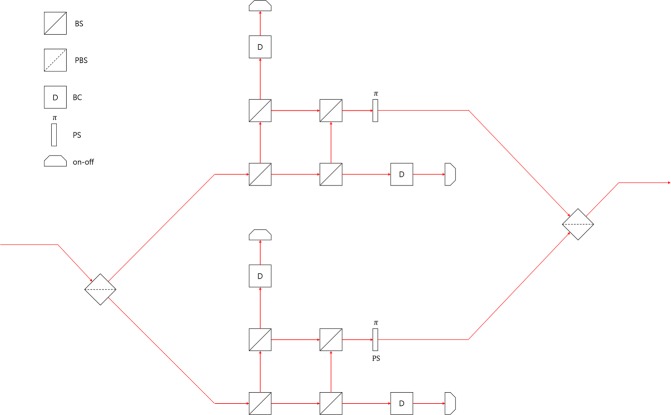
Schematic of the optical model for performing the generalized sequential state discrimination of two mixed states. Here, BS is a beam splitter, PBS is a polarized beam splitter, D is a beam combiner, PS is a phase shifter, and on-off is an on-off detector.

In this subsection, we propose a way to implement the generalized sequential state discrimination of two mixed states. In our model, the mixed states are produced as in the following process (See Fig. 11): First, Alice prepares one out of two coherent states $$|{\beta }_{i}\rangle ,|{\bar{\beta }}_{i}\rangle $$, with prior probabilities $$|{r}_{i}\rangle ,|{\bar{r}}_{i}\rangle $$. Second, Alice polarizes the coherent state $$|{\beta }_{i}\rangle $$$$(|{\bar{\beta }}_{i}\rangle )$$ in the horizontal (vertical) direction. After performing two steps, Alice obtains a mixed state as^50^

33 $${\rho }_{i}={r}_{i}|{\beta }_{i}\otimes H\rangle \langle {\beta }_{i}\otimes H|+{\bar{r}}_{i}|{\bar{\beta }}_{i}\otimes V\rangle \langle {\bar{\beta }}_{i}\otimes V|,$$

where $|{\beta }_{i}\otimes H\rangle =|{\beta }_{i}\rangle \otimes |H\rangle$ and $|{\bar{\beta }}_{i}\otimes V\rangle =|{\bar{\beta }}_{i}\rangle \otimes |V\rangle$. Because $|{\beta }_{i}\otimes H\rangle$ and $|{\bar{\beta }}_{i}\otimes V\rangle$ are orthogonal to each other, Eq. (33) is equal to Eq. (6). If Alice wants to build rank-$m$ mixed states, she would perform the following process: First, Alice prepares a coherent state $|{\beta }_{ij}\rangle \in \{|{\beta }_{i1}\rangle ,\ldots ,|{\beta }_{im}\rangle \}$ with a prior probability ${r}_{ij}$. Second, she passes ${\beta }_{ij}$ to the *j*-th photon path (${D}_{j}$). Then, she obtains the following mixed states:

34 $${\rho }_{i}={r}_{i1}|{\beta }_{i1}\otimes {D}_{1}\rangle \langle {\beta }_{i1}\otimes {D}_{1}|+\cdots +{r}_{im}|{\beta }_{im}\otimes {D}_{m}\rangle \langle {\beta }_{im}\otimes {D}_{m}|,$$

where $|{\beta }_{ij}\otimes {D}_{j}\rangle =|{\beta }_{ij}\rangle \otimes |{D}_{j}\rangle$. Moreover, $|{D}_{j}\rangle$ denotes the *j*-th path state.

$|{\beta }_{i}\otimes H\rangle$ and $|{\bar{\beta }}_{i}\otimes V\rangle$ can be perfectly discriminated by using a polarized beam splitter. We can use the Banaszek model to discriminate the nonorthogonal coherent states and in Fig. 10, we propose an optical model to discriminate the mixed states, expressed as Eq. (33). The Huttner model can also be used to perform the measurement for the generalized sequential state discrimination of two mixed states, in a similar way to that in Fig. 10.

## Security analysis of multiparty QKD based on sequential state discrimination - Part I: Eve’s single trial for eavesdropping

**Figure 12:**
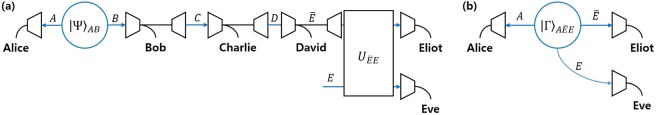
The case where Eve eavesdrops between David and Eliot. In (**a**), Eve eavesdrops between David and Eliot. Here, Eve interacts her system $E$ with system $\bar{E}$. The interaction is described by the global unitary operator ${U}_{\bar{E}E}$. After the interaction, Eve measures system $E$. The description of (**a**) is equivalent to the case of (**b**), where Alice, Eve, and Eliot share $|\varGamma {\rangle }_{A\bar{E}E}$.

In this section, we analyze the security of multiparty QKD, which optimal sequential state discrimination provides. Even though our analysis is confined to the case of four receivers (Bob, Charlie, David, Eliot), it can be consistently extended to the case of arbitrary number of receivers (See Fig. 12). In information theory, the classical bit of Alice can be expressed by $i\in \{0,1\}$ and we use $\{|0\rangle ,|1\rangle ,|?\rangle \}$ as a computational basis. Here, $|?\rangle$ is a computational basis that corresponds to the “failure” of Eve.

In QKD, Alice should minimize the prior information of the classical bit. Otherwise, Eve can obtain the prior information of the classical bit without being caught by the sender and the receiver. Alice prepares quantum states $|{\psi }_{0}\rangle$ and $|{\psi }_{1}\rangle$ corresponding to classical bit 0 and 1, with identical prior probability. In the view of Alice and Bob, it is the situation where Alice and Bob share the entangled state $|0\rangle \otimes |{\psi }_{0}\rangle +|1\rangle \otimes |{\psi }_{1}\rangle$^43^. Suppose that Eve tries to eavesdrop between Alice and Bob (Later, we will consider the case where the strategy of Eve is a collective attack^43^). When we denote the eavesdropping of Eve as a quantum channel ${\Lambda }_{B}^{(A\to B)}$, the bipartite quantum state between Alice and Bob can be expressed by

$${\sigma }_{AB}=({\text{id}}_{A}\otimes {\Lambda }_{B}^{(A\to B)})(|\Psi \rangle \langle \Psi {|}_{AB}),$$

where $|\Psi {\rangle }_{AB}=(1/\sqrt{2}){(|0\rangle }_{A}\otimes |{\psi }_{0}{\rangle }_{B}+|1{\rangle }_{A}\otimes |{\psi }_{1}{\rangle }_{B})$ and id_*A*_ is an identity channel. One can assume that Alice and Bob do not have any information about Eve. Then, ${\Lambda }_{B}^{(A\to B)}$ can be seen as

$${\Lambda }_{B}^{(A\to B)}(\sigma )={\eta }_{AB}\sigma +(1-{\eta }_{AB})\frac{{I}_{B}}{2},$$

where ${\eta }_{AB}\in [0,1]$ is the efficiency of the quantum channel. As the efficiency is more close to 1, Alice and Bob can be less affected by Eve. Then, the bipartite state ${\sigma }_{AB}$ can be given as

35 $${\sigma }_{AB}={\eta }_{AB}|\Psi \rangle \langle \Psi {|}_{AB}+(1-{\eta }_{AB})|+\rangle \langle +{|}_{A}\otimes \frac{{I}_{B}}{2}.$$

The purification of the bipartite state ${\sigma }_{AB}$ can be found as

36 $$|\Gamma {\rangle }_{ABE}=\sqrt{{\eta }_{AB}}|\Psi {\rangle }_{AB}\otimes |?{\rangle }_{E}+\sqrt{1-{\eta }_{AB}}|+{\rangle }_{A}\otimes |{\phi }_{+}{\rangle }_{BE},$$

where $|{\phi }_{+}\rangle =(1/\sqrt{2})(|00\rangle +|11\rangle )$. Equation (36) can be understood as follows. If Eve fails to eavesdrop with a probability ${\eta }_{AB}$, Alice and Bob succeed in sharing $|\Psi \rangle$. Then, Alice and Bob can share a secret key. Meanwhile, the quantum state of Eve is given by $|?\rangle$. If Eve succeeds to eavesdrop with a probability of $1-{\eta }_{AB}$, Eve shares a maximally entangled state with Bob.

The joint probability of the case where Alice prepares $|{\psi }_{i}\rangle$, Bob obtains $j$ as a result of measurement, and Eve gets bit $k$ can be given as follows:

37 $${P}_{ABE}(i,j,k)={\text{Tr}}_{ABE}\{|\Gamma \rangle \langle \Gamma {|}_{ABE}(|i\rangle \langle i{|}_{A}\otimes {M}_{j}\otimes |k\rangle \langle k{|}_{E})\},\,i,j\in \{0,1\}.$$

When the prior probability is identical, the optimal measurement of Bob corresponds to the case of ${\alpha }_{0}={\alpha }_{1}=1-{s}^{1/N}$. Therefore, one can obtain the following probabilities (The detailed derivation can be found in Method):

38 $$\begin{array}{rcl}{P}_{A}(i) & = & \frac{1}{2},\\ {P}_{AB}(i,j) & = & {\eta }_{AB}\frac{1}{2}(1-{s}^{1/4}){\delta }_{ij}+(1-{\eta }_{AB})\frac{1-{s}^{1/4}}{4(1-{s}^{2})},\\ {P}_{E}(k) & = & \frac{1-{\eta }_{AB}}{2},\\ {P}_{BE}(j,k) & = & \frac{(1-{\eta }_{AB})(1-{s}^{1/4})}{4\{1+{(-1)}^{k}s\}}.\end{array}$$

In Eq. (38), $i,j,k\in \{0,1\}$ is considered. Because an inconclusive result of Bob and failure of Eve cannot provide any information, Bob (Eve) can discard the inconclusive result (failure). The post-processing transforms four probabilities of Eq. (38) as follows:

$$\begin{array}{l}{\tilde{P}}_{A}(i)=\frac{{P}_{A}(i)}{{P}_{A}(0)+{P}_{A}(1)},{\tilde{P}}_{E}(i)=\frac{{P}_{E}(i)}{{P}_{E}(0)+{P}_{E}(1)},\\ {\tilde{P}}_{AB}(i,j)=\frac{{P}_{AB}(i,j)}{{P}_{AB}(0,0)+{P}_{AB}(0,1)+{P}_{AB}(1,0)+{P}_{AB}(1,1)},{\tilde{P}}_{BE}(j,k)=\frac{{P}_{BE}(j,k)}{{P}_{BE}(0,0)+{P}_{BE}(0,1)+{P}_{BE}(1,0)+{P}_{BE}(1,1)},\end{array}$$

for $i,j,k\in \{0,1\}$. It should be noted that the four probabilities obtained from post-processing are dependent only on the probability of the conclusive result. From these four probabilities, one can evaluate the secret key rate between Alice and Bob in the following way^44^:

$${K}_{AB:E}=\text{max}\{0,I(A:B)-I(B:E)\}.$$

Here, $I(X:Y)$($H(X,Y)$) is Shannon’s mutual information (joint Shannon entropy) between $X$ and $Y$. And because of ${\tilde{P}}_{A}(i)={\tilde{P}}_{E}(i)=1/2(\forall i\in \{0,1\})$, the relation of $I(A:B)-I(B:E)=H(B,E)-H(A,B)$ holds. Therefore, the secret key rate is rewritten as

$${K}_{AB:E}=\text{max}\,\{0,H(B,E)-H(A,B)\}.$$

Now, let us consider the case where Eve eavesdrops between Bob and Charlie. In this case, the quantum state between Alice and Bob is the entangled state $|0\rangle \otimes |{\phi }_{0}^{(B)}\rangle +|1\rangle \otimes |{\phi }_{1}^{(B)}\rangle$(The detailed derivation is found in Method). Here, $|{\phi }_{i}^{(B)}\rangle$ is the post-measurement quantum state corresponding to $i\in \{0,1\}$ which is the result of measurement of Bob. Because Eve eavesdrops between Bob and Charlie, when we denote the eavesdropping of Eve as a quantum channel ${\varLambda }_{B}^{(B\to C)}$, the bipartite state between Bob and Charlie can be given as

$${\sigma }_{AC}=({\text{id}}_{A}\otimes {\varLambda }_{C}^{(B\to C)})(|{\varPhi }^{(B)}\rangle \langle {\varPhi }^{(B)}{|}_{AC}),$$

where $|{\varPhi }^{(B)}{\rangle }_{AC}=(1/\sqrt{2}){(|0\rangle }_{A}\otimes |{\phi }_{0}^{(B)}{\rangle }_{C}+|1{\rangle }_{A}\otimes |{\phi }_{1}^{(B)}{\rangle }_{C})$. Likewise Eq. (37), we can obtain the marginal probabilities of Alice, Charlie, and Eve:

39 $$\begin{array}{rcl}{P}_{A}(i) & = & \frac{1}{2},\\ {P}_{AC}(i,j) & = & {\eta }_{BC}\frac{1}{2}(1-{s}^{1/4}){\delta }_{ij}+(1-{\eta }_{BC})\frac{1-{s}^{1/4}}{4(1-{s}^{3/2})},\\ {P}_{E}(k) & = & \frac{1-{\eta }_{BC}}{2},\\ {P}_{CE}(j,k) & = & \frac{(1-{\eta }_{BC})(1-{s}^{1/4})}{4\{1+{(-1)}^{k}{s}^{3/4}\}}.\end{array}$$

Here, ${\eta }_{BC}$ is a channel efficiency between Bob and Charlie. In the case where Eve eavesdrops between Charlie and David, the marginal probabilities of Alice, David, and Eve are given as

40 $$\begin{array}{rcl}{P}_{A}(i) & = & \frac{1}{2},\\ {P}_{AD}(i,j) & = & {\eta }_{CD}\frac{1}{2}(1-{s}^{1/4}){\delta }_{ij}+(1-{\eta }_{CD})\frac{1-{s}^{1/4}}{4(1-s)},\\ {P}_{E}(k) & = & \frac{1-{\eta }_{CD}}{2},\\ {P}_{DE}(j,k) & = & \frac{(1-{\eta }_{CD})(1-{s}^{1/4})}{4\{1+{(-1)}^{k}{s}^{1/2}\}},\end{array}$$

where ${\eta }_{BC}$ is a channel efficiency between Charlie and David. And in the case where Eve eavesdrops between David and Eliot, the marginal probabilities of Alice, Eliot, and Eve are obtained as (Here, the index $\bar{E}$ denotes Eliot)

41 $$\begin{array}{rcl}{P}_{A}(i) & = & \frac{1}{2},\\ {P}_{A\bar{E}}(i,j) & = & {\eta }_{D\bar{E}}\frac{1}{2}(1-{s}^{1/4}){\delta }_{ij}+(1-{\eta }_{D\bar{E}})\frac{1-{s}^{1/4}}{4(1-{s}^{1/2})},\\ {P}_{E}(k) & = & \frac{1-{\eta }_{D\bar{E}}}{2},\\ {P}_{\bar{E}E}(j,k) & = & \frac{(1-{\eta }_{D\bar{E}})(1-{s}^{1/4})}{4\{1+{(-1)}^{k}{s}^{1/4}\}}.\end{array}$$

The secret key rate can be evaluated by

$${K}_{AX:E}=\text{max}\{0,I(A:X)-I(X:E)\}=\text{max}\{0,H(X,E)-H(A,X)\},\,X\in \{B,C,D,\bar{E}\}.$$

**Figure 13:**
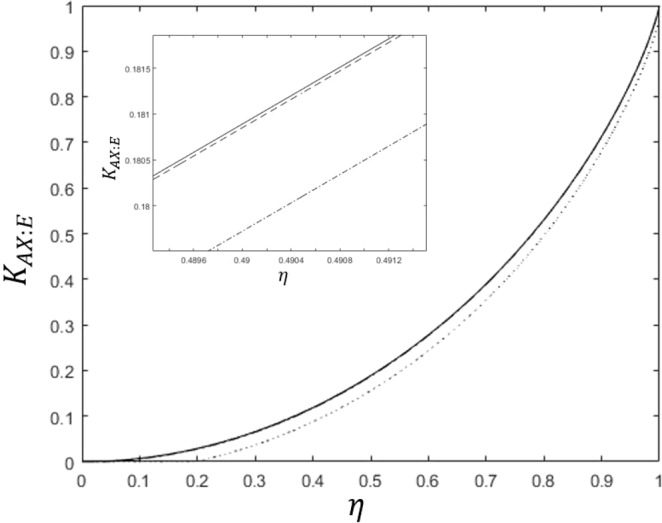
The secret key rate ${K}_{AX:E}$. Here, solid line, dashed line, dashed-dot line and dotted line correspond to the case of $X=B,C,D$ and $\bar{E}$, respectively. $\eta$ is the efficiency of the quantum channel, where eavesdropper Eve exists. According to this, the nonzero secret key rate is guaranteed in almost every region of $\eta$.

Figure 13 shows ${K}_{AX:E}$. Here, $\eta$ is the efficiency of channel where Eve involves. In Fig. 13, we consider the case of $s=0.00128$. Also, solid line, dashed line, dash-dot line, and dotted line denote the cases of $X=B,C,D$ and $\bar{E}$ respectively.

One can see that in Fig. 13, the secret key rate is the lowest in the case of $X=\bar{E}$(dotted line).This implies that the best performance of Eve can be obtained between David and Eliot. However, it should be emphasized that the effect depending on the position of eavesdropping is not big.

## Security analysis of multiparty QKD based on sequential state discrimination - Part II: Eve’s multi-trial for eavesdropping

Here, we consider the case where Eve uses quantum memories. By using quantum memories of Eve, she can perform eavesdropping between sender and receivers. Suppose that Alice, Bob, and Charlie are involved in sequential state discrimination as a sender and two receivers. In this case, for eavesdropping, Eve uses two quantum memories: one quantum memory is used between Alice and Bob, and another quantum memory is used between Bob and Charlie (It should be noted that even though we consider the sequential state discrimination comprised of a sender and two receivers, our argument can be extended to the sequential state discrimination comprised of a sender and multi-receivers).

**Figure 14:**
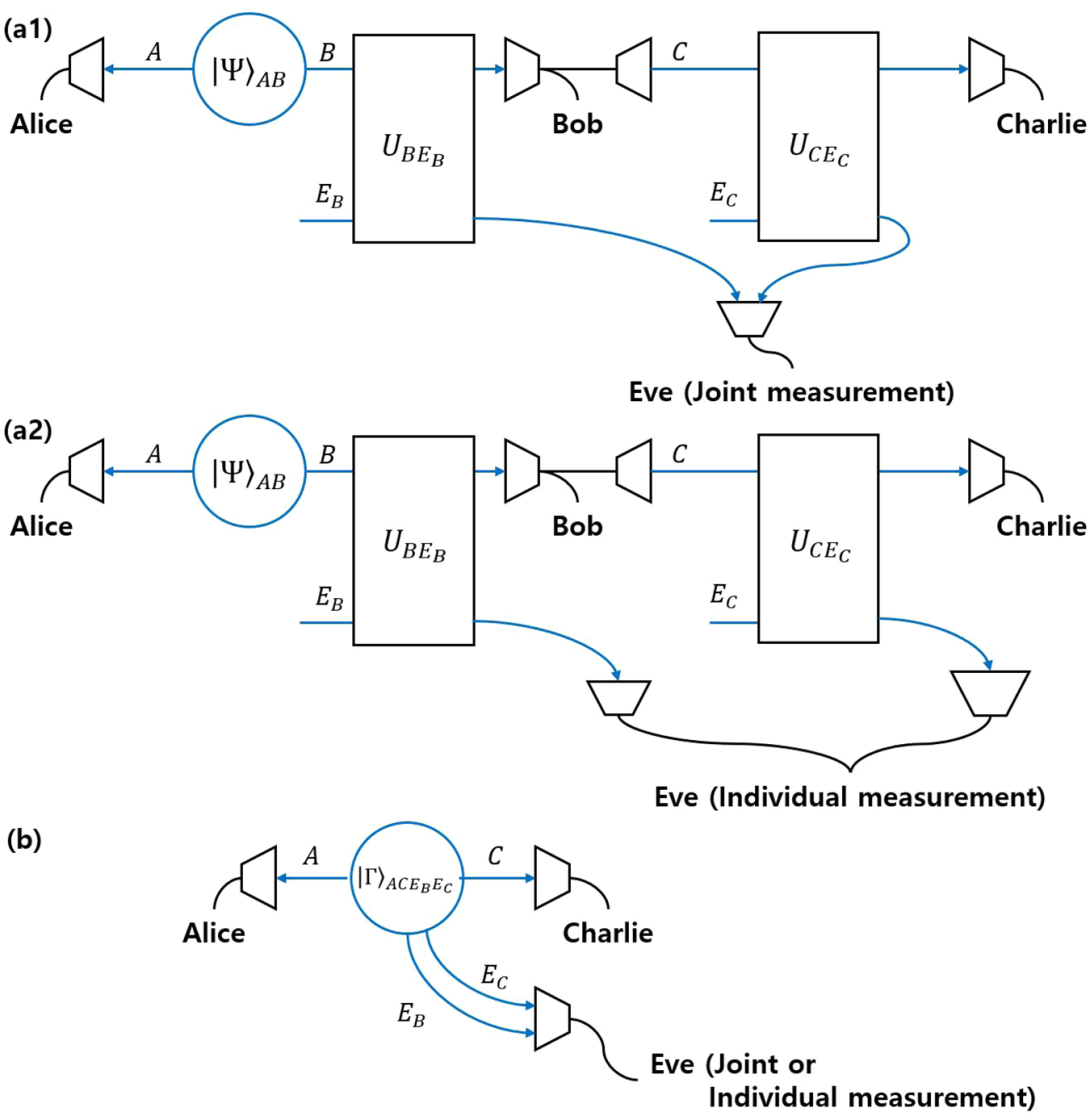
The case where Eve eavesdrops between Alice and Bob and between Bob and Charlie. In (**a1**), Eve eavesdrops between Alice and Bob and between Bob and Charlie. First, Eve prepares two systems of ${E}_{B}$ and ${E}_{C}$. Then, Eve interacts system ${E}_{B}$ with system $B$ (The interaction is expressed by the global unitary operator ${U}_{B{E}_{B}}$). Second, Eve interacts system ${E}_{C}$ with system $C$ (This interaction is described by the global unitary operator ${U}_{C{E}_{C}}$). Atfer these interactions, Eve measures system ${E}_{B}$ and ${E}_{C}$ globally (We denote this measurement as joint measurement). Here, the structure of joint measurement is determined by a unitary transformation $V={\{{V}_{pq}\}}_{p,q=1}^{18}$. If the unitary transformation $V$ is fixed as the identity, (**a1**) and (**a2**) are equivalent. In (**a2**), Eve measures system ${E}_{B}$ and system ${E}_{C}$ locally (We denote this measurement as an individual measurement). The description of (**a1**) and (**a2**) is equivalent to the case of (**b**), where Alice, Charlie, and Eve share $|\Gamma {\rangle }_{AC{E}_{B}{E}_{C}}$.

Now, if Eve use a quantum memory ${E}_{B}$ for evesdropping between Alice and Bob (see Fig. 14), system of $A,B$, and ${E}_{B}$ can be described as

$$|\Gamma {\rangle }_{AB{E}_{B}}=\sqrt{{\eta }_{AB}}|\Psi {\rangle }_{AB}\otimes |?{\rangle }_{{E}_{B}}+\sqrt{1-{\eta }_{AB}}|+{\rangle }_{A}\otimes |{\phi }_{+}{\rangle }_{B{E}_{B}}.$$

When Bob discards an inconclusive result, $|\Gamma {\rangle }_{AB{E}_{B}}$ becomes the following mixed state:

$${\sigma }_{AC{E}_{B}}={\mathscr{N}}({\eta }_{AB})\{|{ {\mathcal E} }_{0}\rangle \langle { {\mathcal E} }_{0}{|}_{A{E}_{B}}\otimes |{\phi }_{0}^{(B)}\rangle \langle {\phi }_{0}^{(B)}{|}_{C}+|{ {\mathcal E} }_{1}\rangle \langle { {\mathcal E} }_{1}{|}_{A{E}_{B}}\otimes |{\phi }_{1}^{(B)}\rangle \langle {\phi }_{1}^{(B)}{|}_{C}\}.$$

Here, ${K}_{i}^{(B)}:{{\mathscr{H}}}_{B}\to {{\mathscr{H}}}_{C}$ is the Kraus operator of Bob which corresponds to the measurement result $i\in \{0,1,?\}$. And we have ${\mathscr{N}}({\eta }_{AB})={[\langle { {\mathcal E} }_{0}|{ {\mathcal E} }_{0}\rangle +\langle { {\mathcal E} }_{1}|{ {\mathcal E} }_{1}\rangle ]}^{-1}$. The non- normalized vector $|{ {\mathcal E} }_{0}\rangle ,|{ {\mathcal E} }_{1}\rangle$ is defined as follows:

$$\begin{array}{rcl}|{ {\mathcal E} }_{0}{\rangle }_{A{E}_{B}} & = & \sqrt{\frac{{\eta }_{AB}(1-\sqrt{s})}{2}}|0{\rangle }_{A}\otimes |?{\rangle }_{{E}_{B}}+\sqrt{\frac{(1-{\eta }_{AB})(1-\sqrt{s})}{2}}|+{\rangle }_{A}\otimes |{\alpha }_{0}{\rangle }_{{E}_{B}},\\ |{ {\mathcal E} }_{1}{\rangle }_{A{E}_{B}} & = & \sqrt{\frac{{\eta }_{AB}(1-\sqrt{s})}{2}}|1{\rangle }_{A}\otimes |?{\rangle }_{{E}_{B}}+\sqrt{\frac{(1-{\eta }_{AB})(1-\sqrt{s})}{2}}|+{\rangle }_{A}\otimes |{\alpha }_{1}{\rangle }_{{E}_{B}}.\end{array}$$

Here, Bob’s POVM consists of ${\alpha }_{i}|{\alpha }_{i}\rangle \langle {\alpha }_{i}|$. When Eve uses a quantum memory ${E}_{C}$ for eavesdropping between Bob and Charlie, the eavesdropping of Eve can be expressed as a quantum channel ${\Lambda }_{C}^{(B\to C)}$. That is, the eavesdropping of Eve transforms ${\sigma }_{AC{E}_{B}}$ as follows:

$$\begin{array}{rcl}{\text{id}}_{{\rm{A}}{E}_{B}}\otimes {\Lambda }_{C}^{(B\to C)}({\sigma }_{{\rm{A}}C{E}_{B}}) & = & {\mathscr{N}}({\eta }_{AB})[{\eta }_{BC}\mathop{\sum }\limits_{x=0}^{1}|{ {\mathcal E} }_{x}\rangle \langle { {\mathcal E} }_{x}{|}_{A{E}_{B}}\otimes |{\phi }_{x}^{(B)}\rangle \langle {\phi }_{p}^{(B)}{|}_{C}\\  &  & +\,(1-{\eta }_{BC})\mathop{\sum }\limits_{x=0}^{1}|{ {\mathcal E} }_{x}\rangle \langle { {\mathcal E} }_{x}{|}_{A{E}_{B}}\otimes \frac{{I}_{C}}{2}].\end{array}$$

The purification of ${\text{id}}_{A{E}_{B}}\otimes {\Lambda }_{C}^{(B\to C)}({\sigma }_{AC{E}_{B}})$ is given as

$$\begin{array}{rcl}|\Gamma {\rangle }_{AC{E}_{B}{E}_{C}} & = & \sqrt{{\eta }_{BC}{\mathscr{N}}({\eta }_{AB})}{\{|{ {\mathcal E} }_{0}\rangle }_{A{E}_{B}}\otimes |{\phi }_{0}^{(B)}{\rangle }_{C}\otimes |{?}_{0}{\rangle }_{{E}_{C}}\\  &  & +\,|{ {\mathcal E} }_{1}{\rangle }_{A{E}_{B}}\otimes |{\phi }_{1}^{(B)}{\rangle }_{C}\otimes |{?}_{1}{\rangle }_{{E}_{C}}\}\\  & = & \sqrt{\frac{(1-{\eta }_{BC}){\mathscr{N}}({\eta }_{AB})}{2}}(|{ {\mathcal E} }_{0}{\rangle }_{A{E}_{B}}\otimes |0{\rangle }_{C}\otimes |00{\rangle }_{{E}_{C}}\\  &  & +\,|{ {\mathcal E} }_{1}{\rangle }_{A{E}_{B}}\otimes |0{\rangle }_{C}\otimes |01{\rangle }_{{E}_{C}}\\  &  & +\,|{ {\mathcal E} }_{0}{\rangle }_{A{E}_{B}}\otimes |1{\rangle }_{C}\otimes |10{\rangle }_{{E}_{C}}+|{ {\mathcal E} }_{1}{\rangle }_{A{E}_{B}}\otimes |1{\rangle }_{C}\otimes |11{\rangle }_{{E}_{C}}).\end{array}$$

Here, $|{?}_{0}\rangle$ and $|{?}_{1}\rangle$ are computational basis corresponding to Eve’s failure. It should be noted that $|{?}_{0}\rangle$ and $|{?}_{1}\rangle$ are orthogonal to each other. The computational basis is orthogonal to $|00\rangle ,|01\rangle ,|10\rangle$, and $|11\rangle$.

Unlike system ${E}_{B}$, system ${E}_{C}$ is composed of two subsystems. It is because when Eve eavesdrops between Bob and Charlie, Eve also can eavesdrop between Alice and Bob. When ${E}_{C1}$ and ${E}_{C2}$ are the subsystems of ${E}_{C}$, $|\Gamma {\rangle }_{AC{E}_{B}{E}_{C}}$ can be described in the following way:

$$\begin{array}{rcl}|\Gamma {\rangle }_{AC{E}_{B}{E}_{C}} & = & \sqrt{{\eta }_{BC}{\mathscr{N}}({\eta }_{AB})}\{|{ {\mathcal E} }_{0}{\rangle }_{A{E}_{B}}\otimes |{\phi }_{0}^{(B)}{\rangle }_{C}\otimes |{?}_{0}{\rangle }_{{E}_{C}}\\  &  & +\,|{ {\mathcal E} }_{1}{\rangle }_{A{E}_{B}}\otimes |{\phi }_{1}^{(B)}{\rangle }_{C}\otimes |{?}_{1}{\rangle }_{{E}_{C}}\}\\  &  & +\,\sqrt{(1-{\eta }_{BC}){\mathscr{N}}({\eta }_{AB})}\{|{ {\mathcal E} }_{0}{\rangle }_{A{E}_{B}}\otimes |0{\rangle }_{{E}_{C2}}\\  &  & +\,|{ {\mathcal E} }_{1}{\rangle }_{A{E}_{B}}\otimes |1{\rangle }_{{E}_{C2}}\}\otimes |{\phi }_{+}{\rangle }_{C{E}_{C1}}.\end{array}$$

Here, $|{\phi }_{+}\rangle$ is a maximally entangled state $|{\phi }_{+}\rangle =(|00\rangle +|11\rangle )/\sqrt{2}$. In other word, subsystem ${E}_{C1}$ is used for eavesdropping of Alice’s quantum state and subsystem ${E}_{C2}$ is used for eavesdropping of Bob’s post-measurement state.

When Bob and Charlie perform optimal sequential state discrimination, the prior probability is ${P}_{A}(i)=1/2(\forall i\in \{0,1\})$ (The detailed calculation can be found in Method). And, the marginal probability between Alice and Charlie is given as follows:

$$\begin{array}{rcl}{P}_{AC}(i,j) & = & {\eta }_{BC}{\mathscr{N}}({\eta }_{AB})(1-\sqrt{s}){\delta }_{j0}{{\mathscr{X}}}_{0}(i)+{\eta }_{BC}{\mathscr{N}}({\eta }_{AB})(1-\sqrt{s}){\delta }_{j1}{{\mathscr{X}}}_{1}(i)\\  &  & +\,\frac{(1-{\eta }_{BC}){\mathscr{N}}({\eta }_{AB})}{2(1+\sqrt{s})}\{{{\mathscr{X}}}_{0}(i)+{{\mathscr{X}}}_{1}(i)\}.\end{array}$$

Here, ${{\mathscr{X}}}_{a}(i)$ is defined in the following way:

$${{\mathscr{X}}}_{a}(i)=\frac{1}{2}{\eta }_{AB}(1-\sqrt{s}){\delta }_{ia}+\frac{(1-{\eta }_{AB})(1-\sqrt{s})}{4(1-{s}^{2})}.$$

**Figure 15:**
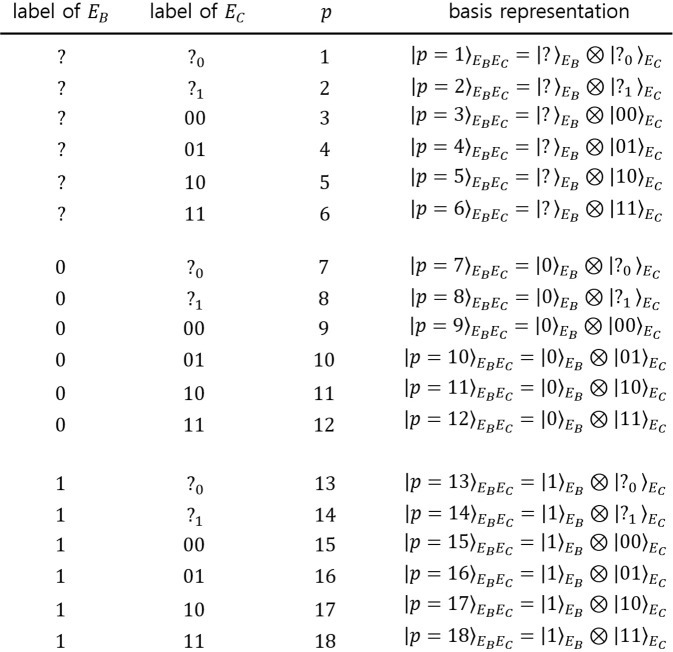
The label of $p$ in terms of basis of ${E}_{B}$ and ${E}_{C}$. As an example, $p=1$ corresponds to the case where the basis of ${E}_{B}$ is “?” and the basis of ${E}_{C}$ is “?_0_”, which is denoted as $|p=1{\rangle }_{{E}_{B}{E}_{C}}=|?{\rangle }_{{E}_{B}}\otimes |{?}_{0}{\rangle }_{{E}_{C}}$. And $p=2$ corresponds to the case where the basis of ${E}_{B}$ is “?” and the basis of ${E}_{C}$ is “?_1_”, which is denoted as $|p=2{\rangle }_{{E}_{B}{E}_{C}}=|?{\rangle }_{{E}_{B}}\otimes |{?}_{1}{\rangle }_{{E}_{C}}$.

The result of measurement of Eve can be expressed as a single label $p\in {\{?,0,1\}}_{{E}_{B}}\times {\{{?}_{0},{?}_{1},00,01,10,11\}}_{{E}_{C}}$ (The notation of the label can be found in Fig. 15). It should be noted that $|{?}_{0}\rangle$ and $|{?}_{1}\rangle$ cannot be expressed by a linear combination of $|00\rangle ,|01\rangle ,|10\rangle$, and $|11\rangle$.

The marginal probability of Charlie and Eve is given by

$$\begin{array}{rcl}{P}_{E}(p) & = & \langle \varGamma |({I}_{AC}\otimes |{\pi }_{p}{\rangle }_{{E}_{B}{E}_{C}}\langle {\pi }_{p}{|}_{{E}_{B}{E}_{C}})|\Gamma \rangle \\  & = & {\langle \Gamma {|}_{AC{E}_{B}{E}_{C}}({I}_{AC}\otimes |{\pi }_{p}{\rangle }_{{E}_{B}{E}_{C}})({I}_{AC}\otimes \langle {\pi }_{p}{|}_{{E}_{B}{E}_{C}})|\Gamma \rangle }_{AC{E}_{B}{E}_{C}},\\ {P}_{CE}(k,p) & = & \langle \Gamma |({I}_{A}\otimes {\beta }_{k}|{\beta }_{k}\rangle {\langle {\beta }_{k}{|}_{C}\otimes |{\pi }_{p}\rangle }_{{E}_{B}{E}_{C}}\langle {\pi }_{p}{|}_{{E}_{B}{E}_{C}})|\Gamma \rangle \\  & = & {\beta }_{k}\langle \Gamma {|}_{AC{E}_{B}{E}_{C}}({I}_{A}\otimes |{\beta }_{k}{\rangle }_{C}\otimes |{\pi }_{p}{\rangle }_{{E}_{B}{E}_{C}})\\  &  & \times \,({I}_{A}\otimes \langle {\beta }_{k}{|}_{C}\otimes \langle {\pi }_{p}{|}_{{E}_{B}{E}_{C}})|\Gamma {\rangle }_{AC{E}_{B}{E}_{C}}.\end{array}$$

${P}_{E}(p)$ and ${P}_{CE}(p)$ can be found in Method and labeled vector $|{\pi }_{p}{\rangle }_{{E}_{B}{E}_{C}}$ can be expressed as

$$|{\pi }_{p}{\rangle }_{{E}_{B}{E}_{C}}=\mathop{\sum }\limits_{q=1}^{18}{V}_{pq}|q{\rangle }_{{E}_{B}{E}_{C}},$$

where every ${V}_{pq}\in {\mathbb{C}}$ satisfies ${\sum }_{r=1}^{18}{V}_{pr}{V}_{qr}^{\ast }={\sum }_{k=r}^{18}{V}_{rp}^{\ast }{V}_{rq}={\delta }_{pq}$. The set of these labeled vectors $\{|{\pi }_{p}\rangle {\}}_{p=1}^{18}$ forms the joint projective measurement $\{|{\pi }_{p}\rangle {\langle {\pi }_{p}|\}}_{p=1}^{18}$ of Eve.

When Alice, Charlie, and Eve discard inconclusive result, marginal probability becomes

$$\begin{array}{rcl}{\tilde{P}}_{AC}(i,j) & = & \frac{{P}_{AC}(i,j)}{{\sum }_{i,j=0}^{1}{P}_{AC}(i,j)},\\ {\tilde{P}}_{E}(p) & = & \frac{{P}_{E}(p)}{{\sum }_{p\notin 1,2,3,4,5,6,7,8,13,14\}}{P}_{E}(p)},\\ {\tilde{P}}_{CE}(k,p) & = & \frac{{P}_{CE}(k,p)}{{\sum }_{k=0}^{1}{\sum }_{p\notin \{1,2,3,4,5,6,7,8,13,14\}}{P}_{CE}(k,p)}.\end{array}$$

**Figure 16:**
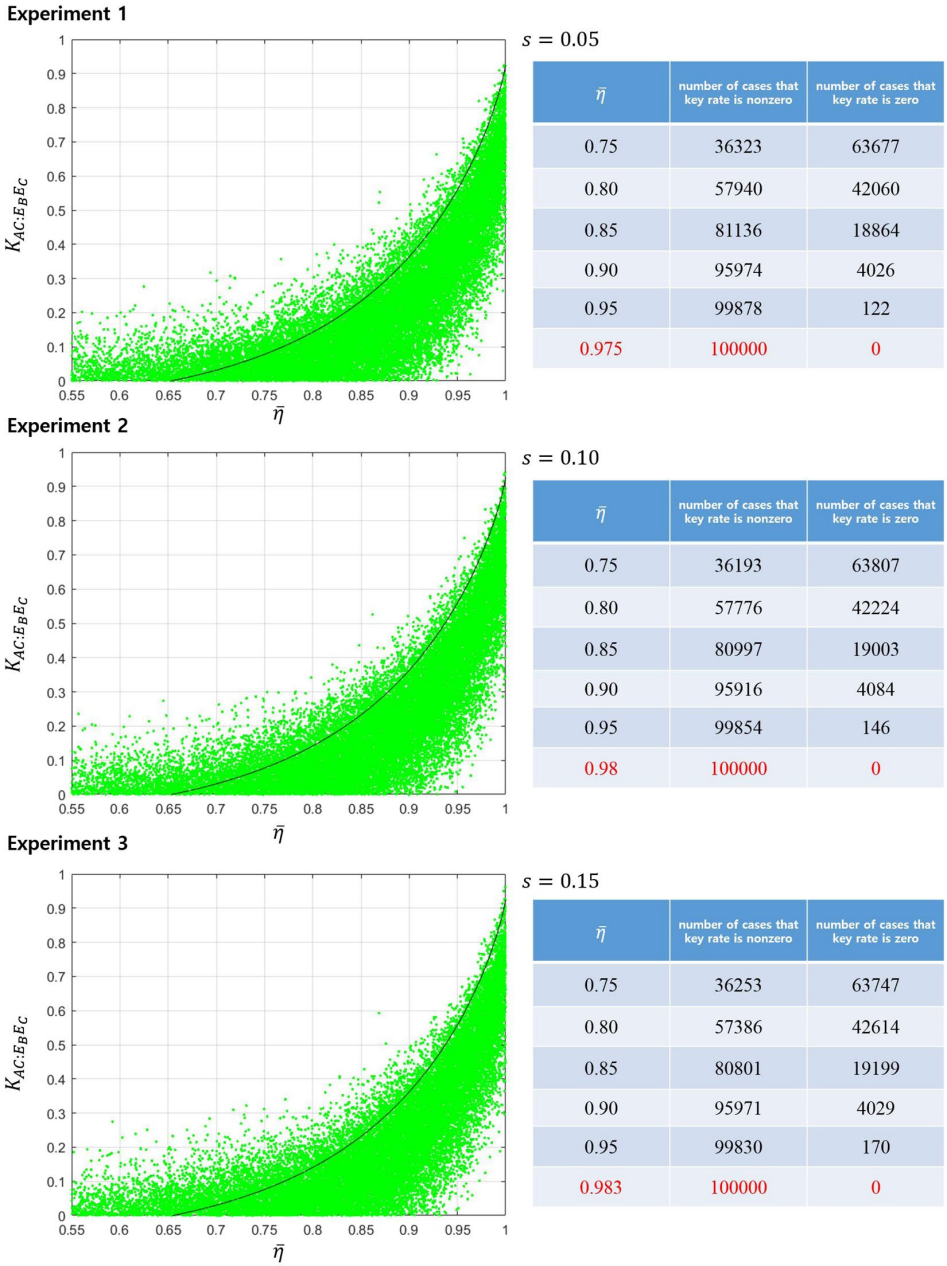
The secret key rate between Alice and Charlie when Eve eavesdrops between Alice and Bob and between Bob and Charlie. The solid black line denotes the case where the unitary transformation of Eve is the identity. The green dots display the secret key rate when the unitary transformation of Eve is arbitrary. The table shows the ratio of nonzero secret key rate out of 100000 random cases.

The secret key rate between Alice and Charlie, which is given by

$${K}_{AC:{E}_{B}{E}_{C}}=\text{max}\{0,I(A:C)-I(C:E)\}=\text{max}\{0,H(A)-H(C,A)+H(C,E)-H(E)\},$$

is displayed in Fig. 16. For convenience, it is assumed that each channel efficiency is equal to each other ${\eta }_{AB}={\eta }_{BC}=\bar{\eta }$. In Fig. 16, the solid black line corresponds to the case where Eve measures her subsystem, by the eighteen basis (This is the case where unitary transformation ${\{{V}_{pq}\}}_{p,q=1}^{18}$ is an identity). And, green points correspond to the cases of random unitary transformation. Because Alice and Bob cannot know Eve’s system, treating unitary transformation as a random one can be justified.

## Experiment 1: The nonzero secret key rate in the case of *s* = 0.05

### Individual measurement

When Eve measures her subsystem using eighteen basis $\{|p\rangle {\}}_{p=1}^{18}$, if $\bar{\eta }$ is greater than ${\bar{\eta }}_{crit}=0.65295$, Alice and Charlie can obtain nonzero secret key rate (The method of simulation can be found in Method).

### Arbitrary joint measurement

When Eve selects a measurement out of 100000 measurements, the ratio to obtaining nonzero secret key rate increases as $\bar{\eta }$ increases. When $\bar{\eta }=0.75$, the ratio to obtaining nonzero secret key rate becomes $\mathrm{36.323 \% }$. When $\bar{\eta }=0.95$, the ratio to obtaining nonzero secret key rate becomes $\mathrm{99.978 \% }$. Specially, when $\bar{\eta }=0.975$, nonzero secret key rate can be obtained regardless of a measurement.

## Experiment 2: The nonzero secret key rate in the case of *s* = 0.10

### Individual measurement

When Eve measures her subsystem using eighteen basis $\{|p\rangle {\}}_{p=1}^{18}$, if $\bar{\eta }$ is greater than ${\bar{\eta }}_{crit}=0.65364$, Alice and Charlie can obtain nonzero secret key rate (The method of simulation can be found in Method).

### Arbitrary joint measurement

When Eve selects a measurement out of 100000 measurements, the ratio to obtaining nonzero secret key rate increases as $\bar{\eta }$ increases. When $\bar{\eta }=0.75$, the ratio to obtaining nonzero secret key rate becomes $\mathrm{36.193 \% }$. When $\bar{\eta }=0.95$, the ratio to obtaining nonzero secret key rate becomes $\mathrm{99.854 \% }$. Specially, when $\bar{\eta }=0.98$, nonzero secret key rate can be obtained regardless of a measurement.

## Experiment 3: The nonzero secret key rate in the case of *s* = 0.15

### Individual measurement

When Eve measures her subsystem using eighteen basis $\{|p\rangle {\}}_{p=1}^{18}$, if $\bar{\eta }$ is greater than ${\bar{\eta }}_{crit}=0.65480$, Alice and Charlie can obtain nonzero secret key rate (The method of simulation can be found in Method).

### Arbitrary joint measurement

When Eve selects a measurement out of 100000 measurements, the ratio to obtaining nonzero secret key rate increases as $\bar{\eta }$ increases. When $\bar{\eta }=0.75$, the ratio to obtaining nonzero secret key rate becomes $\mathrm{36.253 \% }$. When $\bar{\eta }=0.95$, the ratio to obtaining nonzero secret key rate becomes $\mathrm{99.830 \% }$. Specially, when $\bar{\eta }=0.983$, nonzero secret key rate can be obtained regardless of a measurement.

## Discussion

In this report, we presented a generalization of sequential state discrimination. In our work, we did not limit the prior probabilities and the number of quantum states and receivers. We could express the generalized sequential state discrimination as a mathematical optimization problem. Because this optimization cannot be solved analytically, a numerical method was applied to the construction of the optimal POVM. Our optimization problems include all the results of the previous work^27^ as special cases. Moreover, we applied the generalized sequential state discrimination to multiparty QKD. If Alice prepares one out of two pure states, the generalized sequential state discrimination can be used to perform multiparty QKD when there are a few receivers. It should be noted that if Alice prepares one out of two mixed states, the optimal success probability of generalized sequential state discrimination can exceed that of the quantum reproducing and quantum broadcasting strategies. Therefore, the generalized sequential state discrimination of mixed states has more potential for application to multiparty QKD than the other strategies. Finally, we analyze the security of multiparty QKD provided by optimal sequential state discrimination. Our analysis shows that the multiparty QKD guarantees nonzero secret key rate even in low channel efficiency.

Even if we considered discriminating two quantum states, we could extend our argument for generalized sequential state discrimination to more than two quantum states. However, an unambiguous discrimination of more than three quantum states has not been known yet. Therefore, one needs to find a way to discriminate more than three quantum states, without any error.

If pure states $\{|{\psi }_{1}\rangle ,\ldots ,|{\psi }_{n}\rangle \}$ are linearly dependent, unambiguous discrimination cannot be performed. However, in the case of finite copies of coherent states ${\{|{\psi }_{1}\rangle }^{\otimes C},\ldots ,|{\psi }_{n}{\rangle }^{\otimes C}\}$, they are not always linearly dependent. Therefore, when finite copies of pure state are available, the receiver can perform unambiguous discrimination on ${\{|{\psi }_{1}\rangle }^{\otimes C},\ldots ,|{\psi }_{n}{\rangle }^{\otimes C}\}$^51,52^. Although supports of mixed states $\{{\rho }_{1},\ldots ,{\rho }_{n}\}$ are completely overlapped to each other, supports of $\{{\rho }_{1}^{\otimes C},\ldots ,{\rho }_{n}^{\otimes C}\}$ may not be completely overlapped and unambiguous discrimination of $\{{\rho }_{1}^{\otimes C},\ldots ,{\rho }_{n}^{\otimes C}\}$ can be performed. Using this idea, one may devise sequential state discrimination of general mixed states.

## Methods

### Derivation of the optimization problem

In this section, we derive the optimization problem of generalized sequential state discrimination. First, a tangential point $({\gamma }_{1},{\gamma }_{2})$ between a plane ${P}_{s}^{(\text{B},\text{C},\text{D})}$ and a surface $(1-{\gamma }_{1})(1-{\gamma }_{2})=|\langle {\phi }_{1}^{(\text{C})}|{\phi }_{2}^{(\text{C})}\rangle {|}^{2}$ satisfies the following equality:

$$\frac{\partial {P}_{s}^{(\text{B},\text{C},\text{D})}/\partial {\gamma }_{1}}{\partial {P}_{s}^{(\text{B},\text{C},\text{D})}/\partial {\gamma }_{2}}=\frac{\partial \{(1-{\gamma }_{1})(1-{\gamma }_{2})-|\langle {\phi }_{1}^{(\text{C})}|{\phi }_{2}^{(\text{C})}\rangle {|}^{2}\}/\partial {\gamma }_{1}}{\partial \{(1-{\gamma }_{1})(1-{\gamma }_{2})-|\langle {\phi }_{1}^{(\text{C})}|{\phi }_{2}^{(\text{C})}\rangle {|}^{2}\}/\partial {\gamma }_{2}}.$$

Combining both above equality and $(1-{\gamma }_{1})(1-{\gamma }_{2})=|\langle {\phi }_{1}^{(\text{C})}|{\phi }_{2}^{(\text{C})}\rangle {|}^{2}$, we obtain $({\gamma }_{1},{\gamma }_{2})$ as

$$\begin{array}{rcl}{\gamma }_{1} & = & 1-|\langle {\phi }_{1}^{(\text{C})}|{\phi }_{2}^{(\text{C})}\rangle |\sqrt{\frac{{q}_{2}{\alpha }_{2}{\beta }_{2}}{{q}_{1}{\alpha }_{1}{\beta }_{1}}}=1-\frac{|\langle {\phi }_{1}^{(\text{B})}|{\phi }_{2}^{(\text{B})}\rangle |}{\sqrt{(1-{\beta }_{1})(1-{\beta }_{2})}}\sqrt{\frac{{q}_{2}{\alpha }_{2}{\beta }_{2}}{{q}_{1}{\alpha }_{1}{\beta }_{1}}},\\ {\gamma }_{2} & = & 1-|\langle {\phi }_{1}^{(\text{C})}|{\phi }_{2}^{(\text{C})}\rangle |\sqrt{\frac{{q}_{1}{\alpha }_{1}{\beta }_{1}}{{q}_{2}{\alpha }_{2}{\beta }_{2}}}=1-\frac{|\langle {\phi }_{1}^{(\text{B})}|{\phi }_{2}^{(\text{B})}\rangle |}{\sqrt{(1-{\beta }_{1})(1-{\beta }_{2})}}\sqrt{\frac{{q}_{1}{\alpha }_{1}{\beta }_{1}}{{q}_{2}{\alpha }_{2}{\beta }_{2}}}.\end{array}$$

Under the condition $0\le {\gamma }_{i}\le 1-|\langle {\psi }_{1}|{\psi }_{2}\rangle {|}^{2}$, we can show that $({\beta }_{1},{\beta }_{2})$ should satisfy the inequality constraints in Eq. (16). Because a detailed derivation is too lengthy, we omit the derivation. If we substitute $({\gamma }_{1},{\gamma }_{2})$ into the optimization problem, we can obtain Eq. (16).

If $({\gamma }_{1},{\gamma }_{2})$ satisfies ${\gamma }_{1} < 0,{\gamma }_{2} > 1-|\langle {\psi }_{1}|{\psi }_{2}\rangle {|}^{2}$ or ${\gamma }_{1} > 1-|\langle {\psi }_{1}|{\psi }_{2}\rangle {|}^{2},{\gamma }_{2} < 0$, then $({\gamma }_{1},{\gamma }_{2})=(1-|\langle {\psi }_{1}|{\psi }_{2}\rangle {|}^{2},0)$ or $({\gamma }_{1},{\gamma }_{2})=(0,1-|\langle {\psi }_{1}|{\psi }_{2}\rangle {|}^{2})$ becomes an optimal condition. Substituting it into ${P}_{s}^{(\text{B},\text{C},\text{D})}$, we obtain Eq. (20). Although we deals only with the $N=3$ case, we can use this method for any $N$.

### Optimal success probability of the quantum reproducing strategy

We derive the optimal success probability of the quantum reproducing strategy. To make it simple, we consider the $N=2$ case. Then, the success probability is expressed as

$$\begin{array}{rcl}{P}_{rep}^{(\text{B},\text{C})} & = & \frac{1}{2}{\text{Pr}}_{\text{B}}[1|{\rho }_{1}]{\text{Pr}}_{\text{C}}[1|{\rho }_{1}]+\frac{1}{2}{\text{Pr}}_{\text{B}}[2|{\rho }_{2}]{\text{Pr}}_{\text{C}}[2|{\rho }_{2}]\\  & = & \left\{\frac{1}{2},{\text{Pr}}_{\text{B}},[,1,|,{\rho }_{1},],+,\frac{1}{2},{\text{Pr}}_{\text{B}},[,2,|,{\rho }_{2},]\right\}\\  &  & \times \,\{\frac{\frac{1}{2}{\text{Pr}}_{\text{B}}[1|{\rho }_{1}]}{\frac{1}{2}{\text{Pr}}_{\text{B}}[1|{\rho }_{1}]+\frac{1}{2}{\text{Pr}}_{\text{B}}[2|{\rho }_{2}]}{\text{Pr}}_{\text{C}}[1|{\rho }_{1}]\\  &  & +\,\frac{\frac{1}{2}{\text{Pr}}_{\text{B}}[2|{\rho }_{2}]}{\frac{1}{2}{\text{Pr}}_{\text{B}}[1|{\rho }_{1}]+\frac{1}{2}{\text{Pr}}_{\text{B}}[2|{\rho }_{2}]}{\text{Pr}}_{\text{C}}[2|{\rho }_{2}]\}.\end{array}$$

$\frac{1}{2}{\text{Pr}}_{\text{B}}\mathrm{[1|}{\rho }_{1}]+\frac{1}{2}{\text{Pr}}_{\text{B}}\mathrm{[2|}{\rho }_{2}]$ only depends on Bob’s POVM. When Bob’s POVM corresponds to the real vector $({\alpha }_{1},{\alpha }_{2})$^21^, we obtain optimal success probability from the following optimization problem:

$$\begin{array}{ll}\text{maximize} & \frac{1}{2}(r{\alpha }_{1}+\bar{r}{\bar{\alpha }}_{1})+\frac{1}{2}(r{\alpha }_{2}+\bar{r}{\bar{\alpha }}_{2})\\ \text{subject}\,\text{to} & (1-{\alpha }_{1})(1-{\alpha }_{2})\ge {s}^{2},\\  & (1-{\bar{\alpha }}_{1})(1-{\bar{\alpha }}_{2})\ge {\bar{s}}^{2}.\end{array}$$

This optimization problem is partitioned into the following two sub-optimization problem:

$$\begin{array}{ll}\text{maximize} & {\alpha }_{1}+{\alpha }_{2}\\ \text{subject}\,\text{to} & (1-{\alpha }_{1})(1-{\alpha }_{2})\ge {s}^{2}\\ \text{maximize} & {\bar{\alpha }}_{1}+{\bar{\alpha }}_{2}\\ \text{subject}\,\text{to} & (1-{\bar{\alpha }}_{1})(1-{\bar{\alpha }}_{2})\ge {\bar{s}}^{2}\end{array}$$

The optimal solution of the two problems is given as ${\alpha }_{1}={\alpha }_{2}=1-s$ and ${\bar{\alpha }}_{1}={\bar{\alpha }}_{2}=1-\bar{s}$. Hence, we obtain the optimal success probability as $\text{max}\left\{\frac{1}{2}{\text{Pr}}_{\text{B}}\mathrm{[1|}{\rho }_{1}]+\frac{1}{2}{\text{Pr}}_{\text{B}}\mathrm{[2|}{\rho }_{2}]\right\}=r\mathrm{(1}-s)+\bar{r}\mathrm{(1}-\bar{s})$. Because ${\alpha }_{1}={\alpha }_{2}$ and ${\bar{\alpha }}_{1}={\bar{\alpha }}_{2}$, ${\text{Pr}}_{\text{B}}\mathrm{[1|}{\rho }_{1}]={\text{Pr}}_{\text{B}}\mathrm{[2|}{\rho }_{2}]$ also holds. Therefore, we obtain $\frac{\frac{1}{2}{\text{Pr}}_{\text{B}}[i|{\rho }_{i}]}{\frac{1}{2}{\text{Pr}}_{\text{B}}\mathrm{[1|}{\rho }_{1}]+\frac{1}{2}{\text{Pr}}_{\text{B}}\mathrm{[2|}{\rho }_{2}]}=\frac{1}{2}(\forall i)$. This means that Charlie’s success probability is also expressed as $\frac{1}{2}{\text{Pr}}_{\text{C}}\mathrm{[1|}{\rho }_{1}]+\frac{1}{2}{\text{Pr}}_{\text{C}}\mathrm{[2|}{\rho }_{2}]$. In conclusion, the optimal success probability of the quantum reproducing strategy is given as ${(r\mathrm{(1}-s)+\bar{r}\mathrm{(1}-\bar{s}))}^{2}$. Although we consider only the $N=2$ case, this calculation can be applied to any $N$.

### Derivation of secret key rate in multiparty QKD - Part I: Eve’s single trial of eavesdropping

Here, we explain the method to obtain the secret key rate of generalized sequential discrimination. Even though the identical prior probability is used in Result, we consider general prior probabilities given by ${q}_{0}$ and ${q}_{1}$. Then, the entangled state between Alice and Bob is expressed by

$$|\varPsi {\rangle }_{AB}=\sqrt{{q}_{0}}|0{\rangle }_{A}\otimes |{\psi }_{0}{\rangle }_{B}+\sqrt{{q}_{1}}|1{\rangle }_{A}\otimes |{\psi }_{1}{\rangle }_{B}.$$

The quantum channel ${\Lambda }_{B}^{(A\to B)}$ transforms the entangled state $|\varPsi \rangle$ as follows:

$$\begin{array}{rcl}{\sigma }_{AB} & = & ({\text{id}}_{A}\otimes {\Lambda }_{B}^{(A\to B)})(|\Psi \rangle \langle \Psi {|}_{AB})\\  & = & {q}_{0}|0\rangle \langle 0{|}_{A}\otimes \left({\eta }_{AB}|{\psi }_{0}\rangle \langle {\psi }_{0}{|}_{B}+(1-{\eta }_{AB})\frac{{I}_{B}}{2}\right)\\  &  & +\,\sqrt{{q}_{0}{q}_{1}}|0\rangle \langle 1{|}_{A}\otimes \left({\eta }_{AB}|{\psi }_{0}\rangle \langle {\psi }_{1}{|}_{B}+(1-{\eta }_{AB})\frac{{I}_{B}}{2}\right)\\  &  & +\,\sqrt{{q}_{0}{q}_{1}}|1\rangle \langle 0{|}_{A}\otimes \left({\eta }_{AB}|{\psi }_{1}\rangle \langle {\psi }_{0}{|}_{B}+(1-{\eta }_{AB})\frac{{I}_{B}}{2}\right)\\  &  & +\,{q}_{1}|1\rangle \langle 1{|}_{A}\otimes \left({\eta }_{AB}|{\psi }_{1}\rangle \langle {\psi }_{1}{|}_{B}+(1-{\eta }_{AB})\frac{{I}_{B}}{2}\right)\\  & = & {\eta }_{AB}|\Psi \rangle \langle \Psi {|}_{AB}+(1-{\eta }_{AB})|{e}_{+}^{(A\to B)}\rangle \langle {e}_{+}^{(A\to B)}{|}_{A}\otimes \frac{{I}_{B}}{2}.\end{array}$$

Here, $|{e}_{+}^{(A\to B)}\rangle =\sqrt{{q}_{0}}|0\rangle +\sqrt{{q}_{1}}|1\rangle$. When ${q}_{0}={q}_{1}$, one can have $|{e}_{+}^{(A\to B)}\rangle =|+\rangle$. The purification ${\sigma }_{AB}$ becomes

$$|\Gamma {\rangle }_{ABE}=\sqrt{{\eta }_{AB}}|\Psi {\rangle }_{AB}\otimes |?{\rangle }_{E}+\sqrt{1-{\eta }_{AB}}|{e}_{+}^{(A\to B)}{\rangle }_{A}\otimes |{\phi }_{+}{\rangle }_{BE}.$$

Because $|?\rangle$ and $|{\phi }_{+}\rangle$ are orthogonal to each other, the relation of ${\text{Tr}}_{E}|\Gamma \rangle \langle \Gamma {|}_{ABE}={\sigma }_{AB}$ is obvious. Therefore, two marginal probabilities ${P}_{A}(i)$ and ${P}_{E}(k)$ are evaluated as follows:

$$\begin{array}{rcl}{P}_{A}(i) & = & {\text{Tr}}_{ABE}[|\Gamma \rangle \langle \Gamma {|}_{ABE}|i\rangle \langle i{|}_{A}\otimes {I}_{BE}]\\  & = & {\text{Tr}}_{A}[{\text{Tr}}_{BE}(|\Gamma \rangle \langle \Gamma {|}_{ABE})|i\rangle \langle i{|}_{A}]\\  & = & {\text{Tr}}_{A}[{\text{Tr}}_{B}\{{\eta }_{AB}|\Psi \rangle \langle \Psi {|}_{AB}\\  &  & +\,(1-{\eta }_{AB})|{e}_{+}^{(A\to B)}\rangle \langle {e}_{+}^{(A\to B)}|\otimes \frac{{I}_{B}}{2}\}|i\rangle \langle i{|}_{A}]\\  & = & {\eta }_{AB}{q}_{i}+(1-{\eta }_{AB}){|\langle i|{e}_{+}^{(A\to B)}\rangle |}^{2}={q}_{i},\\ {P}_{E}(k) & = & {\text{Tr}}_{ABE}[|\Gamma \rangle \langle \Gamma {|}_{ABE}{I}_{AB}\otimes |k\rangle \langle k{|}_{E}]\\  & = & {\text{Tr}}_{E}[{\text{Tr}}_{AB}\{|\Gamma \rangle \langle \Gamma {|}_{ABE}\}|k\rangle \langle k{|}_{E}]\\  & = & (1-{\eta }_{AB})\langle k|\frac{{I}_{E}}{2}|k\rangle \\  & = & (1-{\eta }_{AB})\frac{1}{2}\langle k|k\rangle =\frac{1-{\eta }_{AB}}{2}.\end{array}$$

To evaluate the marginal probabilities ${P}_{AB}(i,j)$ and ${P}_{BE}(j,k)$, we should use POVM element ${M}_{i}$ of Bob. Using the condition of overlap $s=\langle {\psi }_{0}|{\psi }_{1}\rangle$ in two pure states of Alice, we can construct an explicit form of the two pure states as follows:

$$|{\psi }_{0}\rangle =\sqrt{\frac{1+s}{2}}|0\rangle +\sqrt{\frac{1-s}{2}}|1\rangle ,\,|{\psi }_{1}\rangle =\sqrt{\frac{1+s}{2}}|0\rangle -\sqrt{\frac{1-s}{2}}|1\rangle .$$

The POVM element of Bob can be given as ${\alpha }_{i}|{\alpha }_{i}\rangle \langle {\alpha }_{i}|$^12^, where $|{\alpha }_{0}\rangle$ and $|{\alpha }_{1}\rangle$ are expressed as follows:

42 $$|{\alpha }_{0}\rangle =\frac{1}{\sqrt{2(1+s)}}|0\rangle +\frac{1}{\sqrt{2(1-s)}}|1\rangle ,\,|{\alpha }_{1}\rangle =\frac{1}{\sqrt{2(1+s)}}|0\rangle -\frac{1}{\sqrt{2(1-s)}}|1\rangle .$$

The state $|{\alpha }_{i}\rangle$ satisfies the following relations: (i) $\langle {\alpha }_{i}|{\alpha }_{i}\rangle =1/(1-{s}^{2})$, (ii) $|\langle k|{\alpha }_{j}\rangle |=1/\sqrt{2\{1+{(-1)}^{k}s\}}$. Using these relations, the marginal probabilities ${P}_{AB}(i,j)$ and ${P}_{BE}(j,k)$ can be evaluated as

$$\begin{array}{rcl}{P}_{AB}(i,j) & = & {\text{Tr}}_{ABE}\,[|\Gamma \rangle \langle \Gamma {|}_{ABE}|i\rangle \langle i{|}_{A}\otimes {M}_{j}\otimes {I}_{E}]\\  & = & {\text{Tr}}_{AB}\,[{\text{Tr}}_{E}\{|\Gamma \rangle \langle \Gamma {|}_{ABE}\}\}|i\rangle \langle i{|}_{A}\otimes {M}_{j}]\\  & = & {\text{Tr}}_{AB}\,\left[\left\{{\eta }_{AB}|\Psi \rangle \langle \Psi {|}_{AB}+\mathrm{(1}-{\eta }_{AB})|{e}_{+}^{(A\to B)}\rangle \langle {e}_{+}^{(A\to B)}|\otimes \frac{{I}_{B}}{2}\right\}|i\rangle \langle i{|}_{A}\otimes {M}_{j}\right]\\  & = & {\eta }_{AB}\langle \Psi |(|i\rangle \langle i{|}_{A}\otimes {M}_{j})|\Psi \rangle +\mathrm{(1}-{\eta }_{AB})|\langle i|{e}_{+}^{(A\to B)}\rangle {|}^{2}\frac{1}{2}{\text{Tr}}_{B}{M}_{j}\\  & = & {\eta }_{AB}\langle \Psi |(|i\rangle \langle i{|}_{A}\otimes {M}_{j})|\Psi \rangle +\mathrm{(1}-{\eta }_{AB})|\langle i|{e}_{+}^{(A\to B)}\rangle {|}^{2}\frac{{\alpha }_{j}}{2}\langle {\alpha }_{j}|{\alpha }_{j}\rangle \\  & = & {\eta }_{AB}{q}_{i}{\alpha }_{i}{\delta }_{ij}+\mathrm{(1}-{\eta }_{AB}){q}_{i}\frac{{\alpha }_{j}}{\mathrm{2(1}-{s}^{2})},\\ {P}_{BE}(j,k) & = & {\text{Tr}}_{ABE}[|\Gamma \rangle \langle \Gamma {|}_{ABE}{I}_{A}\otimes {M}_{j}\otimes |k\rangle \langle k{|}_{E}]\\  & = & {\text{Tr}}_{BE}[{\text{Tr}}_{A}\{|\Gamma \rangle \langle \Gamma {|}_{ABE}\}{M}_{j}\otimes |k\rangle \langle k{|}_{E}]\\  & = & \frac{1}{2}\mathrm{(1}-{\eta }_{AB})\langle k|{M}_{j}|k\rangle \\  & = & \frac{1-{\eta }_{AB}}{2}{\alpha }_{j}|\langle k|{\alpha }_{j}\rangle {|}^{2}\\  & = & \frac{\mathrm{(1}-{\eta }_{AB}){\alpha }_{j}}{\mathrm{4\{1}+{(-\mathrm{1)}}^{k}s\}}\mathrm{}.\end{array}$$

We can see that when ${q}_{0}={q}_{1}$, four marginal probabilities are given by Eq. (38).

When Bob eliminates an inconclusive result, the measurement of Bob provides the following ensemble:

$$\begin{array}{l}\frac{{I}_{A}\otimes {K}_{0}|\Psi \rangle \langle \Psi {|}_{AB}{I}_{A}\otimes {K}_{0}^{\dagger }+{I}_{A}\otimes {K}_{1}|\Psi \rangle \langle \Psi {|}_{AB}{I}_{A}\otimes {K}_{1}^{\dagger }}{\text{Tr}\{{I}_{A}\otimes {K}_{0}|\Psi \rangle \langle \Psi {|}_{AB}{I}_{A}\otimes {K}_{0}^{\dagger }+{I}_{A}\otimes {K}_{1}|\Psi \rangle \langle \Psi {|}_{AB}{I}_{A}\otimes {K}_{1}^{\dagger }\}}\\ \begin{array}{rcl} & = & \frac{{q}_{0}{\alpha }_{0}}{{q}_{0}{\alpha }_{0}+{q}_{1}{\alpha }_{1}}|0\rangle \langle 0{|}_{A}\otimes |{\phi }_{0}^{(B)}\rangle \langle {\phi }_{0}^{(B)}{|}_{C}\\  &  & +\,\frac{{q}_{1}{\alpha }_{1}}{{q}_{0}{\alpha }_{0}+{q}_{1}{\alpha }_{1}}|1\rangle \langle 1{|}_{A}\otimes |{\phi }_{1}^{(B)}\rangle \langle {\phi }_{1}^{(B)}{|}_{C}.\end{array}\end{array}$$

Here, ${K}_{i}=\sqrt{{\alpha }_{i}}|{\phi }_{i}^{(B)}{\rangle }_{C}\langle {\alpha }_{i}{|}_{B}$ is a linear map of ${{\mathscr{H}}}_{B}\to {{\mathscr{H}}}_{C}$. The quantum state between Alice and Charlie becomes the following entangled state

$$\begin{array}{rcl}|{\varPhi }^{(B)}{\rangle }_{AB} & = & \sqrt{\frac{{q}_{0}{\alpha }_{0}}{{q}_{0}{\alpha }_{0}+{q}_{1}{\alpha }_{1}}}|0{\rangle }_{A}\otimes |{\phi }_{0}^{(B)}{\rangle }_{C}+\sqrt{\frac{{q}_{1}{\alpha }_{1}}{{q}_{0}{\alpha }_{0}+{q}_{1}{\alpha }_{1}}}|1{\rangle }_{A}\otimes |{\phi }_{1}^{(B)}{\rangle }_{C}\\  & = & \sqrt{{Q}_{0}^{(B)}}|0{\rangle }_{A}\otimes |{\phi }_{0}^{(B)}{\rangle }_{C}+\sqrt{{Q}_{1}^{(B)}}|1{\rangle }_{A}\otimes |{\phi }_{1}^{(B)}{\rangle }_{C}\mathrm{}.\end{array}$$

When ${q}_{0}={q}_{1}$, the optimal condition is given by ${\alpha }_{0}={\alpha }_{1}=1-{s}^{1/N}$. Therefore, we can find ${Q}_{0}^{(B)}={Q}_{1}^{(B)}$.

Two post-measurement states $|{\phi }_{0}^{(B)}\rangle$ and $|{\phi }_{1}^{(B)}\rangle$ satisfies the overlap condition $s{\prime} =\langle {\phi }_{0}^{(B)}|{\phi }_{1}^{(B)}\rangle =s/\sqrt{(1-{\alpha }_{0})(1-{\alpha }_{1})}$ and the explicit forms of $|{\phi }_{0}^{(B)}\rangle$ and $|{\phi }_{1}^{(B)}\rangle$ become

$$|{\phi }_{0}^{(B)}\rangle =\sqrt{\frac{1+s{\prime} }{2}}|0\rangle +\sqrt{\frac{1-s{\prime} }{2}}|1\rangle ,\,|{\phi }_{1}^{(B)}\rangle =\sqrt{\frac{1+s{\prime} }{2}}|0\rangle -\sqrt{\frac{1-s{\prime} }{2}}|1\rangle .$$

The POVM element of Charlie is given by ${\beta }_{i}|{\beta }_{i}\rangle \langle {\beta }_{i}|$, where $|{\beta }_{0}\rangle$ and $|{\beta }_{1}\rangle$ are expressed by

43 $$|{\beta }_{0}\rangle =\frac{1}{\sqrt{2(1+s{\prime} )}}|0\rangle +\frac{1}{\sqrt{2(1-s{\prime} )}}|1\rangle ,\,|{\beta }_{1}\rangle =\frac{1}{\sqrt{2(1+s{\prime} )}}|0\rangle -\frac{1}{\sqrt{2(1-s{\prime} )}}|1\rangle .$$

We can see the relation of $\langle {\phi }_{i}^{(B)}|{\alpha }_{j}\rangle ={\delta }_{ij}$ from Eq. (43). Substituting Eq. (43) into the marginal probabilities, we can obtain ${P}_{AC}$ and ${P}_{CE}$. Especially, when ${q}_{0}={q}_{1}$, from ${\alpha }_{0}={\alpha }_{1}=1-{s}^{1/N}$, one can obtain $s{\prime} ={s}^{(N-1)/N}$. Using the similar method, for arbitrary $X$, we can find ${P}_{AX}$ and ${P}_{XE}$.

### Derivation of secret key rate in multiparty QKD - Part II: Eve’s multi-trial of eavesdropping

Here, we show how to evaluate the secret key rate of the case where Eve performs eavesdropping between Alice and Bob and between Bob and Charlie. Here, prior probabilities ${q}_{0}$ and ${q}_{1}$ are considered to be arbitrary values. Suppose that Eve performs eavesdropping between Alice and Bob, using a quantum memory ${E}_{B}$. Then, Alice, Bob, and Eve share the following quantum state:

$$|\Gamma {\rangle }_{AB{E}_{B}}=\sqrt{{\eta }_{AB}}|\Psi {\rangle }_{AB}\otimes |?{\rangle }_{{E}_{B}}+\sqrt{1-{\eta }_{AB}}|{e}_{+}^{(A\to B)}{\rangle }_{A}\otimes |{\phi }_{+}{\rangle }_{B{E}_{B}}.$$

Let us assume that the Kraus operator of Bob is given as $\{{K}_{0}^{(B)},{K}_{1}^{(B)},{K}_{?}^{(B)}\}$. Then, Kraus operator ${K}_{0}^{(B)},{K}_{1}^{(B)}$ transforms $|\Gamma {\rangle }_{AB{E}_{B}}$ as follows:

$$\begin{array}{rcl}{I}_{A}\otimes {K}_{0}^{(B)}\otimes {I}_{{E}_{B}}|\Gamma {\rangle }_{AB{E}_{B}} & = & \{\sqrt{{\eta }_{AB}{q}_{0}{\alpha }_{0}}|0{\rangle }_{A}\otimes |?{\rangle }_{{E}_{B}}\\  & = & +\,\sqrt{\frac{(1-{\eta }_{AB}){\alpha }_{0}}{2}}|{e}_{+}^{(A\to B)}{\rangle }_{A}\otimes |{\alpha }_{0}{\rangle }_{{E}_{B}}\}\otimes |{\phi }_{0}^{(B)}{\rangle }_{C}\\  & = & |{ {\mathcal E} }_{0}{\rangle }_{A{E}_{B}}\otimes |{\phi }_{0}^{(B)}{\rangle }_{C},\\ {I}_{A}\otimes {K}_{1}^{(B)}\otimes {I}_{{E}_{B}}|\Gamma {\rangle }_{AB{E}_{B}} & = & \{\sqrt{{\eta }_{AB}{q}_{1}{\alpha }_{1}}|1{\rangle }_{A}\otimes |?{\rangle }_{{E}_{B}}\\  & = & +\,\sqrt{\frac{(1-{\eta }_{AB}){\alpha }_{1}}{2}}|{e}_{+}^{(A\to B)}{\rangle }_{A}\otimes |{\alpha }_{1}{\rangle }_{{E}_{B}}\}\otimes |{\phi }_{1}^{(B)}{\rangle }_{C}\\  & = & |{ {\mathcal E} }_{1}{\rangle }_{A{E}_{B}}\otimes |{\phi }_{1}^{(B)}{\rangle }_{C}.\end{array}$$

When ${\eta }_{AB}=1$, we have $|{ {\mathcal E} }_{i}\rangle =\sqrt{{q}_{i}{\alpha }_{i}}|i{\rangle }_{A}\otimes |?{\rangle }_{{E}_{B}}$. Therefore, the argument of this case is identical to the security analysis of Part I.

After Bob performs a post-processing, $|\Gamma {\rangle }_{AB{E}_{B}}$ becomes the following tripartite state:

$$\begin{array}{rcl}{\sigma }_{AC{E}_{B}} & = & \frac{{I}_{A}\otimes {K}_{0}^{(B)}\otimes {I}_{{E}_{B}}|\Gamma \rangle \langle \Gamma |{I}_{A}\otimes {K}_{0}^{(B)\dagger }\otimes {I}_{{E}_{B}}+{I}_{A}\otimes {K}_{1}^{(B)}\otimes {I}_{{E}_{B}}|\Gamma \rangle \langle \Gamma |{I}_{A}\otimes {K}_{1}^{(B)\dagger }\otimes {I}_{{E}_{B}}}{(\langle \Gamma |{I}_{A}\otimes {K}_{0}^{(B)\dagger }\otimes {I}_{{E}_{B}})({I}_{A}\otimes {K}_{0}^{(B)}\otimes {I}_{{E}_{B}}|\Gamma \rangle )+(\langle \Gamma |{I}_{A}\otimes {K}_{1}^{(B)\dagger }\otimes {I}_{{E}_{B}})({I}_{A}\otimes {K}_{1}^{(B)}\otimes {I}_{{E}_{B}}|\Gamma \rangle )}\\  & = & {\mathscr{N}}({\eta }_{AB})\{|{ {\mathcal E} }_{0}\rangle \langle { {\mathcal E} }_{0}{|}_{A{E}_{B}}\otimes |{\phi }_{0}^{(B)}\rangle \langle {\phi }_{0}^{(B)}{|}_{C}+|{ {\mathcal E} }_{1}\rangle \langle { {\mathcal E} }_{1}{|}_{A{E}_{B}}\otimes |{\phi }_{1}^{(B)}\rangle \langle {\phi }_{1}^{(B)}{|}_{C}\}.\end{array}$$

When Eve performs eavesdropping between Bob and Charlie, ${\sigma }_{AC{E}_{B}}$ becomes

$${\text{id}}_{A{E}_{B}}\otimes {\Lambda }_{C}^{(B\to C)}({\sigma }_{AC{E}_{B}})={\eta }_{BC}{\sigma }_{AC{E}_{B}}+\mathrm{(1}-{\eta }_{BC}){\tau }_{AC{E}_{B}}\mathrm{}.$$

Here, ${\tau }_{AC{E}_{B}}$ is expressed by

$${\tau }_{AC{E}_{B}}={\mathscr{N}}({\eta }_{AB})\{|{ {\mathcal E} }_{0}\rangle \langle { {\mathcal E} }_{0}{|}_{A{E}_{B}}+|{ {\mathcal E} }_{1}\rangle \langle { {\mathcal E} }_{1}{|}_{A{E}_{B}}\}\otimes \frac{{I}_{C}}{2}\mathrm{}.$$

Let us denote the purifications of ${\eta }_{BC}{\sigma }_{AC{E}_{B}}$ and $\mathrm{(1}-{\eta }_{BC}){\tau }_{AC{E}_{B}}$ as $|{\eta }_{BC}{\sigma }_{AC{E}_{B}}{\rangle }_{AC{E}_{B}{E}_{C}}$ and $|(1-{\eta }_{BC}){\tau }_{AC{E}_{B}}{\rangle }_{AC{E}_{B}{E}_{C}}$, respectively. Here, ${E}_{C}$ is the quantum system of Eve which operates between Bob and Charlie. If $|{\eta }_{BC}{\sigma }_{AC{E}_{B}}{\rangle }_{AC{E}_{B}{E}_{C}}$ and $|\mathrm{(1}-{\eta }_{BC}){\tau }_{AC{E}_{B}}{\rangle }_{AC{E}_{B}{E}_{C}}$ are orthogonal to each other, the following equality holds:

$$\begin{array}{c}{\text{Tr}}_{{E}_{C}}(|{\eta }_{BC}{\sigma }_{AC{E}_{B}}\rangle +|\mathrm{(1}-{\eta }_{BC}){\tau }_{AC{E}_{B}}\rangle )(\langle {\eta }_{BC}{\sigma }_{AC{E}_{B}}|+{\langle \mathrm{(1}-{\eta }_{BC}){\tau }_{AC{E}_{B}}|)}_{AC{E}_{B}{E}_{C}}\\ \,=\,{\eta }_{BC}{\sigma }_{AC{E}_{B}}+\mathrm{(1}-{\eta }_{BC}){\tau }_{AC{E}_{B}}\mathrm{}.\end{array}$$

Using $\{|{?}_{0}\rangle ,|{?}_{1}\rangle ,|00\rangle ,|01\rangle ,|10\rangle ,|11\rangle \}$ which consists of orthogonal basis of ${E}_{C}$, we can construct purification of ${\text{id}}_{A{E}_{B}}\otimes {\Lambda }_{C}^{(B\to C)}({\sigma }_{AC{E}_{B}})$. First, $|{\eta }_{BC}{\sigma }_{AB{E}_{B}}\rangle$ can be constructed by the basis $|{?}_{0}\rangle ,|{?}_{1}\rangle$. Second, $|\mathrm{(1}-{\eta }_{AB}){\tau }_{AC{E}_{B}}\rangle$ can be constructed by $|00\rangle ,|01\rangle ,|10\rangle ,|11\rangle$}. Then, $|{\eta }_{BC}{\sigma }_{AC{E}_{B}}\rangle$ and $|\mathrm{(1}-{\eta }_{BC}){\tau }_{AC{E}_{B}}\rangle$ are orthogonal to each other. In conclusion, the purification of ${\text{id}}_{A{E}_{B}}\otimes {\Lambda }_{C}^{(B\to C)}({\sigma }_{AC{E}_{B}})$ can be given as follows:

$$\begin{array}{rcl}|\Gamma {\rangle }_{AC{E}_{B}{E}_{C}} & = & |{\eta }_{BC}{\sigma }_{AC{E}_{B}}\rangle +|\mathrm{(1}-{\eta }_{BC}){\tau }_{AC{E}_{B}}\rangle \\  & = & \sqrt{{\mathscr{N}}({\eta }_{AB})}[\sqrt{{\eta }_{BC}}\{|{ {\mathcal E} }_{0}{\rangle }_{A{E}_{B}}\otimes |{\phi }_{0}^{(B)}{\rangle }_{C}\otimes |{?}_{0}{\rangle }_{{E}_{C}}\\  &  & +\,|{ {\mathcal E} }_{1}{\rangle }_{A{E}_{B}}\otimes |{\phi }_{1}^{(B)}{\rangle }_{C}\otimes |{?}_{1}{\rangle }_{{E}_{C}}\}\\  &  & +\,\sqrt{1-{\eta }_{BC}}|{e}_{+}^{(A\to B\to C)}{\rangle }_{A{E}_{B}{E}_{C2}}\otimes |{\phi }_{+}{\rangle }_{C{E}_{C1}}].\end{array}$$

Here, $$|{\phi }_{+}\rangle =(|00\rangle +|11\rangle )/\sqrt{2}$$, $$|{e}_{+}^{(A\to B\to C)}\rangle =|0\rangle \otimes |{ {\mathcal E} }_{0}\rangle +|1\rangle \otimes |{ {\mathcal E} }_{1}\rangle $$.

To evaluate the secret key rate between Alice and Charlie, one must obtain the marginal probabilities ${P}_{A},{P}_{C},{P}_{CA},{P}_{CE}$. First, ${P}_{A}(i)$ can be evaluated as follows:

$$\begin{array}{rcl}{P}_{A}(i) & = & \text{Tr}[|i\rangle \langle i{|}_{A}\otimes {I}_{C{E}_{B}{E}_{C}}|\Gamma \rangle \langle \Gamma {|}_{AC{E}_{B}{E}_{C}}]\\  & = & {\mathscr{N}}({\eta }_{AB})\mathop{\underbrace{\left[{\eta }_{AB}{\alpha }_{0}+\frac{\mathrm{(1}-{\eta }_{AB})({\alpha }_{0}+{\alpha }_{1})}{\mathrm{2(1}-{s}^{2})}\right]{q}_{0}}}\limits_{{{\mathscr{N}}}_{0}({\eta }_{AB})}{\delta }_{i0}\\  &  & +\,{\mathscr{N}}({\eta }_{AB})\mathop{\underbrace{\left[{\eta }_{AB}{\alpha }_{1}+\frac{\mathrm{(1}-{\eta }_{AB})({\alpha }_{0}+{\alpha }_{1})}{\mathrm{2(1}-{s}^{2})}\right]{q}_{1}}}\limits_{{{\mathscr{N}}}_{1}({\eta }_{AB})}{\delta }_{i1}\mathrm{}.\end{array}$$

Because of ${\mathscr{N}}({\eta }_{AB})={[{{\mathscr{N}}}_{0}({\eta }_{AB})+{{\mathscr{N}}}_{1}({\eta }_{AB})]}^{-1}$, ${P}_{A}(i)$ is expressed as

$${P}_{A}(i)=\frac{{{\mathscr{N}}}_{0}({\eta }_{AB})}{{{\mathscr{N}}}_{0}({\eta }_{AB})+{{\mathscr{N}}}_{1}({\eta }_{AB})}{\delta }_{i0}+\frac{{{\mathscr{N}}}_{1}({\eta }_{AB})}{{{\mathscr{N}}}_{0}({\eta }_{AB})+{{\mathscr{N}}}_{1}({\eta }_{AB})}{\delta }_{i1}.$$

That is, ${P}_{A}(i)$ is a prior probability after Eve’s eavesdropping and Bob’s measurement. Specially, when ${\eta }_{AB}=1$, ${P}_{A}(i)$ is given as follows:

$$\begin{array}{rcl}{P}_{A}(i) & = & \frac{{q}_{0}{\alpha }_{0}}{{q}_{0}{\alpha }_{0}+{q}_{1}{\alpha }_{1}}{\delta }_{i0}+\frac{{q}_{1}{\alpha }_{1}}{{q}_{0}{\alpha }_{0}+{q}_{1}{\alpha }_{1}}{\delta }_{i1}\\  & = & \frac{{q}_{0}\langle {\psi }_{0}|{M}_{0}^{(B)}|{\psi }_{0}\rangle }{{q}_{0}\langle {\psi }_{0}|{M}_{0}^{(B)}|{\psi }_{0}\rangle +{q}_{1}\langle {\psi }_{1}|{M}_{1}^{(B)}|{\psi }_{1}\rangle }{\delta }_{i0}\\  &  & +\,\frac{{q}_{1}\langle {\psi }_{1}|{M}_{1}^{(B)}|{\psi }_{1}\rangle }{{q}_{0}\langle {\psi }_{0}|{M}_{0}^{(B)}|{\psi }_{0}\rangle +{q}_{1}\langle {\psi }_{1}|{M}_{1}^{(B)}|{\psi }_{1}\rangle }{\delta }_{i1}.\end{array}$$

Second, ${P}_{E}={P}_{{E}_{B}{E}_{C}}(l,(m,n))$ is evaluated as follows:

$$\begin{array}{rcl}{P}_{{E}_{B}{E}_{C}}(l,(m,n)) & = & \text{Tr}[{I}_{A}\otimes |l\rangle \langle l{|}_{{E}_{B}}\otimes |mn\rangle \langle mn{|}_{{E}_{C}}|\Gamma \rangle \langle \Gamma |]\\  & = & {\text{Tr}}_{{E}_{B}{E}_{C}}[|l\rangle \langle l{|}_{{E}_{B}}\otimes |mn\rangle \langle mn{|}_{{E}_{C}}{\text{Tr}}_{AC}|\Gamma \rangle \langle \Gamma |].\end{array}$$

Here, ${\text{Tr}}_{AC}|\Gamma \rangle \langle \Gamma {|}_{AC{E}_{B}{E}_{C}}$ is given by

$$\begin{array}{rcl}{\text{Tr}}_{AC}|\Gamma \rangle \langle \Gamma {|}_{AC{E}_{B}{E}_{C}} & = & \frac{1-{\eta }_{BC}}{2}{\mathscr{N}}({\eta }_{AB})[{\text{Tr}}_{A}|{ {\mathcal E} }_{0}\rangle \langle { {\mathcal E} }_{0}{|}_{A{E}_{B}}\otimes |0\rangle \langle 0{|}_{{E}_{C2}}\\  &  & +\,{\text{Tr}}_{A}|{ {\mathcal E} }_{0}\rangle \langle { {\mathcal E} }_{1}{|}_{A{E}_{B}}\otimes |0\rangle \langle 1{|}_{{E}_{C2}}\\  &  & +\,{\text{Tr}}_{A}|{ {\mathcal E} }_{1}\rangle \langle { {\mathcal E} }_{0}{|}_{A{E}_{B}}\otimes |1\rangle \langle 0{|}_{{E}_{C2}}\\  &  & +\,{\text{Tr}}_{A}|{ {\mathcal E} }_{1}\rangle \langle { {\mathcal E} }_{1}{|}_{A{E}_{B}}\otimes |1\rangle \langle 1{|}_{{E}_{C2}}+\varOmega (|{?}_{0}\rangle ,|{?}_{1}\rangle )]\otimes \frac{{I}_{{E}_{C1}}}{2}\mathrm{}.\end{array}$$

Here, $\varOmega (|{?}_{0}\rangle ,|{?}_{1}\rangle )$ is an operator containing $|{?}_{0}{\rangle }_{{E}_{C}},|{?}_{1}{\rangle }_{{E}_{C}}$. Therefore, in $\forall l,m,n\in \{0,1\}$, we obtain $\langle lmn|{I}_{{E}_{C1}}\otimes \varOmega (|{?}_{0}\rangle ,|{?}_{1}\rangle )|lmn\rangle =0$. Then, ${P}_{E}$ becomes

$$\begin{array}{l}{P}_{{E}_{B}{E}_{C}}(l,(m,n))\\ \begin{array}{rcl} & = & \frac{\mathrm{(1}-{\eta }_{BC}){\mathscr{N}}({\eta }_{AB})}{2}{\text{Tr}}_{{E}_{B}{E}_{C}}[|l\rangle \langle l{|}_{{E}_{B}}\otimes |mn\rangle \langle mn{|}_{{E}_{C}}\\  &  & \times \,\{{\text{Tr}}_{A}|{ {\mathcal E} }_{0}\rangle \langle { {\mathcal E} }_{0}{|}_{A{E}_{B}}\otimes |0\rangle \langle 0{|}_{{E}_{C2}}\\  &  & +\,{\text{Tr}}_{A}|{ {\mathcal E} }_{0}\rangle \langle { {\mathcal E} }_{1}{|}_{A{E}_{B}}\otimes |0\rangle \langle 1{|}_{{E}_{C2}}\\  &  & +\,{\text{Tr}}_{A}|{ {\mathcal E} }_{1}\rangle \langle { {\mathcal E} }_{0}{|}_{A{E}_{B}}\otimes |1\rangle \langle 0{|}_{{E}_{C2}}\\  &  & +\,{\text{Tr}}_{A}|{ {\mathcal E} }_{1}\rangle \langle { {\mathcal E} }_{1}{|}_{A{E}_{B}}\otimes |1\rangle \langle 1{|}_{{E}_{C2}}\}\otimes \frac{{I}_{{E}_{C1}}}{2}]\\  & = & \frac{\mathrm{(1}-{\eta }_{BC}\mathrm{)(1}-{\eta }_{AB}){\alpha }_{0}}{\mathrm{4\{1}+{(-\mathrm{1)}}^{l}s\}}{\mathscr{N}}({\eta }_{AB}){\delta }_{n0}\\  &  & +\,\frac{\mathrm{(1}-{\eta }_{BC}\mathrm{)(1}-{\eta }_{AB}){\alpha }_{1}}{\mathrm{4\{1}+{(-\mathrm{1)}}^{l}s\}}{\mathscr{N}}({\eta }_{AB}){\delta }_{n1}\mathrm{}.\end{array}\end{array}$$

Third, the marginal probability ${P}_{AC}(i,j)$ is evaluated as follows:

$$\begin{array}{rcl}{P}_{AC}(i,j) & = & \text{Tr}[|i\rangle \langle i{|}_{A}\otimes {\beta }_{j}|{\beta }_{j}\rangle \langle {\beta }_{j}{|}_{C}\otimes {I}_{{E}_{B}{E}_{C}}|\Gamma \rangle \langle \Gamma |]\\  & = & {\text{Tr}}_{AC}[|i\rangle \langle i{|}_{A}\otimes {\beta }_{j}|{\beta }_{j}\rangle \langle {\beta }_{j}{|}_{C}{\text{Tr}}_{{E}_{B}{E}_{C}}|\Gamma \rangle \langle \Gamma |\mathrm{]}.\end{array}$$

Here, ${\text{Tr}}_{{E}_{B}{E}_{C}}|\Gamma \rangle \langle \Gamma |$ is given by

$$\begin{array}{rcl}{\text{Tr}}_{{E}_{B}{E}_{C}}|\Gamma \rangle \langle \Gamma {|}_{AC{E}_{B}{E}_{C}} & = & {\eta }_{BC}{\mathscr{N}}({\eta }_{AB})[\{{\eta }_{AB}{q}_{0}{\alpha }_{0}|0\rangle \langle 0{|}_{A}\\  &  & +\,\frac{\mathrm{(1}-{\eta }_{AB}){\alpha }_{0}}{\mathrm{2(1}-{s}^{2})}|{e}_{+}^{(A\to B)}\rangle \langle {e}_{+}^{(A\to B)}{|}_{A}\}\otimes |{\phi }_{0}^{(B)}\rangle \langle {\phi }_{0}^{(B)}{|}_{C}\\  &  & +\,\{{\eta }_{AB}{q}_{1}{\alpha }_{1}|1\rangle \langle 1{|}_{A}\\  &  & +\,\frac{\mathrm{(1}-{\eta }_{AB}){\alpha }_{1}}{\mathrm{2(1}-{s}^{2})}|{e}_{+}^{(A\to B)}\rangle \langle {e}_{+}^{(A\to B)}{|}_{A}\}\otimes |{\phi }_{1}^{(B)}\rangle \langle {\phi }_{1}^{(B)}{|}_{C}]\\  &  & +\,\mathrm{(1}-{\eta }_{BC}){\mathscr{N}}({\eta }_{AB})\{{\eta }_{AB}{q}_{0}{\alpha }_{0}|0\rangle \langle 0{|}_{A}+{\eta }_{AB}{q}_{0}{\alpha }_{1}|1\rangle \langle 1{|}_{A}\\  &  & +\,\frac{\mathrm{(1}-{\eta }_{AB})({\alpha }_{0}+{\alpha }_{1})}{\mathrm{2(1}-{s}^{2})}|{e}_{+}^{(A\to B)}\rangle \langle {e}_{+}^{(A\to B)}{|}_{A}\}\otimes \frac{{I}_{C}}{2}\mathrm{}.\end{array}$$

Then, ${P}_{AC}(i,j)$ is expressed as follows:

$$\begin{array}{rcl}{P}_{AC}(i,j) & = & {\eta }_{BC}{\mathscr{N}}({\eta }_{AB})\frac{{\beta }_{j}}{1-{s{\prime} }^{2}}\{{\eta }_{AB}{q}_{j}{\alpha }_{j}{\delta }_{ij}\\  &  & +\,\frac{\mathrm{(1}-{\eta }_{AB}){\alpha }_{j}}{\mathrm{2(1}-{s}^{2})}({q}_{0}{\delta }_{i0}+{q}_{1}{\delta }_{i1})\}\\  &  & +\,\mathrm{(1}-{\eta }_{BC}){\mathscr{N}}({\eta }_{AB})\frac{{\beta }_{j}}{1-{s{\prime} }^{2}}\{{\eta }_{AB}{q}_{0}{\alpha }_{0}{\delta }_{i0}\\  &  & +\,{\eta }_{AB}{q}_{1}{\alpha }_{1}{\delta }_{i1}+\frac{\mathrm{(1}-{\eta }_{AB})({\alpha }_{0}+{\alpha }_{1})}{\mathrm{2(1}-{s}^{2})}({q}_{0}{\delta }_{i0}+{q}_{1}{\delta }_{i1})\}.\end{array}$$

Here, $s{\prime}$ is an overlap between the post-measurement states of Bob $|{\phi }_{0}^{(B)}\rangle$ and $|{\phi }_{1}^{(B)}\rangle$.

Fourth, to obtain ${P}_{E}(p)$, we can evaluate ${I}_{AC}\otimes {\langle {\pi }_{p}{|}_{{E}_{B}{E}_{C}}|\Gamma \rangle }_{AC{E}_{B}{E}_{C}}$ as follows:

44 $$\begin{array}{l}{({I}_{AC}\otimes \langle {\pi }_{p}{|}_{{E}_{B}{E}_{C}})|\Gamma \rangle }_{AC{E}_{B}{E}_{C}}\\ \begin{array}{rcl} & = & \{{I}_{AC}\otimes \mathop{\sum }\limits_{q\mathrm{=1}}^{18}\,{V}_{pq}^{\ast }\langle q{|}_{{E}_{B}{E}_{C}}\}|\Gamma {\rangle }_{AC{E}_{B}{E}_{C}}\\  & = & \sqrt{{\eta }_{BC}{\mathscr{N}}({\eta }_{AB})}\{{\lambda }_{0}{V}_{p1}^{\ast }|0{\rangle }_{A}\otimes |{\phi }_{0}^{(B)}{\rangle }_{C}\\  &  & +\,{\mu }_{0}{V}_{p7}^{\ast }|{e}_{+}^{(A\to B)}{\rangle }_{A}\otimes |{\phi }_{0}^{(B)}{\rangle }_{C}\\  &  & +\,{\nu }_{0}{V}_{p13}^{\ast }|{e}_{+}^{(A\to B)}{\rangle }_{A}\otimes |{\phi }_{0}^{(B)}{\rangle }_{C}\\  &  & +\,{\lambda }_{1}{V}_{p2}^{\ast }|1{\rangle }_{A}\otimes |{\phi }_{1}^{(B)}{\rangle }_{C}+{\mu }_{1}{V}_{p8}^{\ast }|{e}_{+}^{(A\to B)}{\rangle }_{A}\otimes |{\phi }_{1}^{(B)}{\rangle }_{C}\\  &  & +\,{\nu }_{1}{V}_{p14}^{\ast }|{e}_{+}^{(A\to B)}{\rangle }_{A}\otimes |{\phi }_{1}^{(B)}{\rangle }_{C}\}\\  &  & +\,\sqrt{\frac{\mathrm{(1}-{\eta }_{BC}){\mathscr{N}}({\eta }_{AB})}{2}}\{{\lambda }_{0}{V}_{p3}^{\ast }|0{\rangle }_{A}\otimes |0{\rangle }_{C}\\  &  & +\,{\mu }_{0}{V}_{p9}^{\ast }|{e}_{+}^{(A\to B)}{\rangle }_{A}\otimes |0{\rangle }_{C}\\  &  & +\,{\nu }_{0}{V}_{p15}^{\ast }|{e}_{+}^{(A\to B)}{\rangle }_{A}\otimes |0{\rangle }_{C}\\  &  & +\,{\lambda }_{1}{V}_{p4}^{\ast }|1{\rangle }_{A}\otimes |0{\rangle }_{C}+{\mu }_{1}{V}_{p10}^{\ast }|{e}_{+}^{(A\to B)}{\rangle }_{A}\otimes |0{\rangle }_{C}\\  &  & +\,{\nu }_{1}{V}_{p16}^{\ast }|{e}_{+}^{(A\to B)}{\rangle }_{A}\otimes |0{\rangle }_{C}\\  &  & +{\lambda }_{0}{V}_{p5}^{\ast }|0{\rangle }_{A}\otimes |1{\rangle }_{C}+{\mu }_{0}{V}_{p11}^{\ast }|{e}_{+}^{(A\to B)}{\rangle }_{A}\otimes |1{\rangle }_{C}\\  &  & +\,{\nu }_{0}{V}_{p17}^{\ast }|{e}_{+}^{(A\to B)}{\rangle }_{A}\otimes |1{\rangle }_{C}\\  &  & +\,{\lambda }_{1}{V}_{p6}^{\ast }|1{\rangle }_{A}\otimes |1{\rangle }_{C}+{\mu }_{1}{V}_{p12}^{\ast }|{e}_{+}^{(A\to B)}{\rangle }_{A}\otimes |1{\rangle }_{C}\\  &  & +\,{\nu }_{1}{V}_{p18}^{\ast }|{e}_{+}^{(A\to B)}{\rangle }_{A}\otimes |1{\rangle }_{C}\}.\end{array}\end{array}$$

Finally, to obtain ${P}_{CE}(k,p)$, we can evaluate ${I}_{A}\otimes \langle {\beta }_{k}{|}_{C}\otimes {\langle {\pi }_{p}{|}_{{E}_{B}{E}_{C}}|\Gamma \rangle }_{AC{E}_{B}{E}_{C}}$ as follows:

45 $$\begin{array}{l}({I}_{A}\otimes {\langle {\beta }_{k}{|}_{C}\otimes \langle {\pi }_{p}{|}_{{E}_{B}{E}_{C}})|\varGamma \rangle }_{AC{E}_{B}{E}_{C}}\\ \begin{array}{rcl} & = & \{{I}_{A}\otimes \langle {\beta }_{k}{|}_{C}\otimes \mathop{\sum }\limits_{q=1}^{18}\,{V}_{pq}^{\ast }\langle q{|}_{{E}_{B}{E}_{C}}\}|\varGamma {\rangle }_{AC{E}_{B}{E}_{C}}\\  & = & \sqrt{{\eta }_{BC}{\mathscr{N}}({\eta }_{AB})}\{{\lambda }_{0}{V}_{p1}^{\ast }{\delta }_{k0}|0{\rangle }_{A}\\  &  & +\,{\mu }_{0}{V}_{p7}^{\ast }{\delta }_{k0}|{e}_{+}^{(A\to B)}{\rangle }_{A}+{\nu }_{0}{V}_{p13}^{\ast }{\delta }_{k0}|{e}_{+}^{(A\to B)}{\rangle }_{A}\\  &  & +\,{\lambda }_{1}{V}_{p2}^{\ast }{\delta }_{k1}|1{\rangle }_{A}+{\mu }_{1}{V}_{p8}^{\ast }{\delta }_{k1}|{e}_{+}^{(A\to B)}{\rangle }_{A}\\  &  & +\,{\nu }_{1}{V}_{p14}^{\ast }{\delta }_{k0}|{e}_{+}^{(A\to B)}{\rangle }_{A}\}\\  &  & +\,\sqrt{\frac{\mathrm{(1}-{\eta }_{BC}){\mathscr{N}}({\eta }_{AB})}{2}}\{{\lambda }_{0}{V}_{p3}^{\ast }\sqrt{\frac{1+s{\prime} }{2}}|0{\rangle }_{A}\\  &  & +\,{\mu }_{0}{V}_{p9}^{\ast }\sqrt{\frac{1+s{\prime} }{2}}|{e}_{+}^{(A\to B)}{\rangle }_{A}+{\nu }_{0}{V}_{p15}^{\ast }\sqrt{\frac{1+s{\prime} }{2}}|{e}_{+}^{(A\to B)}{\rangle }_{A}\\  &  & +\,{\lambda }_{1}{V}_{p4}^{\ast }\sqrt{\frac{1+s{\prime} }{2}}|1{\rangle }_{A}+{\mu }_{1}{V}_{p10}^{\ast }\sqrt{\frac{1+s{\prime} }{2}}|{e}_{+}^{(A\to B)}{\rangle }_{A}\\  &  & +\,{\nu }_{1}{V}_{p16}^{\ast }\sqrt{\frac{1+s{\prime} }{2}}|{e}_{+}^{(A\to B)}{\rangle }_{A}\\  &  & +\,{\lambda }_{0}{V}_{p5}^{\ast }{(-\mathrm{1)}}^{k}\sqrt{\frac{1-s{\prime} }{2}}|0{\rangle }_{A}+{\mu }_{0}{V}_{p11}^{\ast }{(-\mathrm{1)}}^{k}\sqrt{\frac{1-s{\prime} }{2}}|{e}_{+}^{(A\to B)}{\rangle }_{A}\\  &  & +\,{\nu }_{0}{V}_{p17}^{\ast }{(-\mathrm{1)}}^{k}\sqrt{\frac{1-s{\prime} }{2}}|{e}_{+}^{(A\to B)}{\rangle }_{A}\\  &  & +\,{\lambda }_{1}{V}_{p6}^{\ast }{(-\mathrm{1)}}^{k}\sqrt{\frac{1-s{\prime} }{2}}|1{\rangle }_{A}\\  &  & +\,{\mu }_{1}{V}_{p12}^{\ast }{(-\mathrm{1)}}^{k}\sqrt{\frac{1-s{\prime} }{2}}|{e}_{+}^{(A\to B)}{\rangle }_{A}\\  &  & +\,{\nu }_{1}{V}_{p18}^{\ast }{(-\mathrm{1)}}^{k}\sqrt{\frac{1-s{\prime} }{2}}|{e}_{+}^{(A\to B)}{\rangle }_{A}\}\mathrm{}.\end{array}\end{array}$$

In Eqs. (44) and (45), ${\delta }_{ij}$ is Kronecker delta. And, ${\lambda }_{i}$, ${\mu }_{i}$, and ${\nu }_{i}$ are defined as follows:

$${\lambda }_{i}=\sqrt{{\eta }_{AB}{q}_{i}{\alpha }_{i}},\,{\mu }_{i}=\frac{1}{2}\sqrt{\frac{(1-{\eta }_{AB}){\alpha }_{i}}{1+s}},\,{\nu }_{i}=\frac{1}{2}\sqrt{\frac{(1-{\eta }_{AB}){\alpha }_{i}}{1-s}}.$$

### Simulation method to search for critical channel efficiency (${\bar{\eta }}_{crit}$)

When the unitary transformation $V$ and overlap $s$ are determined, the secret key rate is expressed as follows:

$${K}_{AC:{E}_{B}{E}_{C}}=\text{max}\{0,f(\bar{\eta })\}.$$

**Figure 17:**
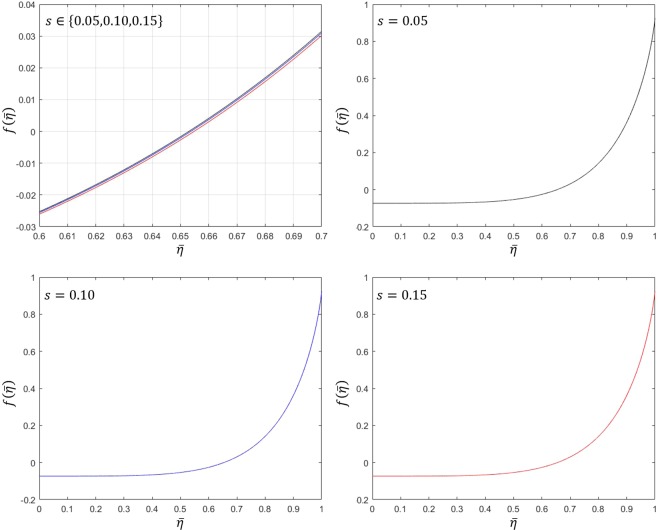
The graph of $f(\bar{\eta })$. In these graphs, the solid black line, the solid blue line, and the solid red line denote the cases of $s=0.05,0.10$, and 0.15, respectively. These graphs show that $f(\bar{\eta })$ is a monotonically increasing function of $\bar{\eta }$. Because $\bar{\eta }$ is a noise strength of channel, these graphs tell that noise of channel can make a bad influence on the secret key rate.

Here, $f(\eta )=I(A:C)-I(C:E)$ is a function of the single variable $\bar{\eta }$. In Fig. 17, when $V$ is an identity and $s\in \{0.05,0.10,0.15\}$, $f(\bar{\eta })$ is a monotonically increasing function. Therefore, in the region of $\bar{\eta }\in [0,1]$, there is only one value of ${\bar{\eta }}_{crit}$ satisfying $f({\bar{\eta }}_{crit})=0$. And, if $\bar{\eta } > {\bar{\eta }}_{crit}$, because of $f(\bar{\eta }) > 0$, the secret key rate becomes nonzero. In this case, ${\bar{\eta }}_{crit}$ can be obtained by a bisection method^49^.
